# Metapopulation heterogeneities in host mobility, productivity, and immunocompetency always increase virulence and infectiousness

**DOI:** 10.1073/pnas.2309272121

**Published:** 2024-12-19

**Authors:** Masato Sato, Ulf Dieckmann, Akira Sasaki

**Affiliations:** ^a^Department of Evolutionary Studies of Biosystems, The Graduate University for Advanced Studies, SOKENDAI, Hayama, Kanagawa 240-0193, Japan; ^b^Bioproduction Research Institute, National Institute of Advanced Industrial Science and Technology, Tsukuba, Ibaraki 305-8561, Japan; ^c^Complexity Science and Evolution Unit, Okinawa Institute of Science and Technology Graduate University (OIST), Tancha, Onna, Kunigami, Okinawa 904-0495, Japan; ^d^Research Center for Integrative Evolutionary Science, The Graduate University for Advanced Studies, SOKENDAI, Hayama, Kanagawa 240-0193, Japan; ^e^Advancing Systems Analysis Program & Evolution and Ecology Program, International Institute for Applied Systems Analysis (IIASA), Laxenburg 2361, Austria

**Keywords:** epidemiology, adaptive dynamics, virulence, metapopulation, heterogeneity

## Abstract

The epidemiology and evolution of diseases unfold in populations that are rarely homogeneous. Instead, hosts infected by pathogens often form metapopulations, in which local populations connected by the movement of hosts experience different demographic and epidemiological conditions. Here, we develop a general theory of the evolution of pathogens in heterogeneous metapopulations. We reveal the following key insights into the evolution of pathogen virulence and infectiousness: (1) When the mobility (movement rate), productivity (birth rate and carrying capacity), or immunocompetency (immunity-loss rate) differ among local populations, this variance always increases pathogen virulence and infectiousness (2) The increment of pathogen virulence caused by such heterogeneity is approximately proportional to the variance of the corresponding heterogeneous local conditions (3) This increment can be expressed as the covariance between the local selection pressures and the local reproductive values experienced by the pathogen (4) The reason why heterogeneity always increases pathogen virulence is explained by the positive correlation of local selection pressures with reproductive values (5) Combinations of multiple independent heterogeneities further increase virulence and infectiousness, even more so when their covariances are positive. Our key findings robustly hold for different epidemiological frameworks – including SI, SIS, SIR, and SIRS models, with both density- and frequency-dependent transmission as well as with superinfection. They provide insights into the risks of growing pathogen infectiousness in a world in which heterogeneity – caused, e.g., by the concentration of human populations in urban areas – is rising.

## Significance

Infectious diseases are spreading on an unprecedented scale through the increased mobility of their hosts. Our general theory reveals that, when host populations experiencing different local conditions are linked by movement networks, more virulent and infectious pathogens evolve than in a corresponding homogeneous population. We show that the greater the variation in host mobility, productivity, and immunocompetency, the more virulent and infectious pathogens become. Worse still, when multiple such heterogeneities are positively correlated, the evolution of higher virulence is boosted: For example, for positively correlated heterogeneities in movement and birth rates, the evolved virulence increases to nearly 200%. Our theory provides insights into aggravating risks for the emergence of more virulent pathogens in our highly connected heterogeneous world.

The evolution of pathogens has historically received attention in a wide range of scientific fields, including epidemiology, demography, and evolutionary ecology. To confront rapidly evolving pathogens, such as SARS-CoV2 spreading among humans since 2019, understanding pathogen evolution is just as important as understanding pathogen demography. During the past few decades, the evolution of pathogen virulence – typically measured in terms of pathogen-induced host mortality – has attracted mounting attention, leading to numerous theoretical and empirical studies addressing why and how pathogens improve their fitness by exploiting, harming, and killing their hosts. A key insight is that pathogen virulence often evolves so as to maximize a pathogen’s basic reproduction number, defined as the expected number of secondary infections resulting from a primary infection in a fully susceptible host population (1, 2). This classical theory enables an understanding of how pathogen virulence adapts under given tradeoffs with other epidemiological parameters, such as the rates of transmission and recovery – its most prominent prediction being that of an intermediate evolutionarily optimal virulence under a saturating tradeoff between virulence and transmission (1, 3–7). Since its inception, this approach has been extended to cover a wide range of ecological and epidemiological factors (7–9).

Despite the large number of theoretical studies describing the evolution of virulence and the aforementioned extensions to many ecological and epidemiological contexts, the majority of them have been developed under the simplifying assumption of homogeneous host populations (but see refs. 10, 11 for class-structured models and refs. 12, 13 for spatially structured models). In nature, however, host populations are almost invariably structured and heterogeneous. According to the metapopulation concept (14–17), many ecological systems can be construed as regional groups of interconnected local populations. The local populations serving as the components of such metapopulations are usually facing different local conditions. Salient differences may involve external environmental heterogeneities, differentiated community compositions, or phenotypic polymorphisms of a focal species and the multiple species with which it interacts (18). External environmental heterogeneities in metapopulations are well documented empirically (19): Two examples picked out of many include spatial heterogeneity in the carrying capacities of deer populations in Japan (20) and spatial heterogeneity in the per capita growth rates of goshawk populations in Germany (21). The movements of individuals connecting local populations are also widely found to be heterogeneous, and an imbalance of incoming and outgoing movements between local populations generates source-sink structure, with local populations having more emigrants than immigrants called sources and local populations having more immigrants than emigrants called sinks: Two examples, again picked out of many, include heterogeneous gene flow among local yeast populations in New Zealand (22) and heterogeneous pollen dispersal across the habitat range of Sitka spruce along the North-American West Coast (23). Such heterogeneities among the local populations forming metapopulations have been the focus of many studies in ecology and epidemiology examining how they affect ecological stability and diversity and how they influence local adaptations (24).

Despite a broad interest in the ecological consequences of metapopulation heterogeneities, their implications for life-history evolution have not yet been equally well explored, especially in the context of evolutionary epidemiology. Previous studies on the evolution of hosts and pathogens in heterogeneous metapopulations (25–27) have mainly focused on local adaptation, examining diversification among local populations as hosts and pathogens adapt to local environmental conditions. Such a limited understanding of the evolution of hosts and pathogens in heterogeneous metapopulations makes it difficult to test various hypotheses proposed by experimental and field biologists (28, 29). An important open question is how the local selection pressures potentially causing local adaptations in heterogeneous metapopulations are mingled, integrated, and reconciled at the metapopulation level, and how this molds the resultant evolutionary process.

Here, we construct a general theory of the evolution of pathogen virulence and infectiousness in heterogeneous host metapopulations. Building on previous theoretical studies of evolution in structured populations using the Price equation for predicting invasion fitness in terms of covariances between local trait values and local-reproductive-value-weighted local fitness values summed over population-structure classes (10, 30–34), we use perturbation analyses in heterogeneity magnitudes (35) to derive simple results and testable quantitative predictions for understanding the effects of various types of metapopulation heterogeneities on the evolution of pathogen virulence. On this basis, we show that heterogeneities in the movement, birth, carrying capacity, and immunity loss of hosts *always* promote the evolutionary emergence of more virulent and infectious pathogens.

## Results

**Fig. 1.**
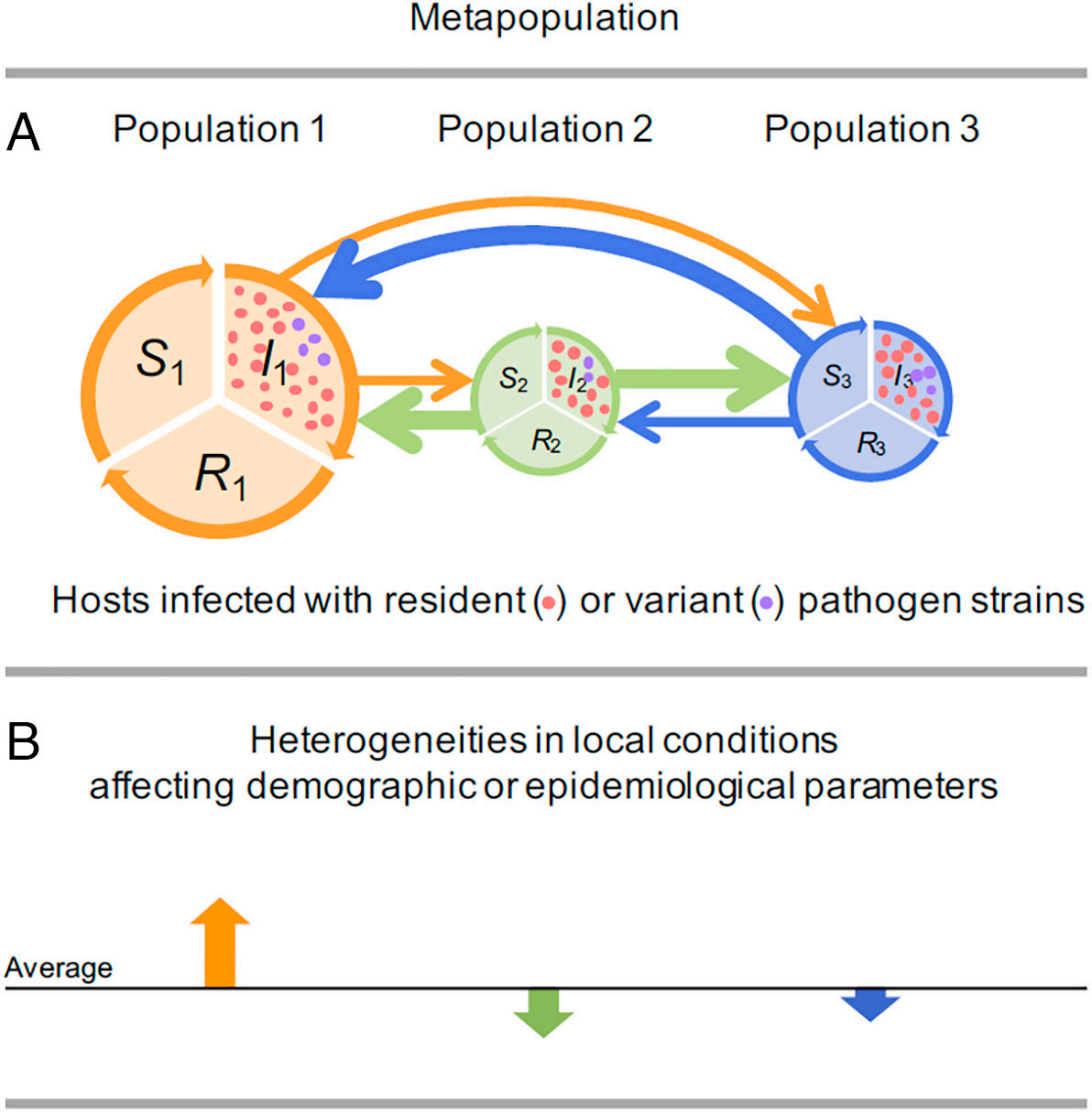
Schematic illustration of heterogeneous host metapopulations with evolving pathogen virulence and infectiousness. (*A*) Host populations (indicated by the orange, green, and blue circles) coupled by movement (indicated by arrows connecting the circles). Each population may comprise susceptible hosts (with densities $S_{1}$, $S_{2}$, $S_{3}$), infected hosts (with densities $I_{1}$, $I_{2}$, $I_{3}$), and recovered-and-immune hosts (with densities $R_{1}$, $R_{2}$, $R_{3}$). Hosts may be infected by pathogen strains differing in their virulence and infectiousness (red: the resident strain, purple: a variant strain). By analyzing when the latter hosts outcompete the former ones, we determine the metapopulation selection pressure on virulence. (*B*) Heterogeneous local conditions in a metapopulation of hosts may cause demographic and epidemiological parameters to vary among local host populations.

We consider SIRS epidemiological dynamics in which hosts are classified into susceptible, infected, and recovered classes in a heterogeneous metapopulation (Eq. **4** in Model and Method and Fig. 1). The hosts’ movement rates between local populations, as well as their birth rates, carrying capacities, and immunity-loss rates in these local populations, are assumed to vary randomly from population to population around their metapopulation averages Eq. **6**. On this basis, we examine how the pathogen virulence evolving under a virulence-infectiousness tradeoff depends on the degree of metapopulation heterogeneity.

Specifically, we investigate how the evolved virulence of a pathogen is determined by a metapopulation’s degree of heterogeneity. We refer to the evolved virulence as evolutionarily stable strategy (ESS) virulence, as it emerges as an ESS. All mathematical notations used below are listed in Table 1. We first introduce the metapopulation heterogeneity. The movement rate $m_{\mathrm{ij}}$ from population j to i varies around its metapopulation average $m_{0}/n=E\left[m_{\mathrm{ij}}\right]$ as

[1a] $$m_{\mathrm{ij}}=m_{0}/n+\epsilon m_{\mathrm{ij}}^{\prime },$$

**Table 1.** Model variables and parameters

Symbol	Description	Type, definition, or default value
$S_{i}$	Density of susceptible hosts in populationi	Variable
$I_{i}$	Density of infected hosts in populationi	Variable
$R_{i}$	Density of recovered-and-immune hosts in populationi	Variable
$N_{i}$	Total density of hosts in populationi	$S_{i}+I_{i}+R_{i}$
$m_{\mathrm{ij}}$	Movement rate of hosts from populationjtoi	Heterogeneous
$m_{0}$	Average movement rate of hosts across metapopulation	0.6
$T_{i}$	Net movement rate of hosts to populationi	$\Sigma_{j}m_{\mathrm{ij}}-\Sigma_{j}m_{\mathrm{ji}}$
$r_{i}$	Intrinsic growth rate of hosts in populationi	Heterogeneous
$r_{0}$	Average intrinsic growth rate of hosts across metapopulation	0.5
$K_{i}$	Carrying capacity of hosts in populationi	Heterogeneous
$K_{0}$	Average carrying capacity of hosts across metapopulation	40
$\nu_{i}$	Immunity-loss rate of hosts in populationi	Heterogeneous
$\nu_{0}$	Average immunity-loss rate of hosts across metapopulation	0.05
α	Virulence of pathogens	Evolving
β	Transmission rate of pathogens	$a\sqrt{\alpha }$
a	Coefficient of the transmission rate of pathogens	5.0
η	Recovery rate of hosts to susceptible state	0.05
γ	Recovery rate of hosts to immune state	0.1
μ	Natural mortality rate of hosts	0.001
n	Number of local populations	20
ϵ	Coefficient of variation of metapopulation heterogeneity	0.1
${\hat{V}}_{x}$	Relative variance of parameterx	$\mathrm{Var}\left(x\right)/x_{0}^{2}$
${\hat{C}}_{\mathrm{xy}}$	Relative covariance of parametersxandy	$Cov\left(x,y\right)/\left(x_{0}y_{0}\right)$
$x_{i}$	Local value of heterogeneous parameterxin populationi	Heterogeneous
$v_{i}$	Local reproductive value in populationi	
$s_{i}$	Local selection pressure in populationi	
$R_{0}$	Basic reproduction number of pathogen	
λ	Per capita growth rate of infected hosts	
$E_{x}$	Elasticity of ESS virulence to metapopulation heterogeneity in parameterx	
$Q_{x}$	Elasticity of equilibrium local densities of susceptible hosts to metapopulation heterogeneity in parameterx	
Θ	Elasticity of ESS virulence to equilibrium local densities of susceptible hosts	
$X^{*}$	Equilibrium value ofX	
$\hat{X}$	Value ofXfor a variant pathogen strain	
$X_{0}$	Average ofXacross metapopulation	
$\bar{X}$	Target average ofXacross metapopulation	
$X^{\prime }$	Coefficient of first-order deviation ofXcaused by metapopulation heterogeneity	

where the $m_{\mathrm{ij}}^{\prime }$ have mean 0. Here, $m_{0}$ is the total movement rate in the corresponding homogeneous metapopulation: An individual moves from one population (j) to another with probability $m_{0}$ per unit time, and one of the n populations (i) is chosen as the destination with equal probability 1/n. The second term $\epsilon m_{\mathrm{ij}}^{\prime }$ is the deviation from homogeneous movement. The birth rate $r_{i}$, carrying capacity $K_{i}$, and immunity-loss rate $\nu_{i}$ also vary around their metapopulation averages $r_{0}$, $K_{0}$, and $\nu_{0}$ as

[1b] $$x_{i}=x_{0}+\epsilon x_{i}^{\prime },$$

where x can stand for r, K, or ν, the $x_{i}^{\prime }$ have mean 0, and $x_{0}=E\left[x_{i}\right]$. The positive constant ϵ determines the degree of metapopulation heterogeneity. Throughout this paper, we assume a positive and saturating tradeoff between the infection rate β of pathogens and the pathogen virulence α,

[1c] $$\frac{d\beta }{d\alpha }>0\;\mathrm{and}\;\frac{d^{2}\beta }{d\alpha^{2}}<0.$$

### Metapopulation Heterogeneities in Movement, Birth, Carrying Capacity, and Immunity Loss Always Increase Virulence.

We compare a metapopulation with heterogeneities in movement, birth, carrying capacity, and immunity loss with a corresponding homogeneous metapopulation in which all these local conditions have the same means while being uniform across local populations. Our central finding is that the ESS virulence in a heterogeneous metapopulation is always higher than that in a corresponding homogeneous metapopulation. We start by illustrating this general result numerically before demonstrating it analytically further below.

**Fig. 2.**
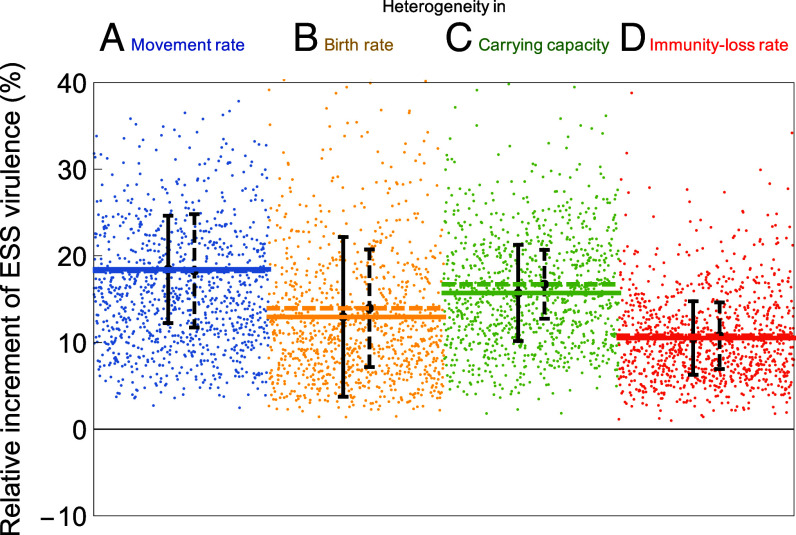
Relative increments of ESS virulence caused by heterogeneities in randomly generated metapopulations. The increments are measured in comparison with the ESS virulence in the corresponding homogeneous metapopulation. The figure compares numerical and analytical results for four different types of heterogeneity: in (*A*) movement rates (blue), (*B*) birth rates (yellow), (*C*) carrying capacities (green), and (*D*) immunity-loss rates (red). For each of these, the panels show the relative increments of ESS virulence (dots) obtained through the numerical evolution of pathogen virulence in each of 1,000 randomly generated metapopulations, the mean ESS virulence obtained through this numerical evolution (continuous lines), and the mean ESS virulence predicted by our analytical theory (dashed lines) according to Eq. **3b**. Black vertical bars show the SD of the distributions of ESS virulence obtained by numerical evolution (continuous bars) and predicted by our analytical theory (dashed bars) according to *SI Appendix*, section S3. All metapopulations comprise n=20 local populations. To facilitate comparison, the degree of heterogeneity, measured by the target coefficient of variation, $\epsilon =\sigma_{x}/\bar{x}$, where $\bar{x}$ is the target mean and $\sigma_{x}$ is the target SD, equals 10% for all four types x=m, r, K, and ν of heterogeneity. All parameter values are as shown in Table 1.

Fig. 2 shows how the mean ESS virulence evolves in response to metapopulation heterogeneities, for heterogeneous variations in movement rates (Fig. 2*A*, blue), birth rates (Fig. 2*B*, yellow), carrying capacities (Fig. 2*C*, green), or immunity loss rates (Fig. 2*D*, red) characterized by average coefficients of variation of 10%, i.e., by relative variances of 1%. The resultant mean changes in ESS virulence range from 10.5% to 19.1%, measured in terms of the increments relative to the ESS virulence in the absence of metapopulation heterogeneities. Hence, the corresponding evolutionary elasticities range from 10.5%/1% = 10.5 for metapopulation heterogeneity in immunity loss to 19.1%/1% = 19.1 for metapopulation heterogeneity in movement. According to the classification of magnitudes of elasticities, this means that the response of ESS virulence to all considered metapopulation heterogeneities is very highly elastic (36), i.e., even small relative variances go a long way in causing large relative changes in ESS virulence. Crucially, as predicted by our theoretical analysis, the ESS virulence always rises.

Fig. 2 also shows results for 1,000 metapopulations randomly generated for each type of heterogeneity. Sampling variance among these replicates causes the realized metapopulation heterogeneities to be scattered around a mean coefficient of variation of 10%. The resultant relative increments of ESS virulence, shown by the dots in Fig. 2, are scattered accordingly. The numerically realized means and SD (thick continuous horizontal lines and vertical error bars) of these distributions closely match their analytically predicted values (thick dashed horizontal lines and vertical error bars), with small differences originating from the finite number of replicates and the leading-order approximation employed in (parts of) our theoretical analysis.

Beyond these means and SD, our theory also allows predicting the shape of the distributions of relative increases in ESS virulence, which, for metapopulations comprising n local populations with normally distributed heterogeneities, can be demonstrated to follow chi-squared distributions with n-1 degrees of freedom (*SI Appendix*, section S4 and Fig. S4). Hence, the coefficient of variation of ESS virulence is predicted to equal $\sqrt{2/\left(n-1\right)}$ (*SI Appendix*, section S6), showing that, while the mean virulence does not change with n, the SD in Fig. 2 are approximately inversely proportional to the square root of the number n of local populations in the metapopulation. This implies that the spread of ESS virulences becomes negligible for large metapopulations, i.e., in the limit of metapopulations comprising increasingly many local populations.

Our analytical predictions can be made even more accurate by taking into account higher-order effects, which, as described further below, enable us to understand – and narrow – the small differences between the numerical results and analytical predictions shown in Fig. 2.

### Why Do Metapopulation Heterogeneities Always Increase Virulence?

We now explain why metapopulation heterogeneity in the considered local conditions always increases ESS virulence (Fig. 2). This key result can be understood in three steps, as follows.

First, we find that, if the degree ϵ of metapopulation heterogeneity is small, the increment of the selection pressure s(α) on the pathogen virulence α due to metapopulation heterogeneity is given by the covariance of local reproductive values and local selection pressures,

[2a] $$s\left(\alpha \right)-s_{0}\left(\alpha \right)=\mathrm{Cov}\left(v_{i},s_{i}\right),$$

up to second order in ϵ, where $s_{0}\left(\alpha \right)$ is the selection pressure in the corresponding homogeneous metapopulation, $v_{i}$ is the local reproductive value of population i, describing the relative contribution of population i to pathogen evolution in the metapopulation, and $s_{i}$ is the local selection pressure on pathogen virulence in population i (see *SI Appendix*, section S1 for the derivation of this equation and *SI Appendix*, section S2 for the derivation of $s\left(\alpha \right)$).

Second, we show that the deviations $s_{i}-s_{0}$ of local selection pressures from their metapopulation average and the deviations $v_{i}-v_{0}$ of local reproductive values from their metapopulation average are both proportional to the deviations $S_{i}^{*}-S_{0}^{*}$ of the local equilibrium densities of susceptible hosts from their metapopulation average,

[2b] $$s_{i}-s_{0}=\frac{d\beta }{d\alpha }(S_{i}^{*}-S_{0}^{*}),$$

**Fig. 3.**
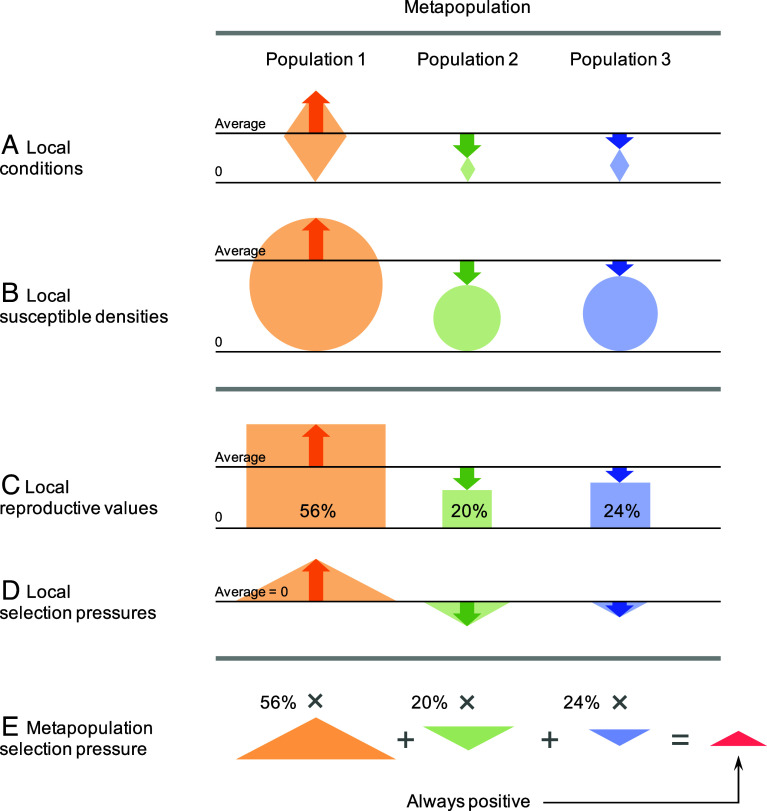
Schematic explanation of the reason why metapopulation heterogeneities in local conditions (movement rates, birth rates, carrying capacities, and immunity-loss rates) always raise the ESS virulence and infectiousness of pathogens. (*A*) The local conditions may vary among local host populations (as indicated by the sizes of the orange, green, and blue diamonds) and thereby deviate (as indicated by the correspondingly colored arrows) from their metapopulation averages (as indicated by the black horizontal lines). Panels (*B*–*D*) illustrate the corresponding variations in local equilibrium densities of susceptible hosts, local reproductive values, and local selection pressures, caused by these variations in local conditions. Crucially, the deviations in all four of these quantities from their metapopulation averages follow a pattern of positive proportionality (as indicated by the congruence among, respectively, all orange arrows, all green arrows, and all blue arrows). (*E*) The metapopulation selection pressure on the virulence and infectiousness of pathogens (as indicated by the red triangle) is given by the sum of local selection pressures (as indicated by the sizes of the orange, green, and blue triangles) weighted by the relative local reproductive values (as indicated by the percentages). The positive proportionality between the deviations in local reproductive values shown in (*C*) and the deviations in local selection pressures shown in (*D*) implies that local populations selecting for increased (decreased) virulence and infectiousness always make above-average (below-average) contributions to the metapopulation selection pressure on virulence and infectiousness, which is therefore always positive.

[2c] $$v_{i}-v_{0}=\frac{\beta }{m_{0}}\left(S_{i}^{*}-S_{0}^{*}\right),$$

up to first order in ϵ (see *SI Appendix*, section S2.3 for the derivations of these equations). Thus, in local populations that have a higher equilibrium density of susceptible hosts than the metapopulation average ($S_{i}^{*}>S_{0}^{*}$), the local selection pressure toward increased virulence not only has above-average strength ($s_{i}>s_{0}$) but the local contribution of such populations to virulence evolution is above-average too ($v_{i}>v_{0}$). Conversely, in local populations that have a lower equilibrium density of susceptible hosts than the metapopulation average ($S_{i}^{*}<S_{0}^{*}$), the local selection pressure toward increased virulence not only has below-average strength ($s_{i}<s_{0}$) but the local contribution of such populations to virulence evolution is below-average too ($v_{i}<v_{0}$). In other words, local populations favoring higher virulence contribute more to virulence evolution in the metapopulation than local populations favoring lower virulence. While the speed and direction of evolution in a metapopulation is determined by the summation of local selection pressures, this sum is weighted by local reproductive values. As illustrated in Fig. 3, the positive proportionality of deviations of local selection pressures and local reproductive values from their metapopulation averages thus leads to the always increasing effect of metapopulation heterogeneity on virulence evolution. This qualitative reasoning can be made quantitative, as shown in our next and last step.

Third, we substitute Eq. **2b** into the covariance of local selection pressures and reproductive values,

[2d] $$\begin{array}{c} \mathrm{Cov}\left(v_{i},s_{i}\right)=\frac{1}{n}\sum_{i}\left(v_{i}-v_{0}\right)\left(s_{i}-s_{0}\right)\\ \;=\frac{\beta }{m_{0}}\frac{d\beta }{d\alpha }\frac{1}{n}\sum_{i}{\left(S_{i}^{*}-S_{0}^{*}\right)}^{2}=\frac{\beta }{m_{0}}\frac{d\beta }{d\alpha }\mathrm{Var}\left(S_{i}^{*}\right). \end{array}$$

Thus, as the variance of local equilibrium densities of susceptible hosts is always positive, the covariance of local reproductive values and local selection pressures is always positive too. Therefore, the increment of the selection pressure on pathogen virulence due to metapopulation heterogeneity Eq. **2a** is always positive,

[2e] $$s\left(\alpha \right)-s_{0}\left(\alpha \right)=\frac{\beta }{m_{0}}\frac{d\beta }{d\alpha }\mathrm{Var}\left(S_{i}^{*}\right)>0.$$

This implies that $s\left(\alpha \right)>s_{0}\left(\alpha \right)$, i.e., for any virulence α, the selection pressure toward increased virulence is always higher in a heterogeneous metapopulation than in the corresponding homogeneous metapopulation. Hence, the ESS virulence $\alpha^{*}$ in a heterogeneous metapopulation is always larger than the ESS virulence $\alpha_{0}^{*}$ in the corresponding homogeneous metapopulation, which satisfies $s_{0}\left(\alpha_{0}^{*}\right)=0$, because $s\left(\alpha_{0}^{*}\right)>0$. Thus, metapopulation heterogeneities always increase the ESS virulence, as we have seen numerically in the previous section.

### Virulence Increases in Proportion to Variance in Local Conditions.

We now investigate how the increment of ESS virulence is determined by a metapopulation’s degree of heterogeneity. As a simple analytical result with a not so simple derivation (*SI Appendix*, section S2), we find that the increment $\delta \alpha^{*}=\alpha^{*}-\alpha_{0}^{*}$ of the ESS virulence $\alpha^{*}$ caused by metapopulation heterogeneity, relative to the ESS virulence $\alpha_{0}^{*}$ in the absence of metapopulation heterogeneity, is proportional to the variance $\mathrm{Var}\left(S_{i}^{*}\right)$ of the local equilibrium densities of susceptible hosts, with a positive proportionality constant Θ specified below,

[3a] $$\frac{\alpha^{*}-\alpha_{0}^{*}}{\alpha_{0}^{*}}=\Theta \frac{\mathrm{Var}\left(S_{i}^{*}\right)}{{\left(S_{0}^{*}\right)}^{2}}.$$

Here, $\mathrm{Var}\left(S_{i}^{*}\right)/E{\left(S_{i}^{*}\right)}^{2}=\mathrm{Var}\left(S_{i}^{*}\right)/{\left(S_{0}^{*}\right)}^{2}$ is the squared coefficient of variation of the equilibrium densities $S_{i}^{*}$ of susceptible hosts across the metapopulation, and ${\left(-d^{2}\beta /d\alpha^{2}\right)}^{-1}$ $\left(\beta /\alpha m_{0}\right){|}_{\alpha ={\alpha^{*}}_{0}}>0$ is a constant that is independent of metapopulation heterogeneity and always positive because the tradeoff between transmission and virulence is assumed to be concave ($d^{2}\beta /d\alpha^{2}<0$). Since variances cannot be negative, this implies that the considered metapopulation heterogeneities never decrease the ESS virulence.

The same principle applies also in terms of the variability of local conditions $x_{i}$ across the metapopulation: With an additional positive proportionality constant $Q_{x}=q_{x}^{2}>0$, where $q_{x}$ is the elasticity of the local equilibrium densities $S_{i}^{*}$ of susceptible hosts to the local conditions $x_{i}$, as defined in Eq. **3c** below, the relative increment of ESS virulence is proportional to the variance $\mathrm{Var}(x_{i})$ of local conditions $x_{i}$,

[3b] $$\frac{\alpha^{*}-\alpha_{0}^{*}}{\alpha_{0}^{*}}=\Theta Q_{x}\frac{\mathrm{Var}\left(x_{i}\right)}{x_{0}^{2}},$$

where $x_{i}$ can stand for the local birth rates, carrying capacities, immunity-loss rates, or net movement inflows varying across the heterogeneous metapopulation and $\mathrm{Var}\left(x_{i}\right)/x_{0}^{2}=\mathrm{Var}\left(x_{i}\right)/E{\left(x_{i}\right)}^{2}$ is the squared coefficient of variation, ${\left({\mathrm{CV}}_{x}\right)}^{2}$, of the local conditions $x_{i}$. This simply follows from Eq. **3a** and the fact that the relative deviation of local equilibrium densities of susceptible hosts from their metapopulation average is proportional to the relative deviation of local conditions from their metapopulation average with the proportionality constant $q_{x}$,

[3c] $$\frac{S_{i}^{*}-S_{0}^{*}}{S_{0}^{*}}=q_{x}\frac{x_{i}-x_{0}}{x_{0}},$$

which implies $\mathrm{Var}\left(S_{i}^{*}\right)/{\left(S_{0}^{*}\right)}^{2}=Q_{x}\mathrm{Var}\left(x_{i}\right)/x_{0}^{2}$ with $Q_{x}=q_{x}^{2}$ as noted earlier; see *SI Appendix*, section S3 for the derivation of this equation and for analytical results for $q_{x}$ for each choice of x. If, for example, the carrying capacities $K_{i}$ of local populations i vary across the metapopulation, the relative increment of ESS virulence due to metapopulation heterogeneity is proportional to the squared coefficient of variation of local carrying capacities, ${\left({\mathrm{CV}}_{K}\right)}^{2}=\mathrm{Var}\left(K_{i}\right)/K_{0}^{2}$ (Fig. 1*C*). The same formula applies to metapopulation heterogeneities in birth rates, with ${\left({\mathrm{CV}}_{r}\right)}^{2}=\mathrm{Var}\left(r_{i}\right)/r_{0}^{2}$, in immunity-loss rates, with ${\left({\mathrm{CV}}_{\nu }\right)}^{2}=\mathrm{Var}\left(\nu_{i}\right)/\nu_{0}^{2}$, and in net movement inflows, with ${\left({\mathrm{CV}}_{T}\right)}^{2}=\mathrm{Var}\left(T_{i}\right)/m_{0}^{2}$, where $x_{i}=T_{i}=\sum_{j}\left(m_{\mathrm{ij}}-m_{\mathrm{ji}}\right)/n$ and $x_{0}=E(m_{\mathrm{ij}})=m_{0}$.

### Analytically Derived Elasticities Allow Predicting the Magnitude of Virulence Increase.

**Fig. 4.**
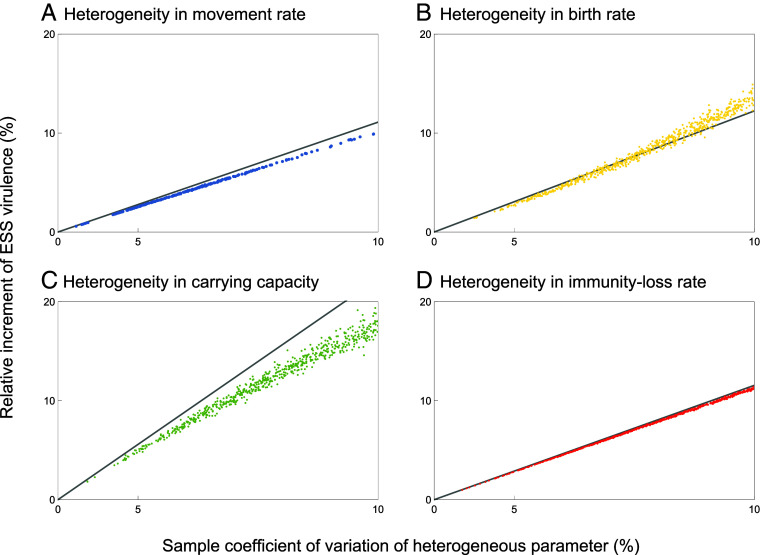
Dependence of the relative increments of ESS virulence caused by metapopulation heterogeneity on the degree of heterogeneity. The increments are measured in comparison with the ESS virulence in the corresponding homogeneous metapopulation. The figure compares numerical and analytical results for four different types of heterogeneity: in (*A*) movement rates (blue), (*B*) birth rates (yellow), (*C*) carrying capacities (green), and (*D*) immunity-loss rates (red). For each of these, the panels show on the vertical axes the relative increments of ESS virulence (dots) obtained through numerical evolution in each of 1,000 randomly generated metapopulations (created as described in the caption of Fig. 2) in comparison with the ESS virulence predicted by our analytical theory (black lines) according to Eq. **3b**. The horizontal axis is scaled quadratically.

Our prediction in Eq. **3b** that the increment of the ESS virulence is approximately proportional to the variance of local conditions across a metapopulation is corroborated in Fig. 4. In this figure, the relative increment of the ESS virulence is plotted against the coefficient of sampling variation in the parameters x describing movement (Fig. 4*A*), birth (Fig. 4*B*), carrying capacity (Fig. 4*C*), and immunity loss (Fig. 4*D*) using quadratic scaling on the horizontal axis and showing good agreement between our analytical predictions and our numerical results based on explicit evolutionary dynamics.

The proportionality constant $q_{x}$ in Eq. **3c** is the elasticity of the local equilibrium densities $S_{i}^{*}$ of susceptible hosts to the local conditions $x_{i}$. Differences in $q_{x}$ among the four parameters described by x are responsible for the differences in the responsiveness of the ESS virulence to the four different types of metapopulation heterogeneities of the same relative magnitude shown across the four panels of Fig. 2. Since the local equilibrium densities of susceptible hosts depend on many epidemiological and ecological parameters other than x, understanding how $Q_{x}$ depends on the considered types of metapopulation heterogeneities and on the considered other parameters is not easy (*SI Appendix*, section S3). Some further insights can be gained from a limiting case, in which the host growth rate r is sufficiently high: This allows us to show that, for x=T,K,ν, the elasticity $Q_{x}$ then is the higher the larger the movement rate $m_{0}$ is relative to the force of infection, $\beta I_{0}^{*}$ (*SI Appendix*, section S3.6).

In summary, the variance in the local conditions $x_{i}$ affects the variance in the local equilibrium densities $S_{i}^{*}$ of susceptible hosts according to the elasticity $Q_{x}$, the latter variance affects the ESS virulence $\alpha^{*}$ according to the elasticity Θ, and therefore, the variance in the local conditions $x_{i}$ affects the ESS virulence $\alpha^{*}$ according to the product of these two elasticities, i.e., according to the elasticity $E_{x}=\Theta Q_{x}$. According to the chain rule for elasticities, we can thus understand Θ as the elasticity of the ESS virulence to the variance of the local equilibrium densities of susceptible hosts.

It is noteworthy that our results for the expected increase of ESS virulence in heterogeneous metapopulations show no dependence on the number n of local populations. This means that the amplifying effect of metapopulation heterogeneity on ESS virulence is equally strong in metapopulations with many local populations as it is in metapopulations with few local populations. The number of local populations merely affects the variability of ESS virulence around the expected value of ESS virulence – according to a coefficient of variation of $\sqrt{2/\left(n-1\right)}$, as illustrated in Fig. 2 and derived in *SI Appendix*, section S4 – without influencing the expected virulence increment itself.

### Multiple Heterogeneities Increase Virulence According to the Sum of Their Variances and Covariances.

We find that, when the metapopulation heterogeneities we have so far considered individually are combined for a pair of parameters x and y, their impacts on ESS virulence are additive, in the sense that the relative increment of ESS virulence is given by

**Fig. 5.**
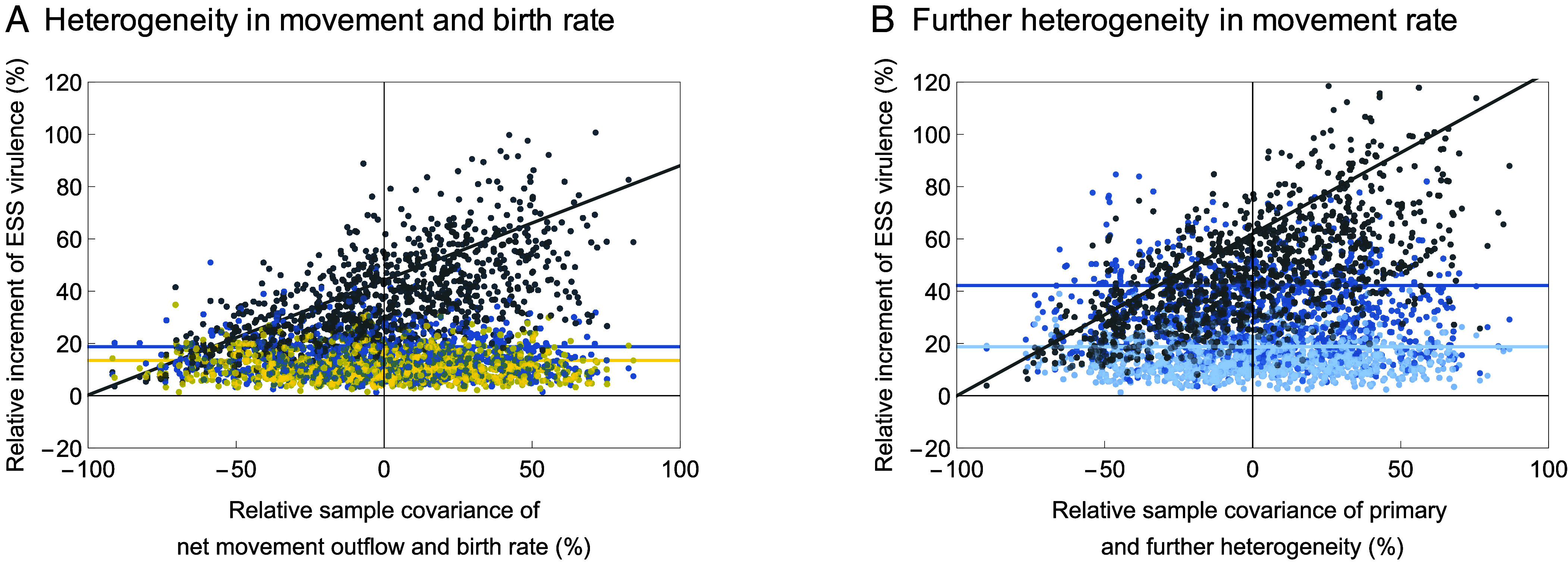
Dependence of the relative increments of ESS virulence caused by two metapopulation heterogeneities on the covariation between these heterogeneities. The two heterogeneities are of different type in (*A*) (here, the heterogeneities in movement rates and birth rates), while they are of the same type in (*B*) (here, the primary and additional heterogeneities in movement rates). Panels (*A*) and (*B*) show on the vertical axes the relative increments of ESS virulence (gray dots) obtained through numerical evolution in each of 1,000 randomly generated metapopulations (created as described in the caption of Fig. 2) in comparison with the ESS virulence predicted by our analytical theory (gray lines) according to Eq. **4a** for (*A*) and Eq. **4c** for (*B*). For comparison, panel (*A*) also shows the relative increments of ESS virulence when the heterogeneity in movement rates is applied alone (blue dots, with blue line for the analytical prediction) and when the heterogeneity in birth rates is applied alone (yellow dots, with yellow line for the analytical prediction). Similarly, panel (*B*) also shows the relative increments of ESS virulence when the primary heterogeneity in movement rates of degree 15% is applied alone (blue dots, with blue line for the analytical prediction) and when the additional heterogeneity in movement rates of degree 10% is applied alone (light-blue dots, with light-blue line for the analytical prediction). See the captions of *SI Appendix*, Figs. S2 and S3 for further details.

[4a] $$\frac{\alpha^{*}-\alpha_{0}^{*}}{\alpha_{0}^{*}}=\Theta \left(q_{x}^{2}{\hat{V}}_{x}+2q_{x}q_{y}{\hat{C}}_{\mathrm{xy}}+q_{y}^{2}{\hat{V}}_{y}\right),$$

where ${\hat{V}}_{x}=\mathrm{Var}\left(x\right)/x_{0}^{2}$ and ${\hat{V}}_{y}=\mathrm{Var}\left(y\right)/y_{0}^{2}$ are the relative variances of x and y, and ${\hat{C}}_{\mathrm{xy}}=Cov\left(x,y\right)/\left(x_{0}y_{0}\right)$ is the relative covariance of x and y (*SI Appendix*, section S2.6). Thus, when two heterogeneities are uncorrelated, their joint effect is simply the sum of each heterogeneity’s individual effect. A positive correlation between two heterogeneities boosts the increase in ESS virulence (Fig. 5*A*, and *SI Appendix*, Fig. S2). When two heterogeneities are strongly positively correlated, a mere 10% of variation in each heterogeneous parameter suffices to cause as much as an 80% increase in ESS virulence (Fig. 5*A* and *SI Appendix*, Fig. S2). While a negative correlation between two heterogeneities diminishes the increase in ESS virulence, the net effect on ESS virulence is always positive even for two strongly negatively correlated heterogeneities, as proved by $q_{x}^{2}{\hat{V}}_{x}+2q_{x}q_{y}{\hat{C}}_{\mathrm{xy}}+q_{y}^{2}{\hat{V}}_{y}=\mathrm{Var}\left(q_{x}x/x_{0}+q_{y}y/y_{0}\right)\geq 0$.

The extension of Eq. **6b** to three or more heterogeneities is straightforward,

[4b] $$\frac{\alpha^{*}-\alpha_{0}^{*}}{\alpha_{0}^{*}}=\Theta \left(\sum_{k}q_{x_{k}}^{2}{\hat{V}}_{x_{k}}+\sum_{k}\sum_{l\neq k}q_{x_{k}}q_{x_{l}}{\hat{C}}_{x_{k}x_{l}}\right),$$

where $x_{k}$ and $x_{l}$ denote the k th and l th heterogeneous parameters.

To sum up, when several of the discussed heterogeneities occur together, their effect on the ESS virulence is just the sum of the effects of each heterogeneity alone when they are uncorrelated, more than this sum when they are positively correlated, and still positive even when they are strongly negatively correlated.

### Additional Heterogeneities in Already Heterogeneous Metapopulations Increase Virulence According to the Sum of Their Variances and Covariances.

In our analysis so far, we have assumed that heterogeneities are introduced in a homogeneous metapopulation. We now relax this assumption by considering the effect of additional heterogeneity on ESS virulence in an already heterogeneous metapopulation. The analysis and the results are very similar to the case of multiple heterogeneities discussed above, with the relative increment of ESS virulence given by

[4c] $$\frac{\alpha^{*}-\alpha_{0}^{*}}{\alpha_{0}^{*}}=\Theta q_{x}^{2}\left({\hat{V}}_{x}+2{\hat{C}}_{x\tilde{x}}+{\hat{V}}_{\tilde{x}}\right),$$

where x denotes the primary heterogeneity $x_{i}=x_{0}+\epsilon x_{i}^{\prime }$ and $\tilde{x}$ denotes the additional heterogeneity $x_{i}=x_{0}+\epsilon x_{i}^{\prime }+\epsilon {\tilde{x}}_{i}^{\prime }$. Therefore, if the additional variation is not correlated with the primary variation or if the two variations are positively correlated, the additional heterogeneity further increases ESS virulence from the value $\alpha_{0}^{*}+\Theta q_{x}^{2}{\hat{V}}_{x}$ already increased by the primary heterogeneity (Fig. 5*B* and *SI Appendix*, Fig. S3). If an additional heterogeneity occurs that counteracts or reverses the primary heterogeneity (i.e., the two variations are negatively correlated), the additional heterogeneity may reduce some of the increase in ESS virulence caused by the primary heterogeneity, but, unless the two heterogeneities precisely cancel each other, their net effect on ESS virulence will always be positive, as shown in the previous section.

### Higher-Order Effects Explain Nonlinearities in Virulence Increases Occurring At High Degrees of Heterogeneity.

Our analyses of the effects of metapopulation heterogeneities on ESS virulence so far have concentrated on heterogeneities whose degree is relatively small, leading to a linear dependence of ESS virulence on the variances and covariances of the heterogeneous parameters, as shown in Eqs. **3b** and **4**. It is thus interesting to observe in Fig. 4 that sufficiently strong heterogeneities can cause a slightly nonlinear dependence of ESS virulence on the variance of the heterogeneous parameter.

**Fig. 6.**
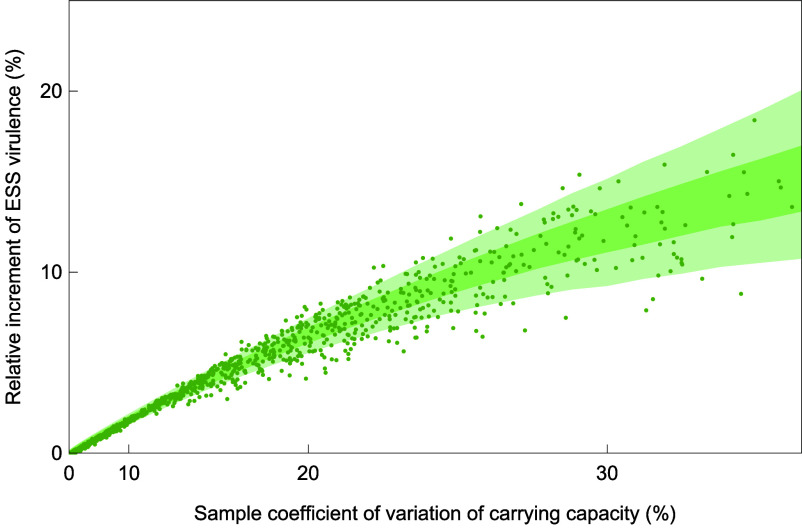
Dependence of the relative increments of ESS virulence caused by metapopulation heterogeneity on the degree of heterogeneity when this degree is large. Our extended analytical theory explains and predicts both the nonlinear increase and the stochastic spread in terms of higher-order effects of heterogeneity. The figure compares numerical (dots) and analytical results (dark-green and light-green areas indicating the predicted 25% to 75% and 5% to 95% percentiles, calculated from the analytically predicted distributions of ESS virulence obtained by substituting higher-order central moments realized in a randomly generated heterogeneous metapopulation into Eqs. **S5.65** to **S5.68** for heterogeneity in carrying capacities based on a simple SIS model in which a deceased host is immediately locally replaced by a newborn host, implying local host populations of constant size (*SI Appendix*, section S5). The horizontal axis is scaled quadratically as in Fig. 3. The target coefficient of variation, $\sigma_{K}/\bar{K}=\epsilon$, equals 20%. All parameter values are as shown in Table 1 except for $\bar{K}=1$, $\eta_{0}=0.15$, a=2.0, and $m_{0}=0.2$.

We find that such nonlinearity can be explained by an additional dependence of ESS virulence on the third and fourth central moments of the heterogeneous parameter (*SI Appendix*, section S5). By taking into consideration the effects of such higher moments, our analytical theory can predict nonlinear increases of ESS virulence for large degrees of heterogeneity (Fig. 6). In this context, it should be noted that even when local parameters are drawn from a symmetric probability distribution like the normal distribution, the sampled third central moment might not be negligible, especially when the number of local populations is small, making small contributions to ESS virulence.

## Results Are Robust for Other Epidemiological Models

Our results described above – based on SIRS models such as in Eq. **5** – robustly hold for other types of epidemiological models, such as SI models, SIS models, and SIR models (*SI Appendix*, section S3). This demonstrates considerable generality.

**Fig. 7.**
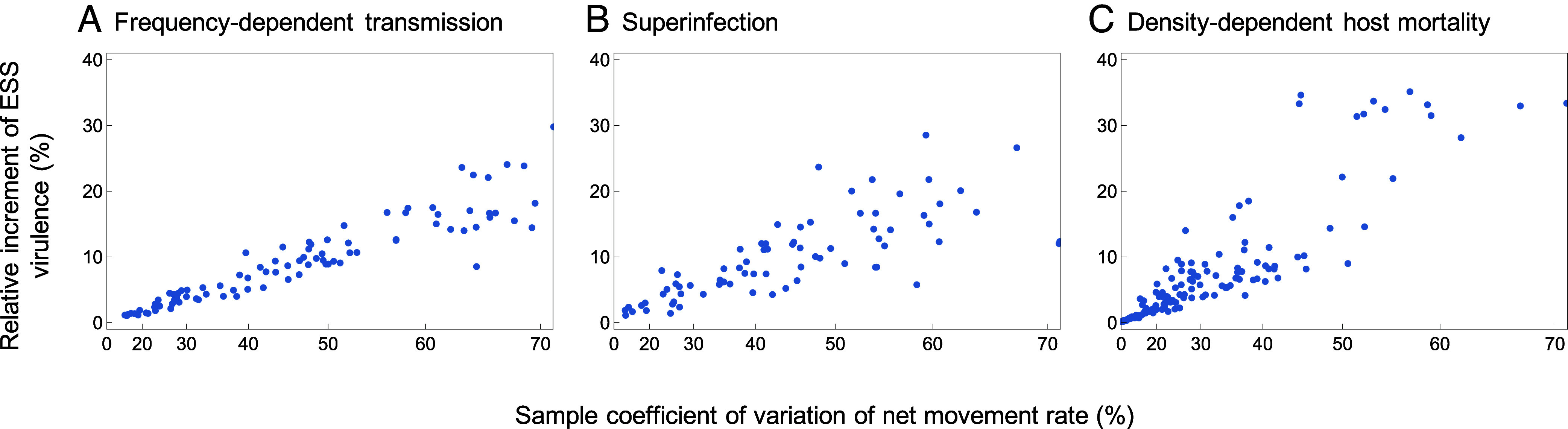
Metapopulation heterogeneity robustly increases ESS virulence in various alternative evo-epidemiological scenarios. (*A*–*C*) Relative increments of ESS virulence caused by metapopulation heterogeneity in movement rate for (*A*) frequency-dependent transmission, (*B*) superinfection, and (*C*) density-dependent mortality causing evolutionary branching in virulence. See *SI Appendix*, section S6 for further details.

Moreover, our main result, that higher degrees of the considered metapopulation heterogeneities always lead to increased ESS virulence, can further be extended to the following three cases (Fig. 7). First, the same set of results shown above, detailed in Eqs. **2** and **3**, follows if we replace density-dependent transmission as assumed in Eq. **5** with frequency-dependent transmission often assumed for sexually transmitted and vector-borne diseases. This robustness is illustrated in Fig. 7*A*. Second, also with superinfection, which is known to increase the ESS virulence already in homogeneous populations (37, 38), metapopulation heterogeneity further increases the ESS virulence. This robustness is illustrated in Fig. 7*B*. Third, even in epidemiological models with more than one environmental-feedback dimension, which break the $R_{0}$ -maximization principle and allow evolutionary branching in pathogen traits (9), e.g., with density-dependent host mortality (39), metapopulation heterogeneity still increases the mean virulence of a dimorphic pathogen population. This robustness is illustrated in Fig. 7*C* and *SI Appendix*, Fig. S5. See *SI Appendix*, section S6 for the detailed specifications of these extensions.

## Discussion

We have studied how metapopulation heterogeneities in movement, birth, carrying capacity, and immunity loss affect the evolution of pathogen virulence and found that such heterogeneities always increase the ESS virulence. Our analytical results also reveal that such increases equal the covariance between local selection pressures and local reproductive values across the metapopulation. The reason why heterogeneities in movement, birth, carrying capacity, and immunity loss always increase the ESS virulence is the positive proportionality of the deviations of local selection pressures and local reproductive values from their metapopulation averages. This means that all local populations in which the considered metapopulation heterogeneity increases the local equilibrium density of susceptible hosts exhibit not only local selection pressures favoring a higher virulence but also local reproductive values that are higher. Since a local reproductive value measures a local population’s relative evolutionary contribution to the metapopulation selection pressure, this means that local selection pressures favoring a lower virulence are contributing less than local selection pressures favoring a higher virulence.

Our results reveal an always-upward effect on pathogen virulence of metapopulation heterogeneities in movement, birth, carrying capacity, and immunity loss, all of which are ubiquitous in nature. A well-documented example out of many is the heterogeneous movement network among local populations of yeast metapopulations in New Zealand (22). Moreover, when, in the context of conservation ecology, fragmented habitats are connected through corridors to facilitate movement between the habitats and counteract habitat loss (40), this inevitably results in heterogeneous movement networks. The striking ubiquity of movement heterogeneity across ecosystems suggests that the classical theory predicting virulence evolution assuming homogenous host populations could seriously and systematically underestimate the severity of evolutionary increases in pathogen virulence. Complementing movement heterogeneity, heterogeneity in habitat quality is no less ubiquitous in metapopulations across ecosystems. This latter type of heterogeneity means that birth rates, carrying capacities, or both differ among local populations. One example is the spatial heterogeneity in the carrying capacities of local populations of deer metapopulations in Japan (20). And while the rate of immunity loss might not be the most likely parameter to be affected by spatial heterogeneity, it may well vary among local populations. For example, it has been demonstrated that the rates at which vaccine-induced immunity to diphtheria is lost vary among European countries due to differences in the number of booster shots subjects receive, the scheduling of these booster shots, and the age groups to which they are offered (41). Together, the four analyzed types of heterogeneity are virtually certain to drive up ESS virulences in natural metapopulations.

Our theory reveals the quantitative dependence of ESS virulences on the type, degree, and covariation of metapopulation heterogeneity and thus can tell us how large an increase in ESS virulences must be expected for any given type, degree, and covariation of metapopulation heterogeneities. Detailed demographic and epidemiological data have become available in recent years with which this quantitative understanding can be used for deriving detailed empirically testable predictions.

When conducting specific practical interventions affecting heterogeneous metapopulations, it is necessary to consider whether they change not only the variance of local conditions but also the mean of local conditions. Unlike the effects of changes in the variance of local conditions on ESS virulence – for which we have found a predictable, general, analytical, qualitative, always-upward dependence –, the effects of changes in the mean of local conditions on ESS virulence could be more complex. As an illustrative example, we numerically analyze the impact of the total movement rate $m_{0}$ on the ESS virulence. As shown in *SI Appendix*, Fig. S6, the ESS virulence is maximized at intermediate $m_{0}$ and shrinks as $m_{0}$ approaches 0 or infinity, at either extreme approaching the ESS of a homogeneous metapopulation. This is not surprising at all: At these extremes, the metapopulation respectively comprises fully isolated or fully connected local populations, whose ESS virulences accordingly approach the ESS of a homogeneous metapopulation. While finding qualitative patterns in the effects of changes in the mean is challenging, *SI Appendix*, Fig. S6 demonstrates how our theory allows for the quantitative evaluation of the effects of practical interventions affecting both the variance and the mean of local conditions and can therefore help predict the impacts of both.

In our model, the basic reproduction number $R_{0}$ of pathogens is maximized by their evolution, just as in the corresponding classical models (1, 7, 42). There are, however, a variety of interesting circumstances under which the $R_{0}$ -maximization principle no longer holds. For example, under spatial viscosity (43), superinfection (38), or density-dependent host mortality (39), there exists no quantity such as $R_{0}$ that is maximized by evolution. This happens in a variety of models in which the environmental-feedback dimension is greater than 1 (9, 44). There is no obvious reason to believe that higher environmental-feedback have a general tendency to promote or diminish the virulence-increasing effect of metapopulation heterogeneity highlighted in this study. Indeed, even for heterogeneous metapopulation models with superinfection or density-dependent mortality, in which $R_{0}$ is no longer maximized by evolution, we have shown that higher degrees of metapopulation heterogeneities increase the evolved virulence (Fig. 7).

We have focused on environmental heterogeneity among local populations that themselves are large and well-mixed enough to be sufficiently free from demographic stochasticity and spatial self-structuring. It is interesting to compare the effects of environmental heterogeneity among local populations and spatial viscosity within local populations resulting from the spatially limited dispersal and interaction of individuals (12, 43, 45). Specifically, spatial viscosity due to spatially limited dispersal, infection, and birth enable spatial self-structuring (i.e., generate self-organized spatial correlations between neighboring individuals). This changes the biotic environment experienced by individuals in their immediate surroundings from place to place within local populations. Spatial viscosity within local populations is known to favor milder virulence and infectiousness in spatially explicit SI models (12, 43) and more severe virulence and infectiousness in spatially explicit SIR models (45). This internally induced spatial heterogeneity needs to be clearly distinguished from the externally induced spatial heterogeneity we have focused on here. An interesting extension of our study will therefore be to ask what happens to the evolution of virulence when these two different types of spatial heterogeneities are combined.

We have achieved our analytical evaluations of the effects of metapopulation heterogeneities on ESS virulence by perturbation analyses with respect to the considered metapopulation heterogeneities. In previous studies, similar expressions have been derived for class-structured populations by summing class-specific selection pressures weighted by class-specific reproductive values (30–32). Indeed, recent studies have shown that such reproductive-value-weighted expression can generally be derived under the assumption of weak selection (10, 11, 33). In our analyses, we have stepped beyond this previously existing framework by combining reproductive-value-weighted sums of selection pressures with their expansions with respect to small degrees of metapopulation heterogeneities (35) to obtain general results characterizing the evolution of pathogens in heterogeneous metapopulations. It may thus be worth highlighting that our explicit results demonstrating the always-upward effects of heterogeneities in movement, birth, carrying capacity, and immunity loss on ESS virulence can only be revealed by applying a Taylor expansion for small degrees of heterogeneity to the classical reproductive-value-weighted form of invasion fitness in structured populations.

While our results have important implications for diseases in heterogeneous host populations, a particularly relevant application is the management of fragmented habitats – a key challenge in conservation ecology. Indeed, habitat fragmentation is considered to be one of the most serious threats to biodiversity, and related conservation measures will become increasingly important in future ecosystem management (46). A prominent strategy for countering habitat fragmentation is to link fragmented habitats by building corridors between them, which has been demonstrated to benefit conservation efforts (40). On the other hand, the risk of facilitating the spreading of infectious diseases by connecting fragmented habitats has been raised as a concern (47, 48). Our results contribute to the debate over such conservation tradeoffs by revealing a threat: Whenever habitats or corridors are heterogeneous, connecting habitats will select for more virulent and more infectious pathogens. While it is important to evaluate these effects of increased movement heterogeneity together with the effects of increased total movement rate (*SI Appendix*, Fig. S6), as both are caused by corridors, our findings highlight the potential for raised pathogen virulence and infectivity under these conditions.

Our results hold for all host-mediated metapopulation heterogeneities, yet this classification of heterogeneities also highlights a limitation to the applicability of our results concerning the always-upward effect of these heterogeneities on ESS virulence. This is because there are local conditions and associated parameters in our ecoepidemiological model in Eq. **5** that have direct impacts on local selection pressures and local reproductive values that do not operate only through the local densities of susceptible hosts. Examples are the rates of host natural mortality and recovery as well as the rate of pathogen transmission. Accordingly, we refer to all such heterogeneities as having a direct effect. For these direct-effect heterogeneities, we cannot presume the positive proportionality that guarantees the always-upward effect of heterogeneities on ESS virulence, instead requiring technically even more involved investigations. Our detailed analysis of direct-effect heterogeneities is underway. This is why we explicitly refer to heterogeneities in movement, birth, carrying capacity, and immunity loss for stating our main results and why we emphasize how ubiquitous and important such heterogeneities are in nature.

Here we have unveiled a simple and general principle for the effect of host-mediated heterogeneities on the evolution of pathogen virulence. Our results are robust for many epidemiological models – including SI, SIS, SIR, and SIRS models, with both density- and frequency-dependent transmission as well as with superinfection – and hence are applicable to many infectious diseases in metapopulations (*SI Appendix*, section S3 and S6). Extending this theory to apply to the evolution of plants or animals living in metapopulations is an interesting future direction. We close this study with a brief and simple reflection applying our results to the mounting concentration and connectivity of human populations. Nowadays, one out of eight people live in just 33 megacities with more than 10 million inhabitants (49). Such conditions imply high degrees of heterogeneity in local conditions, and we therefore suggest that they not only elevate risks of epidemics or pandemics by facilitating pathogen spread but also elevate risks of the emergence of highly virulent and infectious pathogens as predicted by our results. What is more, human populations are increasingly connected by powerful transportation networks, enabling movements over unprecedented distances and at unprecedented intensities. International migration is increasing, and their origins and destinations are highly heterogeneous: The total number of international migrants has increased by 80% from 1990 to 2020 (50), and the coefficient of variation of the estimated net migration rates for the period 2005-2010 equals about 300% (51). Such conditions imply high degrees of heterogeneity in human movement networks. While we must separately consider the effects of increased total movement, such changes in human movement not only elevate risks of epidemics or pandemics by facilitating the spread of diseases afflicting human populations but also elevate risks of the emergence of highly virulent and infectious pathogens, as our results predict.

## Model and Methods

### Metapopulation Epidemiology.

To study the impact of metapopulation heterogeneity on the evolution of pathogen virulence and infectiousness, we consider a general SIRS model with susceptible (S), infected (I), and recovered-and-immune (R) hosts in n local populations connected by host movement (Fig. 1),

[5a] $$\begin{array}{c} \frac{dS_{i}}{dt}=r_{i}\left(1-\frac{N_{i}}{K_{i}}\right)N_{i}-\mu S_{i}+\eta I_{i}+\nu_{i}R_{i}-\beta S_{i}I_{i}\\ \;+\sum_{j=1}^{n}\left(m_{\mathrm{ij}}S_{j}-m_{\mathrm{ji}}S_{i}\right), \end{array}$$

[5b] $$\frac{dI_{i}}{dt}=\beta S_{i}I_{i}-\left(\mu +\alpha +\eta +\gamma \right)I_{i}+\sum_{j=1}^{n}\left(m_{\mathrm{ij}}I_{j}-m_{\mathrm{ji}}I_{i}\right),$$

[5c] $$\frac{dR_{i}}{dt}=\gamma I_{i}-\left(\mu +\nu_{i}\right)R_{i}+\sum_{j=1}^{n}\left(m_{\mathrm{ij}}R_{j}-m_{\mathrm{ji}}R_{i}\right),$$

where the densities of susceptible, infected, and recovered-and-immune hosts in local population i=1,⋯,n are denoted by $S_{i},I_{i}$, and $R_{i}$, respectively, and $N_{i}=S_{i}+I_{i}+R_{i}$ is the total host density in local population i. All model variables and parameters are described in Table 1. The rate at which a host in local population i moves to local population j is $m_{\mathrm{ji}}$. Our model allows back movement, i.e., the movement of a host from local population i to i.

An alternative class of models covered by our analyses are those involving frequency-dependent transmission, which are often used to describe the dynamics of sexually transmitted diseases and vector-borne diseases. In such models, the changes $\beta S_{i}I_{i}$ by infection in Eqs. **4a** and **4b** are replaced with $\beta S_{i}I_{i}/N_{i}$. We demonstrate that all results shown in this study carry over to such alternative models (*SI Appendix*, section S6).

### Virulence Evolution in Homogeneous Metapopulations.

Classical theory of virulence evolution in homogeneous populations predicts that the endpoint of virulence evolution, i.e., the corresponding ESS of the pathogen, maximizes the basic reproduction number $R_{0}=\beta S_{0}/\left(\mu +\alpha +\eta +\gamma \right)$, where $S_{0}=K_{0}\left(1-\mu /r_{0}\right)$ is the equilibrium density of susceptible hosts in the disease-free population. The basic reproduction number $R_{0}$ describes the expected number of secondary infections resulting from a single primarily infected host in an initially disease-free host population: After the infection of the primarily infected host in the disease-free population, the pathogen secondarily infects susceptible hosts at the rate $\beta S_{0}$ during the primarily infected host’s lifetime 1/(μ+α+η+γ), which explains the expression for $R_{0}$.

Following standard practice (8, 42, 52), we assume a tradeoff between the pathogen’s transmission rate β and the pathogen’s virulence α so that β monotonically increases with α (i.e., dβ/dα> >0) with a diminishing return (i.e., $d^{2}\beta /d\alpha^{2}<0$). If pathogen virulence is too high, pathogens spread with a high transmission rate but kill their hosts quickly and therefore have less-than-optimal chances to cause secondary infections. Conversely, if pathogen virulence is too low, pathogens keep their host alive for longer but spread with a low transmission rate and therefore again have less-than-optimal chances to cause secondary infections. Thus, there is an intermediate ESS virulence that maximizes the basic reproduction number by balancing the transmission rate β(α) within the period 1/(μ+α+η+γ) during which transmission can occur.

The principle of $R_{0}$ maximization applies also to the metapopulations described by our model. To see this for homogeneous metapopulations, we examine whether a rare variant pathogen with virulence $\hat{\alpha }$ can spread in a host metapopulation infected by a resident pathogen with virulence α. The per capita growth rate, or invasion fitness, of the rare variant pathogen is $\hat{\lambda }=\beta \left(\hat{\alpha }\right)S_{0}^{*}-\left(\mu +\hat{\alpha }+\eta +\gamma \right)$, where $S_{0}^{*}=\left(\mu +\alpha +\eta +\gamma \right)/\beta =S_{0}/R_{0}$ is the density of susceptible hosts after the spread of the resident pathogen has equilibrated. If and only if the variant pathogen has a positive per capita growth rate $\hat{\lambda }$, it can spread in the resident population. Substituting the variant pathogen’s basic reproduction number ${\hat{R}}_{0}$, the condition for its invasion success thus is $\hat{\lambda }=\left(\mu +\hat{\alpha }+\eta +\gamma \right)\left({\hat{R}}_{0}/R_{0}-1\right)>0$. Therefore, only variant pathogens can invade that have larger basic reproduction numbers than the resident, which means that, after multiple such invasions, the basic reproduction number is maximized by pathogen evolution. Accordingly, the ESS virulence $\alpha_{0}^{*}$ satisfies $s_{0}\left(\alpha_{0}^{*}\right)=0$

[6] $$s_{0}\left(\alpha \right)={\left.\frac{\partial \hat{\lambda }}{\partial \hat{\alpha }}\right|}_{\hat{\alpha }=\alpha }=\frac{\mu +\alpha +\eta +\gamma }{R_{0}}{\left.\frac{\partial {\hat{R}}_{0}}{\partial \hat{\alpha }}\right|}_{\hat{\alpha }=\alpha },$$

where is the selection pressure on pathogen virulence α in a homogeneous metapopulation, defined as the derivative of the variant pathogen’s invasion fitness with respect to the variant pathogen’s virulence $\hat{\alpha }$ evaluated at the resident pathogen’s virulence, $\hat{\alpha }=\alpha$.

For illustration, we use the standard tradeoff function $\beta \left(\alpha \right)=$ $a\sqrt{\alpha }$ throughout this study. Under this tradeoff, the ESS virulence in a homogeneous metapopulation equals $\alpha_{0}^{*}=\mu +\eta +\gamma$.

### Metapopulation Heterogeneities.

The demographic and epidemiological parameters affecting the birth and immunity loss of hosts may vary among local populations, with $r_{i}$ denoting the birth rate, $K_{i}$ the carrying capacity, and $\nu_{i}$ the immunity-loss rate in population i (Fig. 1). Also the movement of hosts between local populations may vary among pairs of local populations, with $m_{\mathrm{ji}}$ denoting the rate at which a host in local population i moves to local population j (Fig. 1). The other parameters affecting the dynamics of infected hosts are assumed to be the same among local populations, including the intrinsic mortality rate u, the recovery rate η to susceptible status, the recovery rate γ to immune status, and the transmission rate β (we consider relaxations of this simplifying assumption in the Discussion section, where we also motivate and explain, based on our analytical results, the range of metapopulation heterogeneities examined in this study).

We quantify the heterogeneities in demographic and epidemiological parameters in terms of the differences between local parameter values and the corresponding metapopulations averages,

[7] $$x_{i}=x_{0}+\epsilon x_{i}^{\prime }\mathrm{and}m_{\mathrm{ij}}=m_{0}/n+\epsilon m_{\mathrm{ij}}^{\prime },$$

where x=r, K, or ν denotes any of the demographic or epidemiological parameters that may vary across the metapopulation, ϵ is a small positive constant that measures the degree of metapopulation heterogeneity, $x_{0}$ and $m_{0}/n$ are the metapopulation averages $x_{0}=\sum_{i}x_{i}/n$ and $m_{0}/n=\sum_{i,j}m_{\mathrm{ij}}/n$, and $x_{i}^{\prime }$ and $m_{\mathrm{ij}}^{\prime }$ determine the deviations of the local parameter values $x_{i}$ and $m_{\mathrm{ij}}$ from these metapopulation averages. By definition, the deviations average to 0, $E\left(x_{i}^{\prime }\right)=\sum_{i}x_{i}^{\prime }/n=0$ and $E\left(m_{\mathrm{ij}}^{\prime }\right)=\sum_{i,j}m_{\mathrm{ij}}^{\prime }/n=0$. To fix the scale of the deviations of different parameters, we could set their coefficients of variation to 1, which would imply that the coefficients of variation of the local parameter values $x_{i}$ and $m_{\mathrm{ij}}$ equal ϵ.

To investigate the effects of metapopulation heterogeneity on pathogen evolution, we randomly and independently draw local parameter values from a multivariate normal probability density function. The sampling variances and covariances of the resultant sets of local parameter values characterize the realized metapopulation heterogeneity and are shown to be the key drivers of the evolution of pathogen virulence and infectiousness in heterogeneous metapopulations.

### Virulence Evolution in Heterogeneous Metapopulations.

As explained above for homogeneous metapopulations, we study the evolution of pathogen virulence and infectiousness in heterogeneous metapopulations by examining the invasion potential of a variant pathogen that differs in its virulence from that of the resident pathogen (Fig. 1). In this way, we identify the endpoints of pathogen evolution.

*SI Appendix*, section S1 provides a summary of our analytical theory. We outline as follows the logical flow of steps needed for deriving our main analytical results in Eqs. 2–3. We start from expressing the metapopulation selection pressure s(α) on pathogen virulence α as


[8a] $$s\left(\alpha \right)=\sum_{i=1}^{n}v_{i}\phi_{i}s_{i}\left(\alpha \right),$$

where $s_{i}\left(\alpha \right)$ is the local selection pressure on pathogen virulence in population i, and $v_{i}$ and $\phi_{i}$, respectively, are the local reproductive value and the local equilibrium proportion of a rare neutral variant pathogen in population i. This formula, which is based on the Rayleigh quotient, is derived under the assumptions applicable for our model framework as shown in the beginning of *SI Appendix*, section S2, and is also known as the Price equation for evolution in structured populations (10, 30, 33), where $v_{i}\phi_{i}$ is called the class reproductive value. Our main results in Eqs. 2 and 3 are obtained by expanding the relative local abundances, local reproductive values, and local selection pressures into Taylor series for a small degree ϵ of metapopulation heterogeneity as introduced in Eq. **7**, i.e., $\phi_{i}=\phi_{0}+\epsilon \phi_{i}^{\prime }+\epsilon^{2}\phi_{i}^{\prime \prime }+O\left(\epsilon^{3}\right)$, $v_{i}=v_{0}+\epsilon v_{i}^{\prime }+\epsilon^{2}v_{i}^{\prime \prime }+O\left(\epsilon^{3}\right)$, and $s_{i}=s_{0}+\epsilon s_{i}^{\prime }+\epsilon^{2}s_{i}^{\prime \prime }+O\left(\epsilon^{3}\right)$, and substituting these expressions into Eq. **8a** to obtain

[8b] $$s\left(\alpha \right)=s_{0}\left(\alpha \right)+\epsilon^{2}\frac{1}{n}\sum_{i=1}^{n}v_{i}^{\prime }s_{i}^{\prime }+O\left(\epsilon^{3}\right),$$

where $s_{0}\left(\alpha \right)$ is the selection pressure in the corresponding homogeneous metapopulation introduced in Eq. **6**, and $\epsilon v_{i}^{\prime }$ and $\epsilon s_{i}^{\prime }$ are the first-order deviations of the local reproductive value and local selection pressure in population i. For deriving Eq. **8b**, we use $\sum_{i}s_{i}^{\prime }=\sum_{i}\phi_{i}^{\prime }=\sum_{i}v_{i}^{\prime }=0$, $\phi_{0}=1/n$, and $\sum_{i}s_{i}^{\prime \prime }/n+\sum_{i}s_{i}^{\prime }\phi_{i}^{\prime }=0$ (*SI Appendix*, section S2). We show in *SI Appendix*, section S2 that both $v_{i}^{\prime }$ and $s_{i}^{\prime }$ are proportional to the first-order deviation $\epsilon {S_{i}^{*}}^{\prime }$ of the local equilibrium density of susceptible hosts in population i, defined by $S_{i}^{*}=S_{0}^{*}+\epsilon S_{i}^{{*}^{\prime }}+O\left(\epsilon^{2}\right)$,

[8c] $$\epsilon v_{i}^{\prime }=\epsilon \frac{\beta }{m_{0}}{S_{i}^{*}}^{\prime },\epsilon s_{i}^{\prime }=\epsilon \frac{d\beta }{d\alpha }{S_{i}^{*}}^{\prime }.$$

Substituting Eq. **8c** into **8b**, we obtain the selection pressure in heterogeneous metapopulation as

[8d] $$s\left(\alpha \right)=s_{0}\left(\alpha \right)+\epsilon^{2}\frac{1}{n}\sum_{i=1}^{n}\frac{\beta }{m_{0}}\frac{d\beta }{d\alpha }{\left({S_{i}^{*}}^{\prime }\right)}^{2}.$$

The increment $\delta s\left(\alpha \right)=s\left(\alpha \right)-s_{0}\left(\alpha \right)$ of the selection pressure in the heterogeneous metapopulation relative to that in the corresponding homogeneous metapopulation is proportional to the variance of local equilibrium densities of susceptible hosts, which is always positive if infectiousness is an increasing function of virulence (dβ/dα> >0). This means that metapopulation heterogeneities in the local equilibrium densities of susceptible hosts generated by metapopulation heterogeneities in mobility, productivity, and immunocompetency always favor increased virulence.

The ESS virulence $\alpha^{*}$ in a heterogeneous metapopulation is defined by $s\left(\alpha^{*}\right)=0$. We expand this as $\alpha^{*}=\alpha_{0}^{*}+\delta \alpha^{*}=\alpha_{0}^{*}+$$\epsilon \alpha_{1}^{*}+\epsilon^{2}\alpha_{2}^{*}+O\left(\epsilon^{3}\right)$, where $\alpha_{0}^{*}$ is the ESS virulence in the corresponding homogeneous metapopulation, which satisfies $s_{0}\left(\alpha_{0}^{*}\right)=0$. Substituting this into Eq. **7d**, we see that the relative increment of ESS virulence in a heterogeneous metapopulation from that in the corresponding homogeneous metapopulation is

[9a] $$\frac{\delta \alpha^{*}}{\alpha_{0}^{*}}=\Theta \frac{1}{n}\sum_{i=1}^{n}{\left(\frac{{S_{i}^{*}}^{\prime }}{S_{0}^{*}}\right)}^{2},$$

where $\Theta ={\left.{\left(-\frac{d^{2}\beta }{d\alpha^{2}}\frac{\alpha^{2}}{\beta }\right)}^{-1}\frac{\alpha }{m_{0}}\right|}_{\alpha =\alpha_{0}^{*}}$ (*SI Appendix*, section S2). *SI Appendix*, section S3 further shows that the first-order deviations of the local equilibrium densities of susceptible hosts are proportional to the first-order deviations of local conditions (mobility, productivity, or immunocompetency),

[9b] $$\frac{{S_{i}^{*}}^{\prime }}{S_{0}^{*}}=q_{x}\frac{x_{i}^{\prime }}{x_{0}},$$

which finally leads to our evolutionary elasticity result in Eq. **3b** for the increment of ESS virulence in a heterogeneous metapopulation from that in the corresponding homogeneous metapopulation.

Further details are provided in *SI Appendix*, S1 to S6. Unless otherwise stated, the numerical illustrations presented in figures throughout this study are based on the default parameter values shown in Table 1. In contrast, our theory is analytical and therefore independent of specific parameter values.

### Evolutionary Elasticities.

We use so-called elasticities to quantify the evolutionary responses of pathogen virulence to metapopulation heterogeneities. In general, an elasticity measures a focal quantity’s relative change in response to another quantity’s relative change. More specifically, the elasticity E of the impact on a quantity A caused by a small change in a quantity B is defined as the ratio of the relative impact magnitude to the relative cause magnitude, $E=\frac{\delta A}{A}/\frac{\delta B}{B}$, where δA and δB are the changes and A and B are the values prior to the changes. The response of quantity A to quantity B is called inelastic when E<1, unit elastic when E=1, and elastic when E>1 (36).

Being defined in relative terms, elasticities are always dimensionless. Therefore, using elasticities not only facilitates the interpretation of evolutionary changes but also makes it possible to compare the impacts on, and of, quantities with different dimensions, such as the rates of movement, birth, and immunity loss, which have the dimension of a rate, and the carrying capacity, which has the dimension of a density.

## Supplementary Material

Appendix 01 (PDF)

## Data Availability

There are no data underlying this work.
