# Supplementary material for: Metapopulation heterogeneities in host mobility, productivity, and immunocompetency always increase virulence and infectiousness
Source: Proc Natl Acad Sci U S A. 2024 Dec 19;121(52):e2309272121. doi: 10.1073/pnas.2309272121 (PMC11670240; doi:10.1073/pnas.2309272121)
Supplement: Supplementary file 1 — Appendix 01 (PDF) [file pnas.2309272121.sapp.pdf]

Supporting Information for

Metapopulation heterogeneities in movement and host-mediated local conditions always increase pathogen virulence and infectiousness

Masato Sato, Ulf Dieckmann and Akira Sasaki

Masato Sato.
E-mail: sato.m.310kg@gmail.com

This PDF file includes:

Supporting text
Figs. S1 to S6
SI References

Supporting Information Text

Contents

S1 Summary for key analytical findings 2

S2 Virulence evolution in heterogeneous metapopulation 5
S2.1 Virulence evolution in single host populations . . . 5
S2.2 Selection gradient in heterogeneous host metapopulations . . . 6
S2.3 Small-heterogeneity approximation for selection gradient in a metapopulation . . . 9
S2.4 Increment of ESS virulence . . . 12
S2.5 Dependence on local conditions . . . 13
S2.6 Multiple heterogeneities . . . 14
S2.7 Additional heterogeneities . . . 14

S3 Equilibrium densities for small heterogeneities 15
S3.1 Endemic equilibrium . . . 15
S3.2 Linear response to local conditions . . . 15
S3.3 Sum of first-order deviations . . . 16
S3.4 First-order deviations of equilibrium densities . . . 16
S3.5 Elasticity to local conditions . . . 17
S3.6 Elasticities in some limiting cases . . . 17

S4 Distribution for the sampling variation of ESS virulence in heterogeneous metapopulation 20
S4.1 Heterogeneities in birth, carrying capacity, and immunity loss . . . 20
S4.2 Heterogeneities in movement . . . 21

S5 Higher-order predictions in heterogeneous metapopulations 24
S5.1 SIS model with constant local host population densities . . . 24
S5.2 Expansion of equilibrium densities . . . 25
S5.3 Eigenvectors of Jacobian for invasion of neutral pathogen variants . . . 28
S5.4 Expansion of dominant right eigenvector . . . 30
S5.5 Expansion of dominant left eigenvector . . . 31
S5.6 Expansion of selection gradient . . . 34
S5.7 Increment of ESS virulence . . . 37

S6 Robustness of our results for more general circumstances 38
S6.1 Frequency-dependent transmission . . . 38
S6.1.1 Analytical results for frequency-dependent transmission model . . . 38
S6.1.2 Numerical evolution with frequency-dependent transmission . . . 40
S6.2 Numerical evolution in superinfection model . . . 40
S6.3 Numerical evolution with density-dependent host mortality . . . 40

S1. Summary for key analytical findings

Here, we briefly summarize the derivations of two of our key findings, that (1) the increment of metapopulation selection pressure caused by metapopulation heterogeneity can be expressed as the covariance of local reproductive values and local selection pressures and that (2) the relative increment of ESS virulence is proportional to the variance of the heterogeneous local conditions and always positive by virtue of the fourfold quantitative concordance between heterogeneous local conditions, local densities of susceptible

hosts, local selection pressures, and local reproductive values. The compactness of the derivations here is made possible by focusing on their backbones, borrowing non-trivial results in Eqs. (S1.1), (S1.9), and (S1.12) from our full derivations presented in Appendices S2-S3.

Increment of metapopulation selection pressure. For the derivations in this Appendix S1, we consider the metapopulation selection pressure $s(\alpha)$ on pathogen virulence α to be given by the sum of the local selection pressures s_i across all local populations i weighted by the corresponding local class reproductive values $v_i\phi_i$ (i.e., reproductive value of a local pathogen population multiplied by its frequency ϕ_i),

$$s(\alpha) = \sum_{i=1}^{n} v_i s_i \phi_i \qquad [S1.1]$$

(see [S2.15] of SI Appendix S2 for the derivation). In other words, the selection gradient is expressed as the weighted average of local selection gradient s_i, where the weight is class reproductive value, $v_i\phi_i$, of local population i (Taylor 1990; Rousset 1999, 2004; Lion 2018). With metapopulation heterogeneity, the local selection pressures vary among local populations at the same magnitude as the local conditions vary among local populations: hence, $s_i = s_0 + \varepsilon s_i' + \varepsilon^2 s_i''$, where s_0 is the selection pressure in the absence of metapopulation heterogeneity, ε is the degree of metapopulation heterogeneity, and $\varepsilon s_i'$ and $\varepsilon^2 s_i''$ are the first and second-order deviations from s_0 of the local selection pressure in local population i. Similarly, the local reproductive values vary among local populations according to $v_i = v_0 + \varepsilon v_i' + \varepsilon^2 v_i''$, where v_0 is the reproductive value in the absence of metapopulation heterogeneity and $\varepsilon v_i'$ and $\varepsilon^2 v_i''$ are the first and second-order deviation from v_0 of the local reproductive value in local population i. The relative densities among metapopulation of susceptible hosts also vary among local populations according to $\phi_i = \phi_0 + \varepsilon\phi_i'$, where $\phi_0 = 1/n$ is the relative density in the absence of metapopulation heterogeneity and $\varepsilon\phi_i'$ is the first-order deviation from ϕ_0 of the relative densities of susceptible hosts in local population i. Substituting these three expressions into Eq. (S1.1) and expanding the result as a Taylor series with respect to ε gives

$$\begin{aligned} s(\alpha) &= \sum_{i=1}^{n}(v_0 + \varepsilon v_i' + \varepsilon^2 v_i'')(s_0 + \varepsilon s_i' + \varepsilon^2 s_i'')(\phi_0 + \varepsilon\phi_i' + \varepsilon^2\phi_i'') + O(\varepsilon^3) \\ &= s_0 + \varepsilon^2 \frac{1}{n}\sum_{i=1}^{n} v_i' s_i' + O(\varepsilon^3) \qquad [S1.2] \end{aligned}$$

since $v_0 = 1$ and $\phi_0 = 1/n$ according to Eqs. (S2.39)-(S2.40), the averages of the first-order deviations in local selection pressure and reproductive value vanish by construction, $\sum_{i=1}^{n} s_i' = \sum_{i=1}^{n} v_i' = \sum_{i=1}^{n} \phi_i' = 0$, according to Es. (S2.41)-(S2.43), $\sum_{i=1}^{n}(v_i''\phi_0 + v_i'\phi_i' + v_0\phi_i'') = 0$ according to the second-order term of the normalization $\boldsymbol{v}^\top\boldsymbol{\phi} = 1$ (Eq. S2.35), and $(1/n)\sum_i s_i'' + \sum_i s_i'\phi_i' = 0$ that follows from $\boldsymbol{v}_0^\top(\boldsymbol{J}''\boldsymbol{\phi}_0 + \boldsymbol{J}'\boldsymbol{\phi}' + \boldsymbol{J}_0\boldsymbol{\phi}'') = 0$, i.e., by multiplying the zeroth order reproductive value vector to the second order eigenequation for the frequency vector $\boldsymbol{\phi}$ (Eq. S2.26). Hence, up to terms of order of ε^2, we have

$$\begin{aligned} s(\alpha) - s_0 &= \varepsilon^2 \frac{1}{n}\sum_{i=1}^{n} v_i' s_i' \\ &= \frac{1}{n}\sum_{i=1}^{n}(v_i - v_0)(s_i - s_0) \\ &= \mathrm{Cov}(v_i, s_i). \qquad [S1.3] \end{aligned}$$

Therefore, the change in metapopulation selection pressure caused by metapopulation heterogeneity can be expressed as the covariance of local reproductive values and local selection pressures.

Relative increment of ESS virulence. For a degree ε of metapopulation heterogeneity, the ESS virulence α^* in the heterogeneous metapopulation changes from the ESS virulence α_0^* in the corresponding homogeneous metapopulation according to $\alpha^* = \alpha_0^* + \varepsilon\alpha_1^* + \varepsilon^2\alpha_2^* + O(\varepsilon^3)$. Substituting this expression into $s(\alpha^*) = s_0(\alpha^*) + \varepsilon^2 s_2(\alpha^*) + O(\varepsilon^3)$ with the first-order term omitted according to Eq. (S1.2), expanding $s_0(\alpha^*)$ and $s_2(\alpha^*)$ as Taylor series with respect to ε, and using the ESS conditions $s(\alpha^*) = 0$ and $s_0(\alpha_0^*) = 0$ gives

$$\begin{aligned} 0 &= s_0(\alpha^*) + \varepsilon^2 s_2(\alpha^*) + O(\varepsilon^3) \\ &= s_0(\alpha_0^*) + \frac{\mathrm{d}s_0}{\mathrm{d}\alpha}(\alpha_0^*)\left(\varepsilon\alpha_1^* + \varepsilon^2\alpha_2^*\right) + \frac{1}{2}\frac{\mathrm{d}^2 s_0}{\mathrm{d}\alpha^2}(\alpha_0^*)\,\varepsilon^2\alpha_1^{*2} + \varepsilon^2 s_2(\alpha_0^*) + O(\varepsilon^3) \\ &= \varepsilon\frac{\mathrm{d}s_0}{\mathrm{d}\alpha}(\alpha_0^*)\,\alpha_1^* + \varepsilon^2\left(\frac{\mathrm{d}s_0}{\mathrm{d}\alpha}(\alpha_0^*)\,\alpha_2^* + \frac{1}{2}\frac{\mathrm{d}^2 s_0}{\mathrm{d}\alpha^2}(\alpha_0^*)\,\alpha_1^{*2} + s_2(\alpha_0^*)\right) + O(\varepsilon^3). \end{aligned} \qquad [\text{S1.4}]$$

For Eq. (S1.4) to hold for different values of ε, the coefficients of ε and ε^2 must vanish individually: this yields $\alpha_1^* = 0$ and $\alpha_2^* = -s_2(\alpha_0^*)/\frac{\mathrm{d}s_0}{\mathrm{d}\alpha}(\alpha_0^*)$, where

$$s_2(\alpha_0^*) = \frac{1}{n}\sum_{i=1}^{n} v_i' s_i' \qquad [\text{S1.5}]$$

according to Eq. (S1.2) and

$$\frac{\mathrm{d}s_0}{\mathrm{d}\alpha}(\alpha_0^*) = \frac{\mathrm{d}^2\beta}{\mathrm{d}\alpha^2}(\alpha_0^*)S_0^* \qquad [\text{S1.6}]$$

(see S2 for the derivation). Therefore, the increment $\delta\alpha^* = \alpha^* - \alpha_0^* = \varepsilon^2\alpha_2^* + O(\varepsilon^3)$ of ESS virulence is given by

$$\delta\alpha^* = \varepsilon^2\left(-\frac{\mathrm{d}^2\beta}{\mathrm{d}\alpha^2}(\alpha_0^*)S_0^*\right)^{-1}\frac{1}{n}\sum_{i=1}^{n} v_i' s_i' + O(\varepsilon^3). \qquad [\text{S1.7}]$$

Since the magnitudes of the local reproductive values and of the local selection pressures both depend on how much pathogen reproduction is expected in each local population, the first-order deviations $\varepsilon v_i'$ in local reproductive values and $\varepsilon s_i'$ in local selection pressures caused by metapopulation heterogeneity are both proportional to the first-order deviations $\varepsilon S_i^{*\prime}$ in the local density of susceptible hosts,

$$\varepsilon v_i' = \varepsilon\frac{\beta}{m_0}S_i^{*\prime}, \qquad [\text{S1.8}]$$

$$\varepsilon s_i' = \varepsilon\frac{\mathrm{d}\beta}{\mathrm{d}\alpha}(\alpha_0^*)S_i^{*\prime}, \qquad [\text{S1.9}]$$

(see Eqs. [S2.43] and [S2.45] of S2.3 for the derivations). Substituting these proportionalities into Eq. (S1.7) gives

$$\begin{aligned} \frac{\delta\alpha^*}{\alpha_0^*} &= \varepsilon^2\left(-\frac{\mathrm{d}^2\beta}{\mathrm{d}\alpha^2}(\alpha_0^*)S_0^*\right)^{-1}\frac{\mathrm{d}\beta}{\mathrm{d}\alpha}(\alpha_0^*)\frac{\beta}{\alpha_0^* m_0}\frac{1}{n}\sum_{i=1}^{n}\left(S_i^{*\prime}\right)^2 \\ &= \left(-\frac{\mathrm{d}^2\beta}{\mathrm{d}\alpha^2}(\alpha_0^*)\right)^{-1}\left(\frac{\mathrm{d}\beta}{\mathrm{d}\alpha}(\alpha_0^*)S_0^*\right)\frac{\beta}{\alpha_0^* m_0}\mathrm{Var}\left(\frac{S_i^*}{S_0^*}\right) \\ &= \left(-\frac{\mathrm{d}^2\beta}{\mathrm{d}\alpha^2}(\alpha_0^*)\right)^{-1}\frac{\beta}{\alpha_0^* m_0}\mathrm{Var}\left(\frac{S_i^*}{S_0^*}\right) \\ &= \Theta\mathrm{Var}\left(\frac{S_i^*}{S_0^*}\right), \end{aligned} \qquad [\text{S1.10}]$$

where

$$\Theta = \left(-\frac{\mathrm{d}^2\beta}{\mathrm{d}\alpha^2}(\alpha_0^*)\right)^{-1}\frac{\beta}{\alpha_0^* m_0}. \qquad [\text{S1.11}]$$

The first-order deviations $\varepsilon S_i^{*\prime}$ in local equilibrium densities of susceptible hosts caused by metapopulation heterogeneity are proportional to the first-order deviations $\varepsilon x_i'$ of local conditions described by a parameter $x = r, K, \nu, T$,

$$\varepsilon \frac{S_i^{*\prime}}{S_0^*} = \varepsilon q_x \frac{x_i'}{x_0}. \quad \text{[S1.12]}$$

where the local parameter values x_i vary around their mean x_0 according to $x_i = x_0 + \varepsilon x_i'$ (see SI Appendices S3 for the derivation and the explicit expression for the coefficients q_x). Substituting this proportionality into Eq. (S1.10) gives

$$\frac{\delta\alpha^*}{\alpha_0^*} = \Theta \mathrm{Var}\left(q_x \frac{x_i'}{x_0}\right) = \Theta q_x^2 \mathrm{Var}\left(\frac{x_i}{x_0}\right) = \mathscr{E}_x \frac{\mathrm{Var}(x)}{x_0^2}, \quad \text{[S1.13]}$$

where $\mathscr{E}_x = \Theta q_x^2$. Therefore, the relative increment of ESS virulence caused by metapopulation heterogeneity is proportional to the variance of parameter x and hence is always positive.

S2. Virulence evolution in heterogeneous metapopulation

S2.1. Virulence evolution in single host populations. Here we first review the classical results for the evolution of pathogen virulence in a single host population.

We consider a host organism in a single population and focus on the evolution of virulence of a pathogen that causes infectious disease in the host. We denote the densities of susceptible, infected, and recovered and immune hosts by S, I, and R, respectively. These densities change with time as

$$\begin{aligned}\frac{\mathrm{d}S}{\mathrm{d}t} &= r\left(1 - \frac{N}{K}\right)N - \mu S + \eta I + \nu R - \beta S I,\\ \frac{\mathrm{d}I}{\mathrm{d}t} &= \beta S I - (\mu + \alpha + \eta + \gamma) I, \quad \text{[S2.1]}\\ \frac{\mathrm{d}R}{\mathrm{d}t} &= \gamma I - (\mu + \nu) R,\end{aligned}$$

where $N = S + I + R$ is the total host density, r, μ, and K are the birth rate, natural mortality, and carrying capacity of the host, β, γ, η, and ν are the transmission rate, recovery rate, infection-loss rate, and immunity-loss rate of the infectious disease, and α is the pathogen virulence or disease-induced host mortality.

Now we consider a new pathogen variant that has a different virulence $\hat{\alpha}$ and transmission rate $\hat{\beta} = \beta(\hat{\alpha})$ than the resident, where $\beta = \beta(\alpha)$ describes the tradeoff between transmission rate β and virulence α. When the density $\hat{I}$ of hosts infected by the variant is small, it changes with time as

$$\frac{\mathrm{d}\hat{I}}{\mathrm{d}t} = \left[\hat{\beta} S^* - (\mu + \hat{\alpha} + \eta + \gamma)\right]\hat{I}, \quad \text{[S2.2]}$$

where $S^* = (u + \alpha + \eta + \gamma)/\beta$ is the equilibrium density of susceptible hosts in the resident population. The variant can invade the population when its marginal growth rate, $\hat{\beta} S^* - (\mu + \hat{\alpha} + \eta + \gamma)$, is positive, while it cannot invade the population if its marginal growth rate is negative. Hence, the invasion fitness of the variant is given by

$$w(\hat{\alpha}, \alpha) = \beta(\hat{\alpha}) S^* - (\mu + \hat{\alpha} + \eta + \gamma) = \beta(\hat{\alpha}) \frac{\mu + \alpha + \eta + \gamma}{\beta(\alpha)} - (\mu + \hat{\alpha} + \eta + \gamma). \quad \text{[S2.3]}$$

The selection gradient $\mathscr{S}(\alpha)$ and the fitness curvature $\mathscr{D}(\alpha)$ for pathogen virulence are then defined by

$$\mathscr{S}(\alpha) = \left.\frac{\partial w}{\partial \hat{\alpha}}\right|_{\hat{\alpha}=\alpha} = \frac{\mathrm{d}\beta}{\mathrm{d}\alpha}(\hat{\alpha}) S^* - 1, \quad \text{[S2.4]}$$

$$\mathscr{D}(\alpha) = \left.\frac{\partial^2 w}{\partial \hat{\alpha}^2}\right|_{\hat{\alpha}=\alpha} = \frac{\mathrm{d}^2\beta}{\mathrm{d}\alpha^2}(\hat{\alpha}) S^*. \quad \text{[S2.5]}$$

Under the standard assumption for describing the transmission-virulence tradeoff by a monotonically increasing and concave function $\beta(\alpha)$ (i.e., $\mathrm{d}\beta/\mathrm{d}\alpha > 0$ and $\mathrm{d}^2\beta/\mathrm{d}\alpha^2 < 0$), this implies that the evolutionarily stable (or ESS, for short) virulence (defined by $\mathcal{S}(\alpha) = 0$ and $\mathcal{D}(\alpha) < 0$) satisfies

$$\mathcal{S}(\alpha) = \frac{\mathrm{d}\beta}{\mathrm{d}\alpha}(\alpha)\frac{u + \alpha + \eta + \gamma}{\beta(\alpha)} - 1 = (\mu + \alpha + \eta + \gamma)\frac{1}{\mathcal{R}_0}\frac{\mathrm{d}\mathcal{R}_0}{\mathrm{d}\alpha} = 0, \qquad \text{[S2.6]}$$

where $\mathcal{R}_0 = \beta S_0/(\mu+\alpha+\eta+\gamma)$, with $S_0 = K(1-\mu/r)$ being the equilibrium host density in the disease-free population, is the basic reproduction ratio of the pathogen. Eq. (S2.6) implies the well-known result that the virulence evolution in a single host population leads to the maximization of the basic reproduction ratio $\mathcal{R}_0$ of the pathogen.

S2.2. Selection gradient in heterogeneous host metapopulations.

Epidemiological dynamics We now extend the epidemiological dynamics, Eq. (S2.1), to cases of hosts living in a heterogeneous metapopulation in which n local populations $i = 1, \ldots, n$ with different birth rates r_i, carrying capacities K_i, and immunity-loss rates ν_i are connected by host movements with rates $\widetilde{m}_{ij}/n$ from population j to population i:

$$\begin{aligned}
\frac{\mathrm{d}S_i}{\mathrm{d}t} &= F_i(S_i, I_i, R_i) + \frac{1}{n}\sum_j \widetilde{m}_{ij}S_j - \frac{1}{n}\sum_j \widetilde{m}_{ji}S_i,\\
\frac{\mathrm{d}I_i}{\mathrm{d}t} &= G_i(S_i, I_i, R_i) + \frac{1}{n}\sum_j \widetilde{m}_{ij}I_j - \frac{1}{n}\sum_j \widetilde{m}_{ji}I_i, \qquad \text{[S2.7]}\\
\frac{\mathrm{d}R_i}{\mathrm{d}t} &= H_i(S_i, I_i, R_i) + \frac{1}{n}\sum_j \widetilde{m}_{ij}R_j - \frac{1}{n}\sum_j \widetilde{m}_{ji}R_i,
\end{aligned}$$

where S_i, I_i, and R_i are the densities of susceptible, infected, and recovered hosts in population i,

$$\begin{aligned}
F_i &= r_i\left(1 - \frac{N_i}{K_i}\right)N_i - \mu S_i + \eta I_i + \nu_i R_i - \beta S_i I_i,\\
G_i &= \beta S_i I_i - (\mu + \alpha + \eta + \gamma)I_i, \qquad \text{[S2.8]}\\
H_i &= \gamma I_i - (\mu + \nu_i)R_i,
\end{aligned}$$

describe the changes in the host densities in population i due to factors other than movements, and $N_i = S_i + I_i + R_i$ is the total host density in population i.

Below, we study how the evolution of the pathogen virulence α is affected by metapopulation heterogeneity in the parameters that characterize the host's local productivity (r_i and K_i), stability of immunity (ν_i), and movement patterns ($\widetilde{m}_{ij}$). With this replacement of symbols, we have

$$\widetilde{m}_{ij} = m_0 + \varepsilon\widetilde{m}'_{ij}.$$

We do not consider metapopulation heterogeneity in the other demographic and epidemiological parameters μ, α, β, η, and γ that affect the rate $\mathrm{d}I_i/\mathrm{d}t$ of change in the densities of infected hosts. This is because our analyses have revealed that metapopulation heterogeneity in these latter parameters affects virulence evolution in a more complex way, which we plan to describe in future research.

Invasion dynamics of new pathogen variants As in the single-population case summarized in S2.1, we consider a host that is in an endemic equilibrium with a resident pathogen characterized by its virulence α and transmission rate $\beta(\alpha)$. We denote by S_i^*, I_i^*, and R_i^* the equilibrium densities of susceptible, infected,

and recovered hosts in population $i = 1, \dots, n$. We consider the invasibility of a new pathogen variant that has a different virulence $\hat{\alpha}$ and transmission rate $\hat{\beta} = \beta(\hat{\alpha})$ than the resident. Denoting by y_i the density of hosts infected by the variant in population i, the linearized dynamics of these host densities, when the variant is rare, is

$$\frac{\mathrm{d}y_i}{\mathrm{d}t} = \beta(\hat{\alpha}) S_i^* y_i - (\mu + \hat{\alpha} + \eta + \gamma) y_i + \frac{1}{n} \sum\nolimits_j \widetilde{m}_{ij} y_j - \frac{1}{n} \sum\nolimits_j \widetilde{m}_{ji} y_i. \tag{S2.9}$$

This dynamics is expressed in vector form as $\mathrm{d}\boldsymbol{y}/\mathrm{d}t = \hat{\boldsymbol{J}}\boldsymbol{y}$, where $\boldsymbol{y} = (y_1, \dots, y_n)^\top$, and $\hat{\boldsymbol{J}}$ is the Jacobian at $\boldsymbol{y} = \boldsymbol{0}$,

$$\hat{\boldsymbol{J}} = \begin{pmatrix} \hat{a}_1 & b_{12} & \cdots & b_{1n} \\ b_{21} & \hat{a}_2 & \ddots & \vdots \\ \vdots & \ddots & \ddots & b_{n-1,n} \\ b_{n1} & \cdots & b_{n,n-1} & \hat{a}_n \end{pmatrix}, \tag{S2.10a}$$

with

$$\hat{a}_i = \beta(\hat{\alpha}) S_i^* - (\mu + \hat{\alpha} + \eta + \gamma) - \sum\nolimits_{j \neq i} \widetilde{m}_{ji}/n, \tag{S2.10b}$$

$$b_{ij} = \widetilde{m}_{ij}/n. \tag{S2.10c}$$

Invasion fitness, selection gradient, and fitness curvature The invasion fitness $w(\hat{\alpha}, \alpha)$ of the variant pathogen with virulence $\hat{\alpha}$ in the heterogeneous host metapopulation endemically infected by the resident pathogen with virulence α is given by the dominant eigenvalue $\hat{\lambda}$ of $\hat{\boldsymbol{J}}$,

$$w(\hat{\alpha}, \alpha) = \hat{\lambda} = \frac{\hat{\boldsymbol{v}}^\top \hat{\boldsymbol{J}} \hat{\boldsymbol{\phi}}}{\hat{\boldsymbol{v}}^\top \hat{\boldsymbol{\phi}}} = \hat{\boldsymbol{v}}^\top \hat{\boldsymbol{J}} \hat{\boldsymbol{\phi}}, \tag{S2.11}$$

where $\hat{\boldsymbol{v}}$ and $\hat{\boldsymbol{\phi}}$ are the corresponding left and right eigenvectors, i.e., $\hat{\boldsymbol{J}}\hat{\boldsymbol{\phi}} = \hat{\lambda}\hat{\boldsymbol{\phi}}$ and $\hat{\boldsymbol{v}}^\top \hat{\boldsymbol{J}} = \hat{\lambda}\hat{\boldsymbol{v}}^\top$. Here, $^\top$ denotes matrix transposition, and $\hat{\boldsymbol{v}}$ is normalized so that $\hat{\boldsymbol{v}}^\top \hat{\boldsymbol{\phi}} = 1$.

The selection gradient, Eq. (S2.4), and fitness curvature, Eq. (S2.5), in the metapopulation are then given by

$$\mathcal{S}(\alpha) = \left.\frac{\mathrm{d}\hat{\lambda}}{\mathrm{d}\hat{\alpha}}\right|_{\hat{\alpha}=\alpha} = \boldsymbol{v}^\top \left(\left.\frac{\mathrm{d}\hat{\boldsymbol{J}}}{\mathrm{d}\hat{\alpha}}\right|_{\hat{\alpha}=\alpha} \right) \boldsymbol{\phi}, \tag{S2.12}$$

$$\mathcal{D}(\alpha) = \left.\frac{\mathrm{d}^2\hat{\lambda}}{\mathrm{d}\hat{\alpha}^2}\right|_{\hat{\alpha}=\alpha} = \boldsymbol{v}^\top \left(\left.\frac{\mathrm{d}^2\hat{\boldsymbol{J}}}{\mathrm{d}\hat{\alpha}^2}\right|_{\hat{\alpha}=\alpha} \right) \boldsymbol{\phi}, \tag{S2.13}$$

where $\boldsymbol{v}$ and $\boldsymbol{\phi}$ are the left and right eigenvectors of $\boldsymbol{J} = \hat{\boldsymbol{J}}|_{\hat{\alpha}=\alpha}$ corresponding to the eigenvalue $\lambda = 0$ for $\hat{\alpha} = \alpha$, i.e., $\boldsymbol{J}\boldsymbol{\phi} = \boldsymbol{0}$ and $\boldsymbol{v}^\top \boldsymbol{J} = \boldsymbol{0}^\top$. In deriving Eq. (S2.12) and Eq. (S2.13), we use the eigenequations $\hat{\boldsymbol{J}}\hat{\boldsymbol{\phi}} = \hat{\lambda}\hat{\boldsymbol{\phi}}$ and $\hat{\boldsymbol{v}}^\top \hat{\boldsymbol{J}} = \hat{\lambda}\hat{\boldsymbol{v}}^\top$ and their $\hat{\alpha}$-differentiation evaluated at $\hat{\alpha} = \alpha$. The matrices $(\mathrm{d}\hat{\boldsymbol{J}}/\mathrm{d}\hat{\alpha})|_{\hat{\alpha}=\alpha}$ and $(\mathrm{d}^2\hat{\boldsymbol{J}}/\mathrm{d}\hat{\alpha}^2)|_{\hat{\alpha}=\alpha}$ are diagonal,

$$\left.\frac{\mathrm{d}\hat{\boldsymbol{J}}}{\mathrm{d}\hat{\alpha}}\right|_{\hat{\alpha}=\alpha} = \mathrm{diag}\left\{ \frac{\mathrm{d}\beta}{\mathrm{d}\alpha}(\alpha) S_1^* - 1, \dots, \frac{\mathrm{d}\beta}{\mathrm{d}\alpha}(\alpha) S_n^* - 1 \right\}, \tag{S2.14a}$$

$$\left.\frac{\mathrm{d}^2\hat{\boldsymbol{J}}}{\mathrm{d}\hat{\alpha}^2}\right|_{\hat{\alpha}=\alpha} = \mathrm{diag}\left\{ \frac{\mathrm{d}^2\beta}{\mathrm{d}\alpha^2}(\alpha) S_1^*, \dots, \frac{\mathrm{d}^2\beta}{\mathrm{d}\alpha^2}(\alpha) S_n^* \right\}, \tag{S2.14b}$$

where $\mathrm{diag}\{a_1, \dots, a_n\}$ denotes a diagonal matrix with diagonal elements $a_1, \dots, a_n$. Each of the diagonal elements of Eq. (S2.14) is given by, respectively, the local selection gradient and the local fitness curvature

that would apply in each of the local populations if they were isolated from all other local populations (compare these elements with Eqs. (S2.4) and (S2.5)). According to the aforementioned normalization convention $\boldsymbol{v}^\top \boldsymbol{\phi} = 1$, the selection gradient of metapopulation is expressed as the weighted sum of local selection gradient:

$$\begin{aligned}
\mathcal{S}(\alpha) &= \frac{\mathrm{d}\beta}{\mathrm{d}\alpha}(\alpha)\, \boldsymbol{v}^\top \begin{pmatrix} S_1^* & 0 & \cdots & 0 \\ 0 & S_2^* & \ddots & \vdots \\ \vdots & \ddots & \ddots & 0 \\ 0 & \cdots & 0 & S_n^* \end{pmatrix} \boldsymbol{\phi} - 1 \\
&= \frac{\mathrm{d}\beta}{\mathrm{d}\alpha}(\alpha) \sum_{i=1}^{n} v_i S_i^* \phi_i - 1 \\
&= \sum_{i=1}^{n} v_i \phi_i s_i, \qquad \text{[S2.15]}
\end{aligned}$$

where s_i is the local selection gradient at population i:

$$s_i = \frac{\mathrm{d}\beta}{\mathrm{d}\alpha} S_i^* - 1. \qquad \text{[S2.16]}$$

The fitness curvature of metapopulation is also expressed as the weighted sum of local fitness curvatures:

$$\begin{aligned}
\mathcal{D}(\alpha) &= \frac{\mathrm{d}^2\beta}{\mathrm{d}\alpha^2}(\alpha)\, \boldsymbol{v}^\top \begin{pmatrix} S_1^* & 0 & \cdots & 0 \\ 0 & S_2^* & \ddots & \vdots \\ \vdots & \ddots & \ddots & 0 \\ 0 & \cdots & 0 & S_n^* \end{pmatrix} \boldsymbol{\phi} \\
&= \frac{\mathrm{d}^2\beta}{\mathrm{d}\alpha^2}(\alpha) \sum_{i=1}^{n} v_i \phi_i S_i^* \\
&= \sum_{i=1}^{n} v_i \phi_i D_i, \qquad \text{[S2.17]}
\end{aligned}$$

where D_i is the local fitness curvature at population i:

$$D_i = \frac{\mathrm{d}^2\beta}{\mathrm{d}\alpha^2} S_i^*. \qquad \text{[S2.18]}$$

In Eq. (S2.15) and Eq. (S2.17), v_i and ϕ_i, respectively, are ith elements of the left and right eigenvectors $\boldsymbol{v}$ and $\boldsymbol{\phi}$.

We can now compare the selection gradient, Eq. (S2.15), and fitness curvature, Eq. (S2.17), for virulence evolution in heterogeneous metapopulations with the corresponding expressions, Eq. (S2.4) and Eq. (S2.5), for virulence evolution in single populations. This reveals that the equilibrium density S^* of susceptible hosts in a single populaftion is replaced by the weighted mean $\sum_i v_i S_i^* \phi_i$ of the equilibrium densities S_i^* of susceptible hosts in a metapopulation, where the weight of the ith local population is given by $v_i \phi_i$, i.e., by the product of the reproductive value v_i and the quasi-equilibrium density ϕ_i of the pathogen in the ith local population.

The remaining task is to express the eigenvectors $\boldsymbol{v}$ and $\boldsymbol{\phi}$ and the equilibrium densities S_i^* of susceptible hosts in terms of the heterogeneous parameters r_i, K_i, ν_i, and $\widetilde{m}_{ij}$. This is generally difficult but becomes analytically tractable if we assume that the deviations of these parameters from their metapopulation means are sufficiently small.

S2.3. Small-heterogeneity approximation for selection gradient in a metapopulation. In the previous subsection S2.2, we have derived the expressions Eq. (S2.15) and Eq. (S2.17), respectively, for the selection gradient and fitness curvature characterizing virulence evolution in a heterogeneous metapopulation. Here, we use these results to derive the explicit forms of the left and right eigenvectors of the Jacobian $\boldsymbol{J} = (\hat{\boldsymbol{J}})_{\hat{\alpha}=\alpha}$ of the dynamics of an invading variant pathogen evaluated for the variant having the same trait value, that is, virulence, as the resident, $\hat{\alpha} = \alpha$. In this case, the invasion fitness of the variant, given by the largest eigenvalue of $\boldsymbol{J}$, must be 0, and the corresponding left and right eigenvectors, $\boldsymbol{v}$ and $\boldsymbol{\phi}$, comprise as their elements the reproductive values and the equilibrium densities of hosts infected by the resident pathogen, respectively, across all local populations. Obtaining these quantities for arbitrary metapopulation parameters is difficult. We therefore adopt a strategy of expressing these quantities through series expansions for small degrees of metapopulation heterogeneity.

Expansion of parameters To obtain analytical results for the evolution of virulence in a heterogeneous metapopulation, we assume that the local birth rates, carrying capacities, immunity-loss rates, and movement rates vary slightly around their metapopulation means:

$$r_i = r_0 + \varepsilon r_i', \quad \text{[S2.19a]}$$
$$K_i = K_0 + \varepsilon K_i', \quad \text{[S2.19b]}$$
$$\nu_i = \nu_0 + \varepsilon \nu_i', \quad \text{[S2.19c]}$$
$$\widetilde{m}_{ij} = m_0 + \varepsilon \widetilde{m}_{ij}', \quad \text{[S2.19d]}$$

where $\varepsilon \ll 1$ is a small positive constant measuring the degree of metapopulation heterogeneity, r_0, K_0, ν_0, m_0 are the metapopulation means of the local birth rates r_i, carrying capacities K_i, immunity-loss rates ν_i, and movement rates $\widetilde{m}_{ij}$, respectively, and $\varepsilon r_i'$, $\varepsilon K_i'$, $\varepsilon \nu_i'$, and $\varepsilon \widetilde{m}_{ij}'$ are the deviations from the metapopulation means. These deviations have means of 0 and standard deviations equaling ε times the corresponding metapopulation means, so r_i', K_i', ν_i', and $\widetilde{m}_{ij}'$ are of order 1, i.e., of order 0 in ε, and ε is the coefficient of variation in r_i, K_i, ν_i, and $\widetilde{m}_{ij}$. By definition of the metapopulation means, we have $\sum_i r_i' = \sum_i K_i' = \sum_i \nu_i' = \sum_i \sum_j \widetilde{m}_{ij}' = 0$. Note that the movement rate from population j to i in the main text is defined as $m_{ij} = m_0/n + \varepsilon m_{ij}'$, but here in SI, we denote, for the sake of mathematical simplicity, m_{ij} and m_{ij}' in the main text by $\widetilde{m}_{ij}/n$ and $\widetilde{m}_{ij}'/n$, respectively, to yield [S2.19d].

Expansion of equilibrium local susceptible densities Under assumption Eq. (S2.19) on metapopulation heterogeneity, the equilibrium density of susceptible hosts in local population i can be expressed as

$$S_i^* = S_0^* + \varepsilon S_i^{*\prime} + \varepsilon^2 S_i^{*\prime\prime} + O(\varepsilon^3), \quad \text{[S2.20]}$$

where $S_0^* = (\mu+\alpha+\eta+\gamma)/\beta$ is the equilibrium density of susceptible hosts in a homogeneous metapopulation, i.e., for $\varepsilon = 0$, and $S_i^{*\prime}$ and $S_i^{*\prime\prime}$, respectively, are the polynomial coefficients in ε of its first-order and second-order deviations resulting from the deviations, Eq. (S2.19), of the conditions in local population i from their metapopulation means.

Expansion of Jacobian and eigenvectors We also express the Jacobian $\boldsymbol{J}$ of the invasion dynamics of a variant having the same trait value as the resident, as well as its left and right eigenvectors $\boldsymbol{v}$ and $\boldsymbol{\phi}$, through series expansions for a small degree ε of metapopulation heterogeneity:

$$\boldsymbol{J} = \boldsymbol{J_0} + \varepsilon \boldsymbol{J}' + \varepsilon^2 \boldsymbol{J}'' + O(\varepsilon^3), \quad \text{[S2.21]}$$
$$\boldsymbol{v} = \boldsymbol{v_0} + \varepsilon \boldsymbol{v}' + \varepsilon^2 \boldsymbol{v}'' + O(\varepsilon^3), \quad \text{[S2.22]}$$
$$\boldsymbol{\phi} = \boldsymbol{\phi_0} + \varepsilon \boldsymbol{\phi}' + \varepsilon^2 \boldsymbol{\phi}'' + O(\varepsilon^3), \quad \text{[S2.23]}$$

where all terms are defined and interpreted analogously to those in Eq. (S2.20).

Eigenequations of zeroth, first, and second orders By equating the polynomial coefficients of the same order ε^0, ε^1, or ε^2, the eigenequations $\boldsymbol{J}\boldsymbol{\phi} = \mathbf{0}$ and $\boldsymbol{v}^\top \boldsymbol{J} = \mathbf{0}$ yield, respectively,

$$\boldsymbol{J_0}\boldsymbol{\phi_0} = \mathbf{0}, \tag{S2.24}$$
$$\boldsymbol{J}'\boldsymbol{\phi_0} + \boldsymbol{J_0}\boldsymbol{\phi}' = \mathbf{0}, \tag{S2.25}$$
$$\boldsymbol{J}''\boldsymbol{\phi_0} + \boldsymbol{J}'\boldsymbol{\phi}' + \boldsymbol{J_0}\boldsymbol{\phi}'' = \mathbf{0}, \tag{S2.26}$$

and

$$\boldsymbol{v_0}^\top \boldsymbol{J_0} = \mathbf{0}^\top, \tag{S2.27}$$
$$\boldsymbol{v_0}^\top \boldsymbol{J}' + \boldsymbol{v}'^\top \boldsymbol{J_0} = \mathbf{0}^\top, \tag{S2.28}$$
$$\boldsymbol{v_0}^\top \boldsymbol{J}'' + \boldsymbol{v}'^\top \boldsymbol{J}' + \boldsymbol{v}''^\top \boldsymbol{J_0} = \mathbf{0}^\top, \tag{S2.29}$$

By evaluating Eq. (S2.10) for $\hat{\alpha} = \alpha$ and using $\widetilde{m}_{ij} = m_0 + \varepsilon \widetilde{m}'_{ij}$, $S^*_i = S^*_0 + \varepsilon S^{*\prime}_i + \ldots$, and $\beta(\alpha)S^*_0 - (\mu + \alpha + \eta + \gamma) = 0$, we obtain

$$\boldsymbol{J_0} = -m_0 \boldsymbol{I} + \frac{m_0}{n}\boldsymbol{U}, \tag{S2.30}$$

$$\boldsymbol{J}' = \beta \begin{pmatrix} S^{*\prime}_1 & 0 & \cdots & 0 \\ 0 & S^{*\prime}_2 & \ddots & \vdots \\ \vdots & \ddots & \ddots & 0 \\ 0 & \cdots & 0 & S^{*\prime}_n \end{pmatrix} + \frac{1}{n} \begin{pmatrix} \widetilde{m}'_{11} & \widetilde{m}'_{12} & \cdots & \widetilde{m}'_{1n} \\ \widetilde{m}'_{21} & \widetilde{m}'_{22} & \ddots & \vdots \\ \vdots & \ddots & \ddots & \widetilde{m}'_{n-1,n} \\ \widetilde{m}'_{n1} & \cdots & \widetilde{m}'_{n,n-1} & \widetilde{m}'_{nn} \end{pmatrix}, \tag{S2.31}$$

$$\boldsymbol{J}'' = \beta \begin{pmatrix} S^{*\prime\prime}_1 & 0 & \cdots & 0 \\ 0 & S^{*\prime\prime}_2 & \ddots & \vdots \\ \vdots & \ddots & \ddots & 0 \\ 0 & \cdots & 0 & S^{*\prime\prime}_n \end{pmatrix}, \tag{S2.32}$$

where $\boldsymbol{I}$ is the n-dimensional identity matrix, $\boldsymbol{U} = \mathbf{1}\mathbf{1}^\top$ is the $n \times n$ matrix of 1s, $\mathbf{1} = (1, \ldots, 1)^\top$ is the n-dimensional vector of 1s, and $\widetilde{m}'_{ii} = -\sum_{j \neq i} \widetilde{m}'_{ji}$.

Normalization of left and right eigenvectors We use the following two normalization conventions for the left and right eigenvectors: $\boldsymbol{v}^\top \boldsymbol{\phi} = 1$ and $\mathbf{1}^\top \boldsymbol{\phi} = 1$, where $\mathbf{1}$ is the vector of 1s. By equating the polynomial coefficients of the same order ε^0, ε^1, or ε^2, the two normalization equations yield, respectively,

$$\boldsymbol{v_0}^\top \boldsymbol{\phi_0} = 1, \tag{S2.33}$$
$$\boldsymbol{v_0}^\top \boldsymbol{\phi}' + \boldsymbol{v}'^\top \boldsymbol{\phi_0} = 0, \tag{S2.34}$$
$$\boldsymbol{v_0}^\top \boldsymbol{\phi}'' + \boldsymbol{v}'^\top \boldsymbol{\phi}' + \boldsymbol{v}''^\top \boldsymbol{\phi_0} = 0, \tag{S2.35}$$

and

$$\mathbf{1}^\top \boldsymbol{\phi_0} = 1, \tag{S2.36}$$
$$\mathbf{1}^\top \boldsymbol{\phi}' = 0, \tag{S2.37}$$
$$\mathbf{1}^\top \boldsymbol{\phi}'' = 0. \tag{S2.38}$$

Zeroth-order eigenvectors By substituting Eq. (S2.30) and Eq. (S2.36) into Eq. (S2.24), we have $\boldsymbol{J_0}\boldsymbol{\phi_0} = -m_0\boldsymbol{\phi_0} + (m_0/n)\mathbf{1}\mathbf{1}^\top \boldsymbol{\phi_0} = -m_0\boldsymbol{\phi_0} + (m_0/n_0)\mathbf{1} = \mathbf{0}$ and hence

$$\boldsymbol{\phi_0} = \frac{\mathbf{1}}{n} = \phi_0 \mathbf{1}, \tag{S2.39}$$

where $\phi_0 = 1/n$.

Similarly, by substituting Eq. (S2.30) and Eq. (S2.33) into Eq. (S2.27), we have $\boldsymbol{v_0}^\top \boldsymbol{J_0} = -m_0\boldsymbol{v_0}^\top + (m_0/n)\boldsymbol{v_0}^\top\mathbf{1}\mathbf{1}^\top = -m_0\boldsymbol{v_0}^\top + m_0\boldsymbol{v_0}^\top\boldsymbol{\phi_0}\mathbf{1}^\top = -m_0\boldsymbol{v_0}^\top + m_0\mathbf{1}^\top = \mathbf{0}^\top$ and hence

$$\boldsymbol{v_0} = \mathbf{1} = v_0\mathbf{1}, \tag{S2.40}$$

where $v_0 = 1$.

First-order eigenvectors Next, by substituting Eqs. (S2.30), (S2.31), (S2.37), and Eq. (S2.39) into Eq. (S2.25), we have $\boldsymbol{J'\phi_0}+\boldsymbol{J_0\phi'} = \boldsymbol{J'}\mathbf{1}/n - m_0\boldsymbol{\phi'} + (m_0/n)\mathbf{1}\mathbf{1}^\top\boldsymbol{\phi'} = \boldsymbol{J'}\mathbf{1}/n - m_0\boldsymbol{\phi'} = (\beta\boldsymbol{S^{*\prime}}+\boldsymbol{T'})/n - m_0\boldsymbol{\phi'} = \mathbf{0}$ and hence

$$\boldsymbol{\phi'} = \frac{\beta\boldsymbol{S^{*\prime}}+\boldsymbol{T'}}{nm_0} \qquad \text{or} \qquad \phi'_i = \frac{\beta S^{*\prime}_i + T'_i}{nm_0}, \tag{S2.41}$$

where $\boldsymbol{S^{*\prime}}$ is the vector of the coefficients of the first-order deviations of the equilibrium susceptible densities from their metapopulation average, i.e., $\boldsymbol{S^{*\prime}} = (S^{*\prime}_1, \ldots, S^{*\prime}_n)^\top$, and $\boldsymbol{T'} = (T'_1, \ldots, T'_n)^\top$ is the vector of the coefficients of the first-order deviations of the net movement inflow into population i from their metapopulation average of 0, i.e.,

$$T'_i = \frac{1}{n}\left(\sum_j \tilde{m}'_{ij} - \sum_j \tilde{m}'_{ji}\right). \tag{S2.42}$$

To obtain $\boldsymbol{v'}$, we first substitute Eqs. (S2.39) and (S2.40) into Eq. (S2.35) to have $\mathbf{1}^\top(\boldsymbol{S^{*\prime}}+\boldsymbol{T'})/nm_0 + \boldsymbol{v'}^\top\mathbf{1}/n = \boldsymbol{v'}^\top\mathbf{1}/n = 0$. Noting this and substituting Eqs. (S2.30), (S2.31), and (S2.40) into Eq. (S2.28), we have $\boldsymbol{v_0}^\top\boldsymbol{J'} + \boldsymbol{v'}^\top\boldsymbol{J_0} = \mathbf{1}^\top\boldsymbol{J'} - m_0\boldsymbol{v'} + (m_0/n)\boldsymbol{v'}\mathbf{1}\mathbf{1}^\top = \beta\boldsymbol{S^{*\prime}}^\top - m_0\boldsymbol{v'} = \mathbf{0}^\top$, and hence

$$\boldsymbol{v'} = \frac{\beta\boldsymbol{S^{*\prime}}}{m_0} \qquad \text{or} \qquad v'_i = \frac{\beta S^{*\prime}_i}{m_0}. \tag{S2.43}$$

Selection gradient The left and right eigenvectors Eq. (S2.22) and Eq. (S2.23) are now defined up to first order in ε by Eqs. (S2.39)–(S2.43). We then substitute these into Eq. (S2.15), which gives

$$\begin{aligned}
\mathcal{S}(\alpha) &= \frac{\mathrm{d}\beta}{\mathrm{d}\alpha}(\alpha)\,\boldsymbol{v}^\top\boldsymbol{S^*}\boldsymbol{\phi} - 1 = \sum_{i=1}^n v_i\phi_i s_i \\
&= \sum_{i=1}^n \left(v_0 + \varepsilon v'_i + \varepsilon^2 v''_i + O(\varepsilon^3)\right)\left(\phi_0 + \varepsilon\phi'_i + \varepsilon^2\phi''_i + O(\varepsilon^3)\right)\left(s_0 + \varepsilon s'_i + \varepsilon^2 s''_i + O(\varepsilon^3)\right) \\
&= \mathcal{S}_0 + \varepsilon\mathcal{S}_1 + \varepsilon^2\mathcal{S}_2 + O(\varepsilon^3),
\end{aligned} \tag{S2.44}$$

where s_0, $\varepsilon s'_i$, and $\varepsilon^2 s''_i$ are the zeroth, first, and second order deviations of *local* selection gradient at population i:

$$s_0 = \frac{\mathrm{d}\beta}{\mathrm{d}\alpha}S^*_0 - 1, \qquad s'_i = \frac{\mathrm{d}\beta}{\mathrm{d}\alpha}S^{*\prime}_i, \qquad s''_i = \frac{\mathrm{d}\beta}{\mathrm{d}\alpha}S^{*\prime\prime}_i. \tag{S2.45}$$

Equating the coefficients of the zeroth-order terms in ε of Eq. (S2.44) yields

$$\mathcal{S}_0 = \sum_{i=1}^n v_0\phi_0 s_0 = s_0 = \frac{\mathrm{d}\beta}{\mathrm{d}\alpha}(\alpha)S^*_0 - 1. \tag{S2.46}$$

Equating the coefficients of the first-order terms in ε of Eq. (S2.44) yields

$$\begin{aligned}
\mathcal{S}_1 &= \sum_{i=1}^n (v'_i\phi_0 s_0 + v_0\phi'_i s_0 + v_0\phi_0 s'_i) \\
&= v_0\phi_0\sum_{i=1}^n s'_i \\
&= 0,
\end{aligned} \tag{S2.47}$$

where the normalization relationship Eq. (S2.34) is used in the second line, and $\sum_{i=1}^{n} s_i' = \frac{\mathrm{d}\beta}{\mathrm{d}\alpha}\sum_{i=1}^{n} S_i^{*\prime} = 0$ is used in the last line. Equating the coefficients of the second-order terms in ε of Eq. (S2.44) yields

$$
\begin{aligned}
\mathcal{S}_2 &= \sum_{i=1}^{n}\left[s_0\left(v_i''\phi_0 + v_i'\phi_i' + v_0\phi_i''\right) + s_i'(v_i'\phi_0 + v_0\phi_i') + s_i''v_0\phi_0\right] \\
&= \sum_{i=1}^{n}\left(\frac{1}{n}s_i'v_i' + s_i'\phi_i' + \frac{1}{n}s_i''\right) \\
&= \frac{1}{n}\sum_{i=1}^{n} v_i's_i',
\end{aligned} \tag{S2.48}
$$

where the normalization condition Eq. (S2.35) is used in the second line, and $\sum_{i=1}^{n}(s_i'\phi_i' + \frac{1}{n}s_i'') = 0$ is used in the third line, which in turn follows from multiplying $\boldsymbol{v_0}^\top$ from the left to Eq. (S2.26) and noting $\boldsymbol{v_0}^\top\boldsymbol{J_0} = \boldsymbol{0}^\top$, $\boldsymbol{v}_0 = \boldsymbol{1}$, $\boldsymbol{\phi_0} = \boldsymbol{1}/n$, $\boldsymbol{J'} = \beta\mathrm{diag}(S_1^{*\prime},\cdots,S_n^{*\prime}) = \beta\left(\frac{\mathrm{d}\beta}{\mathrm{d}\alpha}\right)^{-1}\mathrm{diag}(s_1',\cdots,s_n')$, and $\boldsymbol{J''} = \beta\mathrm{diag}(S_1^{*\prime\prime},\cdots,S_n^{*\prime\prime}) = \beta\left(\frac{\mathrm{d}\beta}{\mathrm{d}\alpha}\right)^{-1}\mathrm{diag}(s_1'',\cdots,s_n'')$:

$$
\begin{aligned}
\boldsymbol{v_0}^\top\left(\boldsymbol{J''}\boldsymbol{\phi_0} + \boldsymbol{J'}\boldsymbol{\phi'} + \boldsymbol{J_0}\boldsymbol{\phi''}\right) &= \boldsymbol{v_0}^\top\boldsymbol{J''}\boldsymbol{\phi_0} + \boldsymbol{v_0}^\top\boldsymbol{J'}\boldsymbol{\phi'} \\
&= \beta\left(\frac{\mathrm{d}\beta}{\mathrm{d}\alpha}\right)^{-1}\sum_{i=1}^{n}\left(\frac{1}{n}s_i'' + s_i'\phi_i'\right) = 0.
\end{aligned} \tag{S2.49}
$$

To conclude, we have determined the selection gradient Eq. (S2.44) for the evolution of pathogen virulence in heterogeneous metapopulations up to second-order terms in ε,

$$
\begin{aligned}
\mathcal{S}(\alpha) &= \mathcal{S}_0(\alpha) + \varepsilon^2\frac{1}{n}\boldsymbol{v'}^\top\left(\frac{\mathrm{d}\beta}{\mathrm{d}\alpha}\boldsymbol{S^{*\prime}}\right) \\
&= \mathcal{S}_0(\alpha) + \varepsilon^2\frac{\mathrm{d}\beta}{\mathrm{d}\alpha}\frac{\beta}{m_0}\frac{1}{n}|\boldsymbol{S^{*\prime}}|^2,
\end{aligned} \tag{S2.50}
$$

where $\mathcal{S}_0(\alpha) = (\mathrm{d}\beta/\mathrm{d}\alpha)S_0^* - 1$ is the selection gradient in the corresponding homogeneous metapopulation, and Eq. (S2.43) is substituted in the second line. As the second term in Eq. (S2.50) is always positive as long as $\boldsymbol{S^{*\prime}} \neq \boldsymbol{0}$ (i.e., as long as there is metapopulation heterogeneity among equilibrium susceptible populations), Eq. (S2.50) implies that at the ESS virulence of the homogeneous metapopulation the selection gradient in the heterogeneous metapopulation is always positive. In other words, metapopulation heterogeneity always selects for increased ESS virulence.

S2.4. Increment of ESS virulence. We now derive the increment of ESS virulence in a heterogeneous metapopulation relative to that in the corresponding homogeneous metapopulation or single population. We denote by α^* the ESS virulence in the heterogeneous metapopulation and by α_0^* the ESS virulence in the corresponding homogeneous metapopulation or single population. According to Eq. (S2.4), α_0^* satisfies

$$
\mathcal{S}_0(\alpha_0^*) = \frac{\mathrm{d}\beta}{\mathrm{d}\alpha}(\alpha_0^*)S_0^* - 1 = 0. \tag{S2.51}
$$

According to Eq. (S2.50), α^* satisfies

$$
\mathcal{S}(\alpha^*) = \mathcal{S}_0(\alpha^*) + \varepsilon^2\left(\frac{\mathrm{d}\beta}{\mathrm{d}\alpha}\frac{\beta}{m_0}\frac{1}{n}|\boldsymbol{S^{*\prime}}|^2\right)_{\alpha=\alpha^*} = 0. \tag{S2.52}
$$

We evaluate $\mathcal{S}_0(\alpha^*)$ by substituting $\alpha^* = \alpha_0^* + \delta\alpha^*$, expanding with respect to small $\delta\alpha^* = O(\varepsilon^2)$ and ε, and using that $\mathcal{S}_0(\alpha_0^*) = 0$ and S_0^* is minimized at $\alpha = \alpha_0^*$,

$$\begin{aligned}\mathcal{S}_0(\alpha_0^* + \delta\alpha^*) &= \mathcal{S}_0(\alpha_0^*) + \frac{\mathrm{d}}{\mathrm{d}\alpha}\left(\frac{\mathrm{d}\beta}{\mathrm{d}\alpha}S_0^* - 1\right)\bigg|_{\alpha=\alpha_0^*+\delta\alpha^*}\delta\alpha^* + O(\varepsilon^3)\\ &= \left(\frac{\mathrm{d}^2\beta}{\mathrm{d}\alpha^2}S_0^* + \frac{\mathrm{d}\beta}{\mathrm{d}\alpha}\frac{\mathrm{d}S_0^*}{\mathrm{d}\alpha}\right)_{\alpha=\alpha_0^*}\delta\alpha^* + O(\varepsilon^3)\\ &= \left(\frac{\mathrm{d}^2\beta}{\mathrm{d}\alpha^2}S_0^*\right)_{\alpha=\alpha_0^*}\delta\alpha^* + O(\varepsilon^3).\end{aligned}$$

Substituting this result into Eq. (S2.52) yields

$$\mathcal{S}(\alpha^*) = \left(\frac{\mathrm{d}^2\beta}{\mathrm{d}\alpha^2}S_0^*\,\delta\alpha^* + \varepsilon^2\frac{\mathrm{d}\beta}{\mathrm{d}\alpha}\frac{\beta}{m_0}\frac{1}{n}|\boldsymbol{S}^{*\prime}|^2\right)_{\alpha=\alpha_0^*} + O(\varepsilon^3) = 0. \qquad \text{[S2.53]}$$

From this, the increment $\delta\alpha^*$ of ESS virulence is obtained as

$$\begin{aligned}\delta\alpha^* &= \varepsilon^2\left(\frac{\mathrm{d}\beta}{\mathrm{d}\alpha}\frac{\beta}{m_0}\left(-\frac{\mathrm{d}^2\beta}{\mathrm{d}\alpha^2}\right)^{-1}\frac{1}{S_0^*}\frac{1}{n}|\boldsymbol{S}^{*\prime}|^2\right)_{\alpha=\alpha_0^*}\\ &= \varepsilon^2\left(\frac{\beta}{m_0}\left(-\frac{\mathrm{d}^2\beta}{\mathrm{d}\alpha^2}\right)^{-1}\frac{1}{n}\frac{|\boldsymbol{S}^{*\prime}|^2}{S_0^{*2}}\right)_{\alpha=\alpha_0^*},\end{aligned} \qquad \text{[S2.54]}$$

where $(\mathrm{d}\beta/\mathrm{d}\alpha)S_0^* = 1$, which follows from Eq. (S2.51), is used in the last line

Thus, the increment of the ESS virulence is proportional to $|\boldsymbol{S}^{*\prime}|^2/n = (1/n)\sum_{i=1}^n (S_i^{*\prime})^2$, i.e., to the average squared coefficients of the first-order deviations of the equilibrium densities of susceptible hosts from their metapopulation mean. Therefore, the larger the degree of metapopulation heterogeneity in terms of the equilibrium densities of susceptible hosts, the larger the increment of the ESS virulence of the pathogen.

S2.5. Dependence on local conditions. According to Eq. (S2.50), (...), and Eq. (S2.54), respectively, the selection gradient, the fitness curvature, and the increment of the ESS virulence in a heterogeneous metapopulation are expressed as functions of the coefficients of the first-order deviations of the equilibrium densities of susceptible hosts, $S_i^{*\prime}$ for $i = 1, \ldots, n$, relative to the corresponding homogeneous metapopulation.

In Appendix S3, we show that these coefficients of the first-order deviations of the equilibrium densities of susceptible hosts relative to the corresponding homogeneous metapopulation, and thus, relative to the metapopulation mean $S_0^* = \sum_i S_i^*/n$, can be expressed as

$$\frac{S_i^{*\prime}}{S_0^*} = \sum_x q_x \frac{x_i'}{x_0}, \qquad \text{[S2.55]}$$

where x_i' is the coefficient of the first-order deviation of the birth rate r_i', carrying capacity K_i', immunity-loss rate ν_i', or net movement inflow $T_i' = \sum_j(\widetilde{m}_{ij}' - \widetilde{m}_{ji}')/n$ in population i, as defined in Eq. (S2.19), x_0 is the corresponding metapopulation mean, i.e., $r_0 = \sum_i r_i/n$, $K_0 = \sum_i K_i/n$, $\nu_0 = \sum_i \nu_i/n$, or $m_0 = \sum_i\sum_j \widetilde{m}_{ij}/n^2$, and q_x is the elasticity $q_x = \partial(S_i^*/S_0^*)/\partial(x_i/x_0)$ of the equilibrium density of susceptible hosts with respect to the parameter x, explicit results for which are derived in Appendix S3.

S2.6. Multiple heterogeneities. The results so far can readily be extended to situations in which multiple types of metapopulation heterogeneities are acting together simultaneously. Substituting Eq. (S2.55) into Eq. (S2.54) and introducing the shorthand

$$\Theta = \left(\frac{\beta}{m_0 \alpha} \left(-\frac{\mathrm{d}^2 \beta}{\mathrm{d}\alpha^2} \right)^{-1} \right) \Bigg|_{\alpha=\alpha_0^*}, \tag{S2.56}$$

we obtain the relative increment of the ESS virulence as

$$\frac{\delta\alpha^*}{\alpha_0^*} = \varepsilon^2 \Theta \left(\frac{1}{n} \sum_i \left(\frac{S_i^{*\prime}}{S_0^*} \right)^2 \right) \Bigg|_{\alpha=\alpha_0^*} = \varepsilon^2 \Theta \left(\frac{1}{n} \sum_i \left(\sum_x q_x \frac{x_i'}{x_0} \right)^2 \right) \Bigg|_{\alpha=\alpha_0^*}$$

$$= \varepsilon^2 \Theta \left(\sum_x {q_x}^2 \frac{1}{n} \sum_i \left(\frac{x_i'}{x_0} \right)^2 + 2 \sum_x \sum_{y \neq x} \frac{1}{n} \sum_i \sum_j \frac{x_i' y_j'}{x_0 y_0} \right) \Bigg|_{\alpha=\alpha_0^*} \tag{S2.57}$$

$$= \Theta \left(\sum_x {q_x}^2 \operatorname{Var}\left(\frac{x}{x_0} \right) + 2 \sum_x \sum_{y \neq x} q_x q_y \operatorname{Cov}\left(\frac{x}{x_0}, \frac{y}{y_0} \right) \right) \Bigg|_{\alpha=\alpha_0^*}, \tag{S2.58}$$

where the summation indices x and y run over all four potentially heterogeneous parameters, i.e., the birth rate r, the carrying capacity K, the immunity-loss rate ν, and the net movement inflow T, while x_0 and y_0 denote the corresponding metapopulation means r_0, K_0, ν_0, and m_0.

S2.7. Additional heterogeneities . A similar extension is possible for situations in which an additional metapopulation heterogeneity is acting on an already heterogeneous metapopulation. We denote by $\varepsilon x_i'$ the local deviation of parameter x in population i from its metapopulation average x_0, with $\sum_i x_i' = 0$, and by $\varepsilon \tilde{x}_i'$ an additional local deviation added in population i, again with $\sum_i \tilde{x}_i' = 0$. We then ask how this additional metapopulation heterogeneity affects the evolution of virulence. d As the total deviation in population i of parameter x from its mean is $\varepsilon(x_i' + \tilde{x}_i')$, the equilibrium density S_i^* of susceptible hosts relative to that in the corresponding homogeneous metapopulation is given by

$$\frac{S_i^*}{S_0^*} = 1 + \varepsilon q_x \frac{x_i' + \tilde{x}_i'}{x_0} \tag{S2.59}$$

or

$$\frac{S_i^{*\prime}}{S_0^*} = q_x \frac{x_i' + \tilde{x}_i'}{x_0}. \tag{S2.60}$$

From this, we immediately have an expression for the increment of the ESS virulence due to the joint action of the two heterogeneities with $x_i = x_0 + \varepsilon(x_i' + \tilde{x}_i')$,

$$\frac{\delta\alpha^*}{\alpha_0^*} = \Theta\, q_x^2 \operatorname{Var}\left(\varepsilon \frac{x_i' + \tilde{x}_i'}{x_0} \right)$$

$$= \Theta\, q_x^2 \left[\operatorname{Var}\left(\frac{x}{x_0} \right) + \operatorname{Cov}\left(\frac{x}{x_0}, \frac{\tilde{x}}{x_0} \right) + \operatorname{Var}\left(\frac{\tilde{x}}{x_0} \right) \right], \tag{S2.61}$$

where all terms on the right-hand sides are evaluated at $\alpha = \alpha_0^*$, Θ is the shorthand defined in Eq. (S2.56), $\operatorname{Var}(x/x_0)$ and $\operatorname{Var}(\tilde{x}/x_0)$ are the variances of the scaled primary and additional heterogeneities in the parameter x across local populations, and $\operatorname{Cov}(x/x_0, \tilde{x}/x_0)$ is the corresponding covariance between the scaled primary and additional heterogeneities across local populations.

S3. Equilibrium densities for small heterogeneities

S3.1. Endemic equilibrium. The equilibrium densities $\boldsymbol{x_i} = (S_i, I_i, R_i)^\top$ of metapopulation epidemiological dynamics Eq. (S2.7) in local population i with birth rate, carrying capacity, immunity-loss rate $\boldsymbol{\theta_i} = (r_i, K_i, \nu_i)^\top$ and movement rates $\widetilde{m}_{ij}$ from population j and $\widetilde{m}_{ji}$ to population j ($j = 1, \ldots, n$) satisfy

$$
\begin{aligned}
F(\boldsymbol{x_i}, \boldsymbol{\theta_i}) + \frac{1}{n}\left(\sum_j \widetilde{m}_{ij} S_j - \sum_j \widetilde{m}_{ji} S_i\right) &= 0, \\
G(\boldsymbol{x_i}, \boldsymbol{\theta_i}) + \frac{1}{n}\left(\sum_j \widetilde{m}_{ij} I_j - \sum_j \widetilde{m}_{ji} I_i\right) &= 0, \\
H(\boldsymbol{x_i}, \boldsymbol{\theta_i}) + \frac{1}{n}\left(\sum_j \widetilde{m}_{ij} R_j - \sum_j \widetilde{m}_{ji} R_i\right) &= 0,
\end{aligned}
\tag{S3.1}
$$

($i = 1, \ldots, n$), where, with $\boldsymbol{x} = (S, I, R)^\top$ and $\boldsymbol{\theta} = (r, K, \nu)^\top$,

$$
\begin{aligned}
F(\boldsymbol{x}, \boldsymbol{\theta}) &= r\,(1 - N/K)\,N - \mu S + \eta I + \nu R - \beta S I, \\
G(\boldsymbol{x}, \boldsymbol{\theta}) &= \beta S I - (\mu + \alpha + \eta + \gamma) I, \\
H(\boldsymbol{x}, \boldsymbol{\theta}) &= \gamma I - (\mu + \nu) R,
\end{aligned}
\tag{S3.2}
$$

where $N = S + I + R$. The equilibrium densities $\boldsymbol{x_0} = (S_0^*, I_0^*, R_0^*)^\top$ in homogeneous metapopulation with $\boldsymbol{\theta_i} = \boldsymbol{\theta_0} = (r_0, K_0, \nu_0)^\top$ for all i and $\widetilde{m}_{ij} = m_0$ for all i, j satisfy

$$
\begin{aligned}
F(\boldsymbol{x_0}, \boldsymbol{\theta_0}) &= 0, \\
G(\boldsymbol{x_0}, \boldsymbol{\theta_0}) &= 0, \\
H(\boldsymbol{x_0}, \boldsymbol{\theta_0}) &= 0.
\end{aligned}
\tag{S3.3}
$$

S3.2. Linear response to local conditions. If we introduce small metapopulation heterogeneities ($\boldsymbol{\theta_i} = \boldsymbol{\theta_0} + \varepsilon \boldsymbol{\theta_i'}$, where $\boldsymbol{\theta_i'} = (r_i', K_i', \nu_i')^\top$, and $\widetilde{m}_{ij} = m_0 + \varepsilon \widetilde{m}_{ij}'$) around metapopulation means ($\boldsymbol{\theta_0}$ and m_0), the equilibrium densities are also modulated from those in homogeneous metapopulation $\boldsymbol{x_0}$ as $\boldsymbol{x_i} = \boldsymbol{x_0} + \varepsilon \boldsymbol{x_i'} + O(\varepsilon^2)$, where $\boldsymbol{x_i'} = (S_i^{*\prime}, I_i^{*\prime}, R_i^{*\prime})^\top$. Substituting these into Eq. (S3.1), we have Eq. (S3.3) for the zeroth order terms of ε. The first-order terms of ε-expansion of Eq. (S3.1) is

$$
\begin{aligned}
\left(\frac{\partial F}{\partial S}\right)_0 S_i^{*\prime} + \left(\frac{\partial F}{\partial I}\right)_0 I_i^{*\prime} + \left(\frac{\partial F}{\partial R}\right)_0 R_i^{*\prime} + m_0\left(\frac{1}{n}\sum_j S_j^{*\prime} - S_i^{*\prime}\right) \\
+ \left(\frac{\partial F}{\partial r}\right)_0 r_i' + \left(\frac{\partial F}{\partial K}\right)_0 K_i' + \left(\frac{\partial F}{\partial \nu}\right)_0 \nu_i' + S_0^* T_i' = 0,
\end{aligned}
\tag{S3.4}
$$

$$
\left(\frac{\partial G}{\partial S}\right)_0 S_i^{*\prime} + m_0\left(\frac{1}{n}\sum_j I_j^{*\prime} - I_i^{*\prime}\right) + I_0^* T_i' = 0,
\tag{S3.5}
$$

$$
\left(\frac{\partial H}{\partial I}\right)_0 I_i^{*\prime} + \left(\frac{\partial H}{\partial R}\right)_0 R_i^{*\prime} + m_0\left(\frac{1}{n}\sum_j R_j^{*\prime} - R_i^{*\prime}\right) + \left(\frac{\partial H}{\partial \nu}\right)_0 \nu_i' + R_0^* T_i' = 0,
\tag{S3.6}
$$

where $\left(\frac{\partial F}{\partial S}\right)_0$ etc are the partial derivatives evaluated at homogeneous case, $\boldsymbol{x} = \boldsymbol{x_0}$, $\boldsymbol{\theta} = \boldsymbol{\theta_0}$, and $\widetilde{m}_{ij} = m_0$, and $T_i' = \left(\sum_j \widetilde{m}_{ij}' - \sum_j \widetilde{m}_{ji}'\right)/n$ denotes the relative net movement into population i.

S3.3. Sum of first-order deviations. Summing all terms in Eq. (S3.4)–Eq. (S3.6) for all i, and noting $\sum_i r_i' = \sum_i K_i' = \sum_i \nu_i' = \sum_i T_i' = 0$, we see that

$$\boldsymbol{\Lambda_0} \begin{pmatrix} \sum_i S_i^{*\prime} \\ \sum_i I_i^{*\prime} \\ \sum_i R_i^{*\prime} \end{pmatrix} = \mathbf{0}, \qquad \text{[S3.7]}$$

where

$$\boldsymbol{\Lambda_0} = \begin{pmatrix} \left(\frac{\partial F}{\partial S}\right)_0 & \left(\frac{\partial F}{\partial I}\right)_0 & \left(\frac{\partial F}{\partial R}\right)_0 \\ \left(\frac{\partial G}{\partial S}\right)_0 & 0 & 0 \\ 0 & \left(\frac{\partial H}{\partial I}\right)_0 & \left(\frac{\partial H}{\partial R}\right)_0 \end{pmatrix} = \begin{pmatrix} F_S & F_I & F_R \\ G_S & 0 & 0 \\ 0 & H_I & H_R \end{pmatrix}, \qquad \text{[S3.8]}$$

where the last equality is for notational simplicity. Explicit forms in each element are given by

$$\begin{aligned} &F_S = \rho_0 - \mu - \beta I_0^*, \quad F_I = \rho_0 + \eta - \beta S_0^*, \quad F_R = \rho_0 + \nu_0, \\ &G_S = \beta I_0^*, \quad H_I = \gamma, \quad H_R = -(\mu + \nu_0) \end{aligned} \qquad \text{[S3.9]}$$

where $\rho_0 = r_0(1 - 2N_0^*/K_0)$. From this, we conclude that if $\boldsymbol{\Lambda_0}$ is invertible, the sum of first-order deviations of local susceptible, infected, and recovered densities over metapopulation respectively are zero:

$$\sum_i S_i^{*\prime} = \sum_i I_i^{*\prime} = \sum_i R_i^{*\prime} = 0. \qquad \text{[S3.10]}$$

S3.4. First-order deviations of equilibrium densities. Substituting Eq. (S3.10) into Eq. (S3.4)–Eq. (S3.6), we have

$$(\boldsymbol{\Lambda_0} - m_0 \boldsymbol{I})\boldsymbol{x_i'} + \left(\frac{\partial F}{\partial r}\right)_0 \boldsymbol{e_1} r_i' + \left(\frac{\partial F}{\partial K}\right)_0 \boldsymbol{e_1} K_i' + \left[\left(\frac{\partial F}{\partial \nu}\right)_0 \boldsymbol{e_1} + \left(\frac{\partial H}{\partial \nu}\right)_0 \boldsymbol{e_3}\right] \nu_i' + \boldsymbol{x_0} T_i' = \mathbf{0}, \qquad \text{[S3.11]}$$

for each i $(i = 1, \ldots, n)$, where $\boldsymbol{e_1} = (1, 0, 0)^\top$, $\boldsymbol{e_3} = (0, 0, 1)^\top$, and $\boldsymbol{x_0} = (S_0^*, I_0^*, R_0^*)^\top$ and

$$\begin{aligned} &\left(\frac{\partial F}{\partial r}\right)_0 = (1 - N_0^*/K_0)N_0^*, \quad \left(\frac{\partial F}{\partial K}\right)_0 = -r_0 {N_0^*}^2/{K_0}^2, \\ &\left(\frac{\partial F}{\partial \nu}\right)_0 = -\left(\frac{\partial H}{\partial \nu}\right)_0 = R_0^*. \end{aligned} \qquad \text{[S3.12]}$$

Therefore, by letting $\boldsymbol{A_0} = m_0 \boldsymbol{I} - \boldsymbol{\Lambda_0}$,

$$\boldsymbol{x_i'} = \boldsymbol{A_0}^{-1} \left(\left(\frac{\partial F}{\partial r}\right)_0 \boldsymbol{e_1} r_i' + \left(\frac{\partial F}{\partial K}\right)_0 \boldsymbol{e_1} K_i' + R_0^*(\boldsymbol{e_1} - \boldsymbol{e_3})\nu_i' + \boldsymbol{x_0} T_i' \right), \qquad \text{[S3.13]}$$

where we used $\left(\frac{\partial F}{\partial \nu}\right)_0 = R_0^*$ and $\left(\frac{\partial H}{\partial \nu}\right)_0 = -R_0^*$. The inverse of $\boldsymbol{A_0}$ is expressed as

$$\begin{aligned} \boldsymbol{A_0}^{-1} &= \begin{pmatrix} m_0 - F_S & -F_I & -F_R \\ -G_S & m_0 & 0 \\ 0 & -H_I & m_0 - H_R \end{pmatrix}^{-1} \\ &= \frac{1}{|\boldsymbol{A_0}|} \begin{pmatrix} m_0(m_0 - H_R) & F_I(m_0 - H_R) + F_R H_I & m_0 F_R \\ G_S(m_0 - H_R) & (m_0 - F_S)(m_0 - H_R) & F_R G_S \\ G_S H_I & (m_0 - F_S) H_I & (m_0 - F_S) m_0 - F_I G_S \end{pmatrix} \end{aligned} \qquad \text{[S3.14]}$$

where

$$|\boldsymbol{A_0}| = m_0(m_0 - F_S)(m_0 - H_R) - F_R G_S H_I - F_I G_S (m_0 - H_R). \qquad \text{[S3.15]}$$

S3.5. Elasticity to local conditions. Equation Eq. (S3.13) is rewritten in non-dimensional form as

$$\frac{\boldsymbol{x_i'}}{S_0^*} = \boldsymbol{q_r}\frac{r_i'}{r_0} + \boldsymbol{q_K}\frac{K_i'}{K_0} + \boldsymbol{q_c}\frac{\nu_i'}{\nu_0} + \boldsymbol{q_T}\frac{T_i'}{m_0} \tag{S3.16}$$

where

$$\boldsymbol{q_r} = \frac{r_0}{S_0^*}\left(\frac{\partial F}{\partial r}\right)_0 \boldsymbol{A_0}^{-1}\boldsymbol{e_1}, \tag{S3.17}$$

$$\boldsymbol{q_K} = \frac{K_0}{S_0^*}\left(\frac{\partial F}{\partial K}\right)_0 \boldsymbol{A_0}^{-1}\boldsymbol{e_1}, \tag{S3.18}$$

$$\boldsymbol{q_\nu} = \frac{\nu_0}{S_0^*}\left[\left(\frac{\partial F}{\partial \nu}\right)_0 \boldsymbol{A_0}^{-1}\boldsymbol{e_1} + \left(\frac{\partial H}{\partial \nu}\right)_0 \boldsymbol{A_0}^{-1}\boldsymbol{e_3}\right], \tag{S3.19}$$

$$\boldsymbol{q_T} = \frac{m_0}{S_0^*}\boldsymbol{A_0}^{-1}\boldsymbol{x_0}, \tag{S3.20}$$

Elasticity of susceptible density Denoting the first element of $\boldsymbol{q_x}$ by q_x, the first-order deviation in the equilibrium density of susceptible hosts, $S_i^{*\prime}$, that plays a key role in the evolution of virulence, is

$$\frac{S_i^{*\prime}}{S_0^*} = q_r\frac{r_i'}{r_0} + q_K\frac{K_i'}{K_0} + q_\nu\frac{\nu_i'}{\nu_0} + q_T\frac{T_i'}{m_0}. \tag{S3.21}$$

where

$$q_r = \frac{r_0}{S_0^*}\left(\frac{\partial F}{\partial r}\right)_0 (\boldsymbol{A_0}^{-1}\boldsymbol{e_1})_1 = \frac{r_0}{S_0^*}\left(1 - \frac{N_0^*}{K_0}\right) N_0^* \frac{m_0(m_0+\mu+\nu_0)}{|\boldsymbol{A_0}|}, \tag{S3.22a}$$

$$q_K = \frac{K_0}{S_0^*}\left(\frac{\partial F}{\partial K}\right)_0 (\boldsymbol{A_0}^{-1}\boldsymbol{e_1})_1 = \frac{K_0}{S_0^*}\frac{r_0 N_0^{*2}}{K_0{}^2}\frac{m_0(m_0+\mu+\nu_0)}{|\boldsymbol{A_0}|}, \tag{S3.22b}$$

$$q_\nu = \frac{\nu_0}{S_0^*}R_0^*(\boldsymbol{A_0}^{-1}(\boldsymbol{e_1}-\boldsymbol{e_3}))_1 = \frac{\nu_0}{S_0^*}R_0^*\frac{m_0\left\{(m_0+\mu+\nu_0)-F_R\right\}}{|\boldsymbol{A_0}|}, \tag{S3.22c}$$

$$\begin{aligned} q_T &= \frac{m_0}{S_0^*}(\boldsymbol{A_0}^{-1}\boldsymbol{x_0})_1 \\ &= \frac{m_0^2(m_0+\mu+\nu_0)}{|\boldsymbol{A_0}|} + \frac{I_0^*}{S_0^*}\frac{m_0(m_0+\mu+\nu_0)F_I + m_0\gamma F_R}{|\boldsymbol{A_0}|} + \frac{R_0^*}{S_0^*}\frac{m_0^2 F_R}{|\boldsymbol{A_0}|}, \end{aligned} \tag{S3.22d}$$

S3.6. Elasticities in some limiting cases.

SIS model with constant local host population densities Though the elasticities of the equilibrium local susceptible densities to heterogeneous parameters have already been defined by Eq. (S3.22), to see their dependencies on the other parameters more explicitly, we here consider the simplest epidemiological model in which the host growth rate is sufficiently large ($r \to \infty$) so that a vacancy open by the mortality of host is immediately filled by a susceptible newborn. Then, the local host densities are maintained at their (heterogeneous) carrying capacities: $N_i = K_i = K_0 + \varepsilon K_i'$ (with $\sum_i K_i' = 0$). We here further focus on an SIS model, $\gamma = 0$, so that there is no immune class ($R_i = 0$). An SIRS model with $\gamma > 0$ is examined in the next subsection. The epidemiological dynamics is then

$$\frac{\mathrm{d}I_i}{\mathrm{d}t} = \beta(K_i - I_i)I_i - (\mu+\alpha+\eta)I_i + \frac{1}{n}\sum_j \widetilde{m}_{ij}I_j - \frac{1}{n}\sum_j \widetilde{m}_{ji}I_i. \tag{S3.23}$$

Heterogeneity in movement is introduced too as in the previous section: $\widetilde{m}_{ij} = m_0 + \varepsilon\widetilde{m}_{ij}'$. At the equilibrium of the resident population,

$$\beta(K_i - I_i^*)I_i^* - (\mu+\alpha+\eta)I_i^* + \frac{1}{n}\sum_j \widetilde{m}_{ij}I_j^* - \frac{1}{n}\sum_j \widetilde{m}_{ji}I_i^* = 0, \tag{S3.24}$$

holds, from which the equilibrium local infected densities I_i^* are obtained. The equilibrium local infected densities are expanded in a Taylor series with respect to the magnitude ε of heterogeneity,

$$I_i^* = I_0^* + \varepsilon I_i^{*\prime} + \varepsilon^2 I_i^{*\prime\prime} + O(\varepsilon^3). \quad \text{[S3.25]}$$

The zeroth-order terms in the ε expansion of Eq. (S3.24) are collected as

$$\left[\beta(K_0 - I_0^*) - (\mu + \alpha + \eta)\right] I_0^* = 0, \quad \text{[S3.26]}$$

from which the equilibrium local density in homogeneous metapopulation is obtained,

$$I_0^* = K_0 - \frac{\mu + \alpha + \eta}{\beta} = K_0 \left(1 - \frac{1}{\mathcal{R}_0}\right), \quad \text{[S3.27]}$$

where $\mathcal{R}_0 = \beta K_0/(\mu + \alpha + \eta)$ is the basic reproduction ratio of the pathogen, which is assumed to be larger than 1. The equilibrium local susceptible density in a homogeneous metapopulation is $S_0^* = K_0 - I_0^* = K_0/\mathcal{R}_0$. The first-order terms in the ε expansion of Eq. (S3.24) are collected as

$$\beta I_0^* (K_i' - I_i^{*\prime}) + m_0 \left(\frac{1}{n} \sum_j I_j^{*\prime} - I_i^{*\prime} \right) + I_0^* T_i' = 0, \quad \text{[S3.28]}$$

from which the first-order deviation $I_i^{*\prime}$ for the equilibrium infected densities are obtained. Here, $T_i = \left(\sum_j \tilde{m}_{ij} - \sum_j \tilde{m}_{ji}\right)/n = \varepsilon T_i' = \varepsilon \left(\sum_j \tilde{m}_{ij}' - \sum_j \tilde{m}_{ji}'\right)/n$ is the net movement inflow to population i. We first note that, by summing up for all i of both sides of Eq. (S3.28), andnoting $\sum_i K_i' = \sum_i T_i' = 0$,

$$\beta I_0^* \sum_i I_i^{*\prime} = 0,$$

which leads to $\sum_i I_i^{*\prime} = 0$. Therefore, $I_i^{*\prime}$ and $S_i^{*\prime} = K_i' - I_i^{*\prime}$ are solved as

$$I_i^{*\prime} = \frac{\beta I_0^*}{m_0 + \beta I_0^*} K_i' + \frac{I_0^*}{m_0 + \beta I_0^*} T_i', \quad \text{[S3.29a]}$$

$$S_i^{*\prime} = \frac{m_0}{m_0 + \beta I_0^*} K_i' - \frac{I_0^*}{m_0 + \beta I_0^*} T_i'. \quad \text{[S3.29b]}$$

If we rewrite $S_i^{*\prime}$ defined in (S3.29b) as the sum of linear responses to environmental heterogeneities in a scaled form,

$$\frac{S_i^{*\prime}}{S_0^*} = q_K \frac{K_i'}{K_0} + q_T \frac{T_i'}{m_0}, \quad \text{[S3.30]}$$

we have

$$q_K = \frac{K_0}{S_0^*} \frac{m_0}{m_0 + \beta I_0^*} = \frac{\mathcal{R}_0}{1 + \frac{\beta K_0}{m_0}\left(1 - \frac{1}{\mathcal{R}_0}\right)}, \quad \text{[S3.31a]}$$

$$q_T = -\frac{m_0}{S_0^*} \frac{I_0^*}{m_0 + \beta I_0^*} = -\frac{\mathcal{R}_0 - 1}{1 + \frac{\beta K_0}{m_0}\left(1 - \frac{1}{\mathcal{R}_0}\right)}. \quad \text{[S3.31b]}$$

The elasticities of local susceptible densities to heterogeneities in carrying capacity and migration depend only on the basic reproduction ratio $\mathcal{R}_0$ of pathogen and the relative importance of transmission to that of migration, $\beta K_0/m_0$, in epidemiological dynamics. For a fixed basic reproduction ratio of the pathogen, the elasticities are high if changes by movement overwhelm those by transmission and low if otherwise. Looking at the signs of elasticities, we see that local susceptible densities are higher in populations with higher carrying capacities, and lower in populations with higher net movement inflow.

SIRS model with constant local host population densities As in the previous section, we derive the explicit forms of elasticities of local susceptible densities to heterogeneities by taking the limit of $r \to \infty$ so that a vacancy open by the mortality is again immediately filled by a newborn. Here, however, we do not assume $\gamma = 0$ so there are immune hosts as well as recoveries and immunity losses from them. Taking the limit of the ratios of F_S, F_I, and F_R, defined in Eq. (S3.8) to r_0 and noting that $N_0^* \to K_0$ as $r_0 \to \infty$, we see that they have the same limit -1,

$$\lim_{r_0\to\infty} \frac{F_S}{r_0} = \lim_{r_0\to\infty} \frac{F_I}{r_0} = \lim_{r_0\to\infty} \frac{F_R}{r_0} = -1.$$

The limit of the ratio of $|\mathbf{\Lambda_0}|$ to r_0 is

$$\begin{aligned}\lim_{r_0\to\infty} \frac{|\mathbf{\Lambda_0}|}{r_0} &= (m_0 + \beta I_0^*)(m_0 + \mu + \nu_0) + \gamma\beta I_0^* \\ &= m_0(m_0 + \mu + \nu_0) + \beta I_0^*(m_0 + \mu + \nu_0 + \gamma).\end{aligned}$$

In this limit, we have from Eq. (S3.3) that $N_0^* = K_0$, $S_0^* = (\mu + \alpha + \eta + \gamma)/\beta$, and $R_0^* = \gamma I_0^*/(\mu + \nu_0)$. This leads to

$$K_0 = \frac{K_0}{\mathcal{R}_0} + I_0^* + \frac{\gamma}{\mu + \nu_0} I_0^*$$

or

$$\begin{aligned} S_0^* &= \frac{K_0}{\mathcal{R}_0}, \\ I_0^* &= \frac{\mu + \nu}{\mu + \nu_0 + \gamma} K_0 \left(1 - \frac{1}{\mathcal{R}_0}\right), \\ R_0^* &= \frac{\gamma}{\mu + \nu_0 + \gamma} K_0 \left(1 - \frac{1}{\mathcal{R}_0}\right), \end{aligned}$$

where $\mathcal{R}_0 = \beta K_0/(\mu + \alpha + \eta + \gamma)$. From these, we have

$$\lim_{r_0\to\infty} \frac{|\mathbf{\Lambda_0}|}{r_0} = m_0(m_0 + \mu + \nu_0) \left[1 + \frac{\beta K_0}{m_0} \left(1 - \frac{1}{\mathcal{R}_0}\right) \frac{(\mu + \nu_0)(m_0 + \mu + \nu_0 + \gamma)}{(\mu + \nu_0 + \gamma)(m_0 + \mu + \nu_0)}\right]$$

and

$$\lim_{r_0\to\infty} q_T = -\frac{\mathcal{R}_0 - 1}{1 + \frac{\beta K_0}{m_0}\left(1 - \frac{1}{\mathcal{R}_0}\right)\omega}, \quad \text{[S3.32a]}$$

$$\lim_{r_0\to\infty} q_K = \frac{\mathcal{R}_0}{1 + \frac{\beta K_0}{m_0}\left(1 - \frac{1}{\mathcal{R}_0}\right)\omega}, \quad \text{[S3.32b]}$$

$$\lim_{r_0\to\infty} q_\nu = -\frac{\nu_0\gamma}{(m_0 + \mu + \nu_0)(\mu + \nu_0 + \gamma)} \frac{\mathcal{R}_0 - 1}{1 + \frac{\beta K_0}{m_0}\left(1 - \frac{1}{\mathcal{R}_0}\right)\omega}, \quad \text{[S3.32c]}$$

where

$$\omega = \frac{(\mu + \nu_0)(m_0 + \mu + \nu_0 + \gamma)}{(\mu + \nu_0 + \gamma)(m_0 + \mu + \nu_0)}. \quad \text{[S3.32d]}$$

We have the relationship $q_T = -(1 - 1/\mathcal{R}_0)q_K$ and $q_\nu = [\nu_0\gamma/(m_0 + \mu + \nu_0)(\mu + \nu_0 + \gamma)]q_T$ in the limit of $r_0 \to \infty$. The elasticities, q_K, q_T, and q_ν of the equilibrium local susceptible density to the local deviations of carrying capacity, net movement inflow, and immunity-loss rate are determined only by three dimensionless parameters, $\mathcal{R}_0$, $\beta K_0/m_0$, and $\omega = (\mu + \nu_0)(m_0 + \mu + \nu_0 + \gamma)/(\mu + \nu_0 + \gamma)(m_0 + \mu + \nu_0)$. From the sign of elasticities, we see that the local susceptible density is high in the populations where the host productivity is high, movement outflow dominates inflow (sources), and the immunity-loss rate is low. If $\mathcal{R}_0$ and ω are fixed, the elasticities are high when the changes by movement are faster than those by infection.

S4. Distribution for the sampling variation of ESS virulence in heterogeneous metapopulation

In this Appendix, we show that the relative increment of metapopulation ESS virulence under normally distributed local conditions is chi-square distributed with degree of freedom $n-1$, where n is the number of local populations. We will first show this for arbitrary n in the cases of metapopulation heterogeneities in birth rate, carrying capacity, and immunity loss rate. We then show that the same is true for movement heterogeneity for $n=2$ and $n=3$, and numerically agreed for n larger than 3.

S4.1. Heterogeneities in birth, carrying capacity, and immunity loss. If metapopulation heterogeneity is in the birth rate (r_i), carrying capacity (K_i), or immunity-loss rate of the host (ν_i), the increment of the ESS virulence, $\delta\alpha$, from that in a homogeneous metapopulation is proportional to the variance of heterogeneous parameter divided by the squared mean:

$$\frac{\delta\alpha^*}{\alpha_0^*} = \mathcal{E}_x \frac{\mathrm{Var}(x_i)}{E(x_i)^2}, \qquad \text{[S4.1]}$$

where $\mathcal{E}_x = \Theta q_x^2$ is the elasticity of ESS virulence to the heterogeneity in parameter x, where x is r, K, or ν (see Appendix S3 for the definition of Θ and q_x). The distribution for $\mathrm{Var}(x_i)/E(x_i)^2$ then gives the distribution for the increment of the ESS virulence in randomly assigned heterogeneity in a metapopulation with a finite number n of local populations. In this Appendix, we derive the distribution of the ESS virulence for randomly assigned metapopulation heterogeneity by looking at the distribution of $\mathrm{Var}(x_i)/E(x_i)^2$.

Let us explicitly describe how the random deviates, x_i's, with mean x_0 and the coefficient of variation ε are generated. We first generate n random deviates, ξ_i's ($i=1,\ldots,n$), by drawing n values independently from a common distribution with mean 0 and variance 1. The shape of the distribution can be arbitrary, but for simplicity, we assume here that it is normally distributed: $\xi_i \sim N(0,1)$. Our main result shown here, i.e., the scaled increment of ESS virulence is chi-square distributed with a degree of freedom $n-1$, remains approximately true by the central limit theorem for the arbitrary shape of the distribution for ξ_i's if the number n of populations is sufficiently large. Once a set of n random deviates, ξ_i's, are drawn, their sample mean $\bar{\xi} = \sum_j \xi_j/n$ is calculated. A new set of n random deviates is then defined from ξ_i's by subtracting their sample mean:

$$\zeta_i = \xi_i - \bar{\xi}, \qquad (i=1,\ldots,n), \qquad \text{[S4.2]}$$

which always have the sample mean 0: $\bar{\zeta} = 0$. A metapopulation heterogeneity in parameter x is then defined as

$$x_i = x_0 + \varepsilon x_i' = x_0(1+\varepsilon\zeta_i), \qquad (i=1,\ldots,n), \qquad \text{[S4.3]}$$

or $x_i' = x_0\zeta_i$. It then follows that the sample mean of x_i is always x_0 ($\bar{x} = \sum_i x_i/n = x_0$), and the sum of deviations from the metapopulation mean always vanishes ($\sum_i x_i' = 0$).

Then, recalling that ξ_i's are identically and independently distributed normal deviates with mean 0 and variance 1, $\sum_i (x_i'/x_0)^2$ follows a chi-square distribution with degree of freedom $n-1$:

$$\sum_i \left(\frac{x_i'}{x_0}\right)^2 = \sum_i (\zeta_i)^2 = \sum_i \left(\xi_i - \frac{1}{n}\sum_j \xi_j\right)^2 \sim \chi^2(n-1). \qquad \text{[S4.4]}$$

Noting that $\mathrm{Var}(x_i) = (1/n)\sum_i (x_i - x_0)^2 = \varepsilon^2 (1/n)\sum_i (x_i')^2$, we have $n\mathrm{Var}(x_i)/(\varepsilon x_0)^2 = \sum_i (x_i'/x_0)^2$. Therefore, from Eq. (S4.1),

$$\frac{\delta\alpha}{\alpha_0} = \mathcal{E}_x \frac{\mathrm{Var}(x_i)}{E(x_i)^2} = \frac{\varepsilon^2 \mathcal{E}_x}{n} \sum_i \left(\frac{x_i'}{x_0}\right)^2,$$

and hence the scaled ESS virulence increments, $Z = (\delta\alpha^*/\alpha_0^*)/(\varepsilon^2\mathcal{E}_x/n)$ is *chi-square distributed with degree of freedom* $n-1$:

$$Z = \frac{n}{\varepsilon^2 \mathcal{E}_x} \frac{\delta\alpha}{\alpha_0^*} = \sum_i \left(\frac{x_i'}{x_0}\right)^2 \sim \chi^2(n-1). \qquad \text{[S4.5]}$$

As Z has mean $n-1$ and variance $2(n-1)$, the mean and variance of $\delta\alpha^*/\alpha_0^*$ are

$$E\left(\frac{\delta\alpha^*}{\alpha_0^*}\right) = \frac{\varepsilon^2\mathscr{E}_x}{n}E(Z) = \varepsilon^2\mathscr{E}_x\left(\frac{n-1}{n}\right), \quad \text{[S4.6a]}$$

$$\mathrm{Var}\left(\frac{\delta\alpha^*}{\alpha_0^*}\right) = \left(\frac{\varepsilon^2\mathscr{E}_x}{n}\right)^2 \mathrm{Var}(Z) = \left(\varepsilon^2\mathscr{E}_x\right)^2\frac{2(n-1)}{n^2}, \quad \text{[S4.6b]}$$

and hence the coefficient of variation of $\delta\alpha/\alpha_0^*$ is

$$\mathrm{CV}\left(\frac{\delta\alpha^*}{\alpha_0^*}\right) = \frac{\sqrt{\mathrm{Var}(\delta\alpha^*/\alpha_0^*)}}{E(\delta\alpha^*/\alpha_0^*)} = \sqrt{\frac{2}{n-1}}. \quad \text{[S4.6c]}$$

S4.2. Heterogeneities in movement . A measure of the imbalance between incoming and outgoing movements of a population is measured by the net movement inflows:

$$T_i = \frac{1}{n}\left(\sum_{j\neq i}\widetilde{m}_{ij} - \sum_{j\neq i}\widetilde{m}_{ji}\right), \quad \text{[S4.7]}$$

and its total imbalance in a metapopulation is measured by the variance in T_i's:

$$V_T(n) = \mathrm{Var}(T_i) = \frac{1}{n}\sum_{i=1}^{n}(T_i)^2, \quad \text{[S4.8]}$$

where we note that $\sum_i T_i = 0$ and hence the mean of T_i's across metapopulation is 0. The heterogeneity in movement rates $\widetilde{m}_{ij}$ between populations affects the invasion fitness and the ESS virulence of pathogen only through the variance V_T if the magnitude of heterogeneity is sufficiently small. We also define the total movement imbalance of the metapopulation: $\mathscr{D}_n = nV_T(n) = \sum_i(T_i)^2$. Note that $\mathscr{D}_n' = \mathscr{D}_n/\varepsilon^2 = \sum_i(T_i')^2$.

The relative increment of ESS virulence in a metapopulation with movement heterogeneity is then expressed, as analogous to Eq. (S4.1)

$$\frac{\delta\alpha^*}{\alpha_0^*} = \mathscr{E}_T\frac{\mathrm{Var}(T_i)}{m_0^2} = \mathscr{E}_T\frac{1}{n}\sum_i\left(\frac{T_i}{m_0}\right)^2 = \mathscr{E}_T\varepsilon^2\frac{1}{n}\sum_i\left(\frac{T_i'}{m_0}\right)^2. \quad \text{[S4.9]}$$

Now we denote the movement rate from population j to i as

$$\widetilde{m}_{ij} = m_0 + \varepsilon\widetilde{m}_{ij}' = m_0(1+\varepsilon\xi_{ij}) \quad \text{[S4.10]}$$

or $\widetilde{m}_{ij}' = m_0\xi_{ij}$ and assume that ξ_{ij}'s are mutually independent normal deviations from mean 0 and variance 1: $\xi_{ij} \sim N(0,1)$. Then $\widetilde{m}_{ij}' \sim N(0, m_0^2)$ and $\widetilde{m}_{ij} \sim N(m_0, \varepsilon^2 m_0^2)$.

Two populations For the metapopulation with only two populations, $n = 2$,

$$\sum_{i=1}^{2}(T_i')^2 = (T_1')^2 + (T_2')^2 = 2(T_1')^2 = 2\left(\frac{1}{2}(m_{12}' - m_{21}')\right)^2 = m_0^2\left(\frac{\xi_{12}-\xi_{21}}{\sqrt{2}}\right)^2$$
$$\sim m_0^2\chi^2(1), \quad \text{[S4.11]}$$

or

$$\sum_{i=1}^{2}\left(\frac{T_i'}{m_0}\right)^2 \sim \chi^2(1)$$

where we used the fact that $T'_1 = (m'_{12} - m'_{21})/2 = -T'_2$, and ξ_{12} and that ξ_{21} are mutually independent standard normal variable, so that

$$\frac{\xi_{12} - \xi_{21}}{\sqrt{2}} \sim N(0,1).$$

Therefore

$$\frac{\delta\alpha}{\alpha_0^*} = \mathscr{E}_T \varepsilon^2 \frac{1}{2} \sum_{i=1}^{2} \left(\frac{T'_i}{m_0}\right)^2 \sim \frac{1}{2}\varepsilon^2 \mathscr{E}_T\, \chi^2(1). \qquad \text{[S4.12]}$$

In other words, the scaled increment of ESS virulence, $(\delta\alpha/\alpha_0^*)/(\varepsilon^2\mathscr{E}_T/2)$, for $n = 2$ is chi-square distributed with the degree of freedom 1.

Three populations If there are 3 populations ($n = 3$),

$$\begin{aligned}
\mathscr{D}'_3 = \sum_{i=1}^{3} (T'_i)^2 = \sum_{i=1}^{3} \left[\frac{1}{3}\left(\sum_{j=1}^{3} \widetilde{m}'_{ij} - \sum_{j=1}^{3} \widetilde{m}'_{ji}\right)\right]^2 &= \frac{2m_0^2}{9} \sum_{i=1}^{3} \left(\sum_{j=1}^{3} \frac{\xi_{ij} - \xi_{ji}}{\sqrt{2}}\right)^2 \\
&= \frac{2m_0^2}{9} \left[(\tau_{12} + \tau_{13})^2 + (\tau_{21} + \tau_{23})^2 + (\tau_{31} + \tau_{32})^2\right] \\
&= \frac{2m_0^2}{9} \left[(a-b)^2 + (b-c)^2 + (c-a)^2\right]. \qquad \text{[S4.13]}
\end{aligned}$$

where we set $\tau_{ij} = (\xi_{ij} - \xi_{ji})/\sqrt{2} = -\tau_{ji}$, $a = \tau_{12}$, $b = \tau_{23}$, and $c = \tau_{31}$. As ξ_{ij}'s are mutually independent, and normally distributed with mean 0 and variance 1, $a = \tau_{12} = (\xi_{12} - \xi_{21})/\sqrt{2}$ is normally distributed with mean 0 and variance 1: $a \sim N(0,1)$. Similarly, both b and c are normally distributed with mean 0 and variance 1, and a, b, and c are mutually independent.

Let us transform the coordinate system with bases $\boldsymbol{e_a} = (1,0,0)^\top$, $\boldsymbol{e_b} = (0,1,0)^\top$, $\boldsymbol{e_c} = (0,0,1)^\top$ to the system with a new orthogonal bases $\{\boldsymbol{e_x}, \boldsymbol{e_y}, \boldsymbol{e_z}\}$ including the vector $\boldsymbol{e_z} = (1,1,1)^\top/\sqrt{3}$:

$$\boldsymbol{e_x} = (-1,1,0)^\top/\sqrt{2}, \qquad \text{[S4.14a]}$$

$$\boldsymbol{e_y} = (-1,-1,2)^\top/\sqrt{6}, \qquad \text{[S4.14b]}$$

$$\boldsymbol{e_z} = (1,1,1)^\top/\sqrt{3}, \qquad \text{[S4.14c]}$$

with which the contour surface $\mathscr{D}'_3 = \sum_{i=1}^{3} (T'_i)^2 =$ const. is given by the surface of the cylinder centered at the axis $\boldsymbol{e_z}$ (we used the Gram-Schmidt algorithm to choose the rest of two base vectors $\boldsymbol{e_x}$ and $\boldsymbol{e_y}$). The coordinates x,y,z in this system are defined as

$$\boldsymbol{p} = (a,b,c)^\top = x\boldsymbol{e_x} + y\boldsymbol{e_y} + z\boldsymbol{e_z}, \qquad \text{[S4.15]}$$

or

$$a = -\frac{x}{\sqrt{2}} - \frac{y}{\sqrt{6}} + \frac{z}{\sqrt{3}}, \qquad \text{[S4.16a]}$$

$$b = \frac{x}{\sqrt{2}} - \frac{y}{\sqrt{6}} + \frac{z}{\sqrt{3}}, \qquad \text{[S4.16b]}$$

$$c = \frac{2y}{\sqrt{6}} + \frac{z}{\sqrt{3}}. \qquad \text{[S4.16c]}$$

The surface $\mathscr{D}'_3 = D'$ is expressed as

$$\begin{aligned}
\mathscr{D}'_3 &= \frac{2m_0^2}{9} \left[(a-b)^2 + (b-c)^2 + (c-a)^2\right] \\
&= \frac{2m_0^2}{9} \left[\left(-\frac{2x}{\sqrt{2}}\right)^2 + \left(\frac{x}{\sqrt{2}} - \frac{3y}{\sqrt{6}}\right)^2 + \left(\frac{x}{\sqrt{2}} + \frac{3y}{\sqrt{6}}\right)^2\right] \\
&= 2m_0^2 \frac{x^2 + y^2}{3} = D'. \qquad \text{[S4.17]}
\end{aligned}$$

The joint probability density function for a, b, and c is

$$\phi = C^3 \exp\left[-\frac{a^2+b^2+c^2}{2}\right] = C^3 \exp\left[-\frac{x^2+y^2+z^2}{2}\right] \qquad \text{[S4.18]}$$

with $C = 1/\sqrt{2\pi}$, because $a^2+b^2+c^2 = x^2+y^2+z^2$ follows as they represent the squared length of the same vector $\boldsymbol{p}$. Therefore, the probability distribution for $\mathscr{D}_3'$ is defined as

$$\begin{aligned} F(D') &= P(\mathscr{D}_3' < D') = P(x^2+y^2 < 3D'/2m_0^2) \\ &= \int\int\int_{x^2+y^2<3D'/2m_0^2} C^3 \exp\left[-\frac{x^2+y^2+z^2}{2}\right] \left|\frac{\partial(a,b,c)}{\partial(x,y,z)}\right| \mathrm{d}x\mathrm{d}y\mathrm{d}z \\ &= \int\int_{x^2+y^2<3D'/2m_0^2} C^2 \exp\left[-\frac{x^2+y^2}{2}\right] \mathrm{d}x\mathrm{d}y. \end{aligned} \qquad \text{[S4.19]}$$

From Eq. (S4.16), we see that the integration factor $|\partial(a,b,c)/\partial(x,y,z)| = 1$. By introducing the radial coordinates ($x = r\cos\theta, y = r\sin\theta$), the last integral is evaluated as

$$\begin{aligned} \int\int_{x^2+y^2<3D'/2m_0^2} C^2 \exp\left[-\frac{x^2+y^2}{2}\right] \mathrm{d}x\mathrm{d}y &= \int_0^{\sqrt{3D'/2m_0^2}} \int_0^{2\pi} \mathrm{d}\theta\, C^2 \exp\left[-\frac{r^2}{2}\right] \left|\frac{\partial(x,y)}{\partial(r,\theta)}\right| \mathrm{d}r \\ &= 2\pi\frac{1}{2\pi}\int_0^{\sqrt{3D'/2m_0^2}} \exp\left[-\frac{r^2}{2}\right] r\mathrm{d}r, \end{aligned} \qquad \text{[S4.20]}$$

and hence

$$F(D') = \int_0^{\sqrt{3D'/2m_0^2}} \exp\left[-\frac{r^2}{2}\right] r\mathrm{d}r. \qquad \text{[S4.21]}$$

The probability density function for $\mathscr{D}_3'$ is obtained by differentiating this with D':

$$\begin{aligned} f(D') = \frac{\mathrm{d}F(D')}{\mathrm{d}D'} &= \exp\left[-\frac{3D'}{4m_0^2}\right]\sqrt{\frac{3D'}{2m_0^2}}\frac{\mathrm{d}}{\mathrm{d}D'}\sqrt{\frac{3D'}{2m_0^2}} = \exp\left[-\frac{3D'}{4m_0^2}\right]\frac{3\sqrt{D'}}{2m_0^2}\frac{1}{2\sqrt{D'}} \\ &= \frac{3}{4m_0^2}\exp\left[-\frac{3D'}{4m_0^2}\right]. \end{aligned} \qquad \text{[S4.22]}$$

Therefore, $X = 3\mathscr{D}_3'/2m_0^2 = 3\sum_{i=1}^3 (T_i')^2/2m_0^2$ follows an exponential distribution with parameter 1/2 or the chi-square distribution with degree of freedom 2:

$$X = \frac{3\mathscr{D}_3'}{2m_0^2} = \frac{3}{2m_0^2}\sum_{i=1}^3 (T_i')^2 \sim \frac{1}{2}e^{-X/2} = \chi^2(2)$$

or

$$\frac{3}{2}\sum_{i=1}^3\left(\frac{T_i'}{m_0}\right)^2 \sim \chi^2(2),$$

and hence

$$\frac{\delta\alpha^*}{\alpha_0^*} = \mathscr{E}_T\varepsilon^2\frac{1}{3}\sum_{i=1}^3\left(\frac{T_i'}{m_0}\right)^2 \sim \frac{2}{9}\varepsilon^2\mathscr{E}_T\,\chi^2(2). \qquad \text{[S4.23]}$$

More than three populations These results, for $n = 2$ and $n = 3$, for the distribution of scaled increment of ESS virulence in metapopulation with heterogeneous movement rates are summarized as

$$\frac{\delta\alpha^*}{\alpha_0^*} = \mathcal{E}_T \varepsilon^2 \frac{1}{n} \sum_{i=1}^{n} \left(\frac{T_i'}{m_0}\right)^2 \sim \mathcal{E}_T \varepsilon^2 \frac{2}{n^2} \chi^2(n-1), \quad \text{[S4.24]}$$

which gives a conjecture that this holds for $n \geq 4$.

The scaled ESS virulence increments, $Z_n = (\delta\alpha^*/\alpha_0^*)/(2\varepsilon^2 \mathcal{E}_T/n^2)$ is chi-square distributed with degree of freedom $n-1$:

$$Z_n = \frac{n^2}{2\varepsilon^2 \mathcal{E}_T} \frac{\delta\alpha}{\alpha_0^*} = \frac{n}{2} \sum_{i=1}^{n} \left(\frac{T_i'}{m_0}\right)^2 \sim \chi^2(n-1). \quad \text{[S4.25]}$$

As Z_n has mean $n-1$ and variance $2(n-1)$, the mean and variance of $\delta\alpha^*/\alpha_0^*$ are

$$E\left(\frac{\delta\alpha^*}{\alpha_0^*}\right) = \frac{2\varepsilon^2 \mathcal{E}_T}{n^2} E(Z_n) = 2\varepsilon^2 \mathcal{E}_T \left(\frac{n-1}{n^2}\right), \quad \text{[S4.26a]}$$

$$\mathrm{Var}\left(\frac{\delta\alpha^*}{\alpha_0^*}\right) = \left(\frac{2\varepsilon^2 \mathcal{E}_T}{n^2}\right)^2 \mathrm{Var}(Z_n) = \left(2\varepsilon^2 \mathcal{E}_T\right)^2 \frac{2(n-1)}{n^4}, \quad \text{[S4.26b]}$$

and hence the coefficient of variation of $\delta\alpha/\alpha_0^*$ is

$$\mathrm{CV}\left(\frac{\delta\alpha^*}{\alpha_0^*}\right) = \frac{\sqrt{\mathrm{Var}(\delta\alpha^*/\alpha_0^*)}}{E(\delta\alpha^*/\alpha_0^*)} = \sqrt{\frac{2}{n-1}}. \quad \text{[S4.26c]}$$

Though Eqn. (S4.26a) and (S4.26b) are factor $n/2$ different from Eqn. (S4.6a) and (S4.6b), the coefficient of variation Eqn. (S4.26c) is exactly the same as (S4.6c) if we replace $\mathcal{E}_T$ with $\mathcal{E}_x$.

See Figure S4 for the distributions of $Z_n = (\delta\alpha/\alpha_0^*)/[2\mathcal{E}_T \varepsilon^2/n^2]$ in randomly generated movement patterns with $n = 2$, 3, 20, and 100. Though we cannot show the proof for Eq. (S4.24) for general n, the distributions of Z_n fitted well to $\chi^2(n-1)$, not only for $n = 2, 3$, but for larger n as well (Fig. S4).

S5. Higher-order predictions in heterogeneous metapopulations

In our previous analysis for ESS virulence in heterogeneous metapopulation in Appendix S2, we assumed that the degree ε of heterogeneity is sufficiently small so that we can ignore the third or higher-order deviations in the selection gradient to predict the ESS virulence. As Figure 3 in the main text shows, our qualitative result that heterogeneity always increases ESS virulence remains true even when the degree of heterogeneity is large. However, we found a non-negligible departure from a linear dependency on the squared coefficient of variation when the degree of heterogeneity becomes moderately large. Here we derive more accurate predictions for a large degree of heterogeneity by taking into account the third and fourth-order terms in the selection gradient.

In S5.1, we first describe an epidemiological model in heterogeneous metapopulation where we analyze the nonlinear dependence of ESS virulence and infectivity of the pathogen on local conditions. In S5.2, we then derive the equilibrium densities of infected hosts in a heterogeneous metapopulation with small variations in local carrying capacities up to the third-order terms of Taylor expansion with respect to the degree ε of local variations.

S5.1. SIS model with constant local host population densities. Our analysis here is restricted to the simplest epidemiological dynamics we dealt with in the present paper: the SIS model where the local population sizes are always kept constant at their carrying capacity (with sufficiently fast host demographic change): $S_i + I_i = K_i$ $(i = 1, \ldots, n)$. We assumed that the carrying capacity varies around its mean, K_0, as

$$K_i = K_0 + \varepsilon K_i'. \quad \text{[S5.1]}$$

We further assume that the metapopulation heterogeneity is only in carrying capacities so that movement between populations is isotropic.

Under these simplified assumptions, epidemiological dynamics for the number of the infected hosts, I_i, in population i is, noting that $S_i = K_i - I_i = K_0 + \varepsilon K'_i - I_i$ gives the susceptible host density at population i,

$$\frac{\mathrm{d}I_i}{\mathrm{d}t} = \beta(K_0 + \varepsilon K'_i - I_i)I_i - vI_i + \frac{m_0}{n}\sum_j (I_j - I_i), \tag{S5.2}$$

where $v = \mu + \alpha + \eta$. Note that $\sum_i K'_i = 0$ by definition.

For notational simplicity, we introduce the vector of the scaled density of equilibrium infected densities, $\boldsymbol{y} = (y_1, y_2, \cdots, y_n)^\top$, as well as the vector of the scaled carrying capacities, $\boldsymbol{k} = (k_1, k_2, \cdots, k_n)^\top$ and the scaled transmission rate b:

$$\boldsymbol{y} = \frac{\boldsymbol{I}^*}{K_0}, \tag{S5.3a}$$

$$\boldsymbol{k} = \frac{\boldsymbol{K}}{K_0}, \tag{S5.3b}$$

$$b = \beta K_0, \tag{S5.3c}$$

where $\boldsymbol{y_0} = y_0\mathbf{1} = y_0(1, 1, \cdots, 1)^\top$ and $\boldsymbol{k'} = \boldsymbol{K'}/K_0 = (k'_1, k'_2, \cdots, k'_n)^\top$. The vector of equilibrium densities of susceptible hosts is also rescaled as $\boldsymbol{x} = \boldsymbol{S}^*/K_0$.

S5.2. Expansion of equilibrium densities . The equilibrium condition for Eq. (S5.2) in scaled form:

$$(b(\boldsymbol{k} - \boldsymbol{y}) - v) \circ \boldsymbol{y} + m_0\left(\frac{\mathbf{1}^\top \boldsymbol{y}}{n}\mathbf{1} - \boldsymbol{y}\right) = \mathbf{0}, \tag{S5.4}$$

where $\boldsymbol{a} \circ \boldsymbol{b}$ indicates taking the element-wise product of vectors $\boldsymbol{a}$ and $\boldsymbol{b}$: e.g., $\boldsymbol{a} \circ \boldsymbol{b} = (a_1b_1, \ldots, a_nb_n)^\top$, and $\boldsymbol{a} \circ \boldsymbol{b} \circ \boldsymbol{c} = (a_1b_1c_1, \ldots, a_nb_nc_n)^\top$. Here, $\mathbf{1} = (1, \ldots, 1)^\top$ is the vector of 1's.

We now expand the scaled density of infected hosts $\boldsymbol{y}$ in a Talyor series with respect to the degree ε of the metapopulation heterogeneity in carrying capacity:

$$\boldsymbol{y} = \boldsymbol{y_0} + \varepsilon\boldsymbol{y'} + \varepsilon^2\boldsymbol{y''} + \varepsilon^3\boldsymbol{y'''} + O(\varepsilon^4). \tag{S5.5}$$

We then substitute Eq. (S5.5) and

$$\boldsymbol{k} = \mathbf{1} + \varepsilon\boldsymbol{k'} \tag{S5.6}$$

into Eq. (S5.4), and collecting all terms of the same order of the power of ε polynomial to solve zeroth, first, second, and third order deviations of the scaled equilibrium density of infected hosts:

$$\boldsymbol{y_0} = y_0\mathbf{1} = \frac{1}{\vartheta}\mathbf{1}, \tag{S5.7a}$$

$$\boldsymbol{y'} = \chi\boldsymbol{k'}, \tag{S5.7b}$$

$$\boldsymbol{y''} = \vartheta\chi^2(1-\chi)\left(\boldsymbol{k'} \circ \boldsymbol{k'} + \frac{1-\chi}{\chi}V_k\mathbf{1}\right), \tag{S5.7c}$$

$$\boldsymbol{y'''} = \vartheta^2\chi^3(1-\chi)(1-2\chi)\left(\boldsymbol{k'} \circ \boldsymbol{k'} \circ \boldsymbol{k'} + \frac{1-\chi}{\chi}\left(T_k\mathbf{1} + V_k\boldsymbol{k'}\right)\right). \tag{S5.7d}$$

Here,

$$\boldsymbol{k'} = (k'_1, \ldots, k'_n)^\top, \tag{S5.8a}$$

$$\boldsymbol{k'} \circ \boldsymbol{k'} = ({k'_1}^2, \ldots, {k'_n}^2)^\top, \tag{S5.8b}$$

$$\boldsymbol{k'} \circ \boldsymbol{k'} \circ \boldsymbol{k'} = ({k'_1}^3, \ldots, {k'_n}^3)^\top, \tag{S5.8c}$$

are the vectors of the local variations, squared local variations, and cubed local variations of scaled carrying capacities from the metapopulation average, and

$$V_k = \frac{1}{n}\sum_i {k'_i}^2 = \frac{\mathbf{1}^\top(\boldsymbol{k}' \circ \boldsymbol{k}')}{n}, \tag{S5.9a}$$

$$T_k = \frac{1}{n}\sum_i {k'_i}^3 = \frac{\mathbf{1}^\top(\boldsymbol{k}' \circ \boldsymbol{k}' \circ \boldsymbol{k}')}{n}, \tag{S5.9b}$$

$$Q_k = \frac{1}{n}\sum_i {k'_i}^4 = \frac{\mathbf{1}^\top(\boldsymbol{k}' \circ \boldsymbol{k}' \circ \boldsymbol{k}' \circ \boldsymbol{k}')}{n}, \tag{S5.9c}$$

are the variance, third and fourth central moments of the scaled (by K_0) carrying capacities further scaled by ε^2, ε^3, and ε^4, respectively (i.e., $\varepsilon^2 V_k$, $\varepsilon^3 T_k$, and $\varepsilon^4 Q_k$ respectively give the actual values of variance, third and fourth central moments of the carrying capacity scaled by K_0). Finally, the parameters χ and ϑ are defined as

$$\chi = \frac{b-v}{b-v+m_0} = \frac{by_0}{by_0+m_0}, \tag{S5.10a}$$

$$\vartheta = \frac{b}{b-v} = \frac{1}{y_0}, \tag{S5.10b}$$

where we used the endemic equilibrium condition, $\beta S_0^* - v = bx_0 - v = b(1-y_0) - v = 0$ or $b - v = by_0$, in homogeneous population.

Derivation of zeroth, first, second, and third order deviations of the scaled equilibrium infected densities. Now we derive the expressions Eq. (S5.7) of the zeroth, first, second, and third order deviations of the scaled equilibrium infected densities, $\boldsymbol{y_0}$, $\boldsymbol{y}'$, $\boldsymbol{y}''$, and $\boldsymbol{y}'''$. We first substitute Eq. (S5.5) and Eq. (S5.6) into Eq. (S5.4):

$$\begin{aligned}\mathbf{0} = &\Big[\{b(\mathbf{1}-\boldsymbol{y_0}) - v\} + \varepsilon b(\boldsymbol{k}' - \boldsymbol{y}') - \varepsilon^2 b\boldsymbol{y}'' - \varepsilon^3 b\boldsymbol{y}''' + O(\varepsilon^4)\Big] \circ (\boldsymbol{y_0} + \varepsilon\boldsymbol{y}' + \varepsilon^2\boldsymbol{y}'' + \varepsilon^3\boldsymbol{y}''' + O(\varepsilon^4)) \\ &+ m_0\frac{\mathbf{1}^\top(\boldsymbol{y_0} + \varepsilon\boldsymbol{y}' + \varepsilon^2\boldsymbol{y}'' + \varepsilon^3\boldsymbol{y}''' + O(\varepsilon^4))}{n}\mathbf{1} - m_0(\boldsymbol{y_0} + \varepsilon\boldsymbol{y}' + \varepsilon^2\boldsymbol{y}'' + \varepsilon^3\boldsymbol{y}''' + O(\varepsilon^4)).\end{aligned} \tag{S5.11}$$

Zeroth-order term Collecting terms of the zeroth-power of ε in Eq. (S5.11), we have $(b(1-y_0) - v)y_0 = 0$, from which the endemic equilibrium density of infected hosts in the isotropic case ($\varepsilon = 0$):

$$y_0 = 1 - v/b = 1/\vartheta. \tag{S5.12}$$

This completes the derivation of Eq. (S5.7a). ■

First-order term Collecting terms of the first-power of ε in Eq. (S5.11), by noting that $\boldsymbol{a} \circ \mathbf{1} = \boldsymbol{a}$ for arbitrary vector $\boldsymbol{a}$ and that $b(1-y_0) - v = 0$ from Eq. (S5.12),

$$b(\boldsymbol{k}' - \boldsymbol{y}')y_0 + m_0\frac{\mathbf{1}^\top\boldsymbol{y}'}{n}\mathbf{1} - m_0\boldsymbol{y}' = \mathbf{0}. \tag{S5.13}$$

Multiplying $\mathbf{1}^\top$ from left to both sides of Eq. (S5.13), and noting $\mathbf{1}^\top\mathbf{1} = n$ and $\mathbf{1}^\top\boldsymbol{k}' = \sum_i k'_i = 0$, we see that

$$\mathbf{1}^\top\boldsymbol{y}' = \sum_i y'_i = 0.$$

Substituting this into Eq. (S5.13),

$$\boldsymbol{y}' = \frac{by_0}{by_0+m_0}\boldsymbol{k}' = \chi\boldsymbol{k}'. \tag{S5.14}$$

where χ is defined in Eq. (S5.10a). This completes the derivation of Eq. (S5.7b). ■

Second-order term Collecting terms of the second-power of ε in Eq. (S5.11),

$$(b(1-y_0)-v)\boldsymbol{y}''+b(\boldsymbol{k}'-\boldsymbol{y}')\circ\boldsymbol{y}'-b\boldsymbol{y}''y_0+m_0\frac{\mathbf{1}^\top\boldsymbol{y}''}{n}\mathbf{1}-m_0\boldsymbol{y}''=\mathbf{0}.$$

As $b(1-y_0)-v=0$,

$$b(\boldsymbol{k}'-\boldsymbol{y}')\circ\boldsymbol{y}'-(by_0+m_0)\boldsymbol{y}''+m_0\frac{\mathbf{1}^\top\boldsymbol{y}''}{n}\mathbf{1}=\mathbf{0}. \qquad \text{[S5.15]}$$

Multiplying $\mathbf{1}^\top$ from left to both sides of Eq. (S5.15) and noting $\mathbf{1}^\top\mathbf{1}=n$, we see that terms proportional to m_0 vanish, and

$$b\mathbf{1}^\top(\boldsymbol{k}'-\boldsymbol{y}')\circ\boldsymbol{y}'-by_0\mathbf{1}^\top\boldsymbol{y}''=\mathbf{0}.$$

Solving this for $\mathbf{1}^\top\boldsymbol{y}''$, by noting $\boldsymbol{y}'=\chi\boldsymbol{k}'$ and $1/y_0=\vartheta$, we have

$$\begin{aligned}\frac{\mathbf{1}^\top\boldsymbol{y}''}{n}&=\frac{1}{y_0}\frac{\mathbf{1}^\top((\boldsymbol{k}'-\boldsymbol{y}')\circ\boldsymbol{y}')}{n}\\&=\vartheta\chi(1-\chi)\frac{\mathbf{1}^\top(\boldsymbol{k}'\circ\boldsymbol{k}')}{n}\\&=\vartheta\chi(1-\chi)V_k.\end{aligned} \qquad \text{[S5.16]}$$

See Eq. (S5.9) for the definition of V_k. Substituting this and Eq. (S5.7b) into Eq. (S5.15), we have

$$\begin{aligned}\boldsymbol{y}''&=\frac{1}{by_0+m_0}\left(b\chi(1-\chi)\boldsymbol{k}'\circ\boldsymbol{k}'+m_0\vartheta\chi(1-\chi)V_k\mathbf{1}\right)\\&=\frac{1}{y_0}\frac{by_0}{by_0+m_0}\chi(1-\chi)\left(\boldsymbol{k}'\circ\boldsymbol{k}'+\frac{m_0}{b}\vartheta V_k\mathbf{1}\right)\\&=\vartheta\chi^2(1-\chi)\left(\boldsymbol{k}'\circ\boldsymbol{k}'+\frac{1-\chi}{\chi}V_k\mathbf{1}\right),\end{aligned} \qquad \text{[S5.17]}$$

where we used $by_0/(by_0+m_0)=\chi$, $1/y_0=\vartheta$, and $b/m_0=\vartheta\chi/(1-\chi)$ that follow from the definitions Eq. (S5.10a) and Eq. (S5.10b) of χ and ϑ. This completes the derivation of Eq. (S5.7c). ■

Third-order term Collecting terms of the third-power of ε in Eq. (S5.11),

$$\begin{aligned}\mathbf{0}&=(b(1-y_0)-v)\boldsymbol{y}'''+b(\boldsymbol{k}'-\boldsymbol{y}')\circ\boldsymbol{y}''-b\boldsymbol{y}''\circ\boldsymbol{y}'-b\boldsymbol{y}'''y_0\\&\quad+m_0\frac{\mathbf{1}^\top\boldsymbol{y}'''}{n}\mathbf{1}-m_0\boldsymbol{y}'''\\&=b(\boldsymbol{k}'-2\boldsymbol{y}')\circ\boldsymbol{y}''-(by_0+m_0)\boldsymbol{y}'''+m_0\frac{\mathbf{1}^\top\boldsymbol{y}'''}{n}\mathbf{1}.\end{aligned} \qquad \text{[S5.18]}$$

Multiplying $\mathbf{1}^\top$ from left to both sides of Eq. (S5.18), and noting $\mathbf{1}^\top\mathbf{1}=n$, we have

$$b\mathbf{1}^\top(\boldsymbol{k}'-2\boldsymbol{y}')\circ\boldsymbol{y}''-by_0\mathbf{1}^\top\boldsymbol{y}'''=0.$$

Solving this for $\mathbf{1}^\top\boldsymbol{y}'''$ by noting $\mathbf{1}^\top(\boldsymbol{k}'-2\boldsymbol{y}')\circ\boldsymbol{y}''=(1-2\chi)\mathbf{1}^\top(\boldsymbol{k}'\circ\boldsymbol{y}'')$ and $1/y_0=\vartheta$,

$$\frac{\mathbf{1}^\top\boldsymbol{y}'''}{n}=\vartheta(1-2\chi)\frac{1}{n}\mathbf{1}^\top(\boldsymbol{y}''\circ\boldsymbol{k}') \qquad \text{[S5.19]}$$

Substituting Eq. (S5.7c),

$$\begin{aligned}\frac{\mathbf{1}^\top\boldsymbol{y}'''}{n}&=\vartheta(1-2\chi)\frac{1}{n}\mathbf{1}^\top\left[\vartheta\chi^2(1-\chi)\left(\boldsymbol{k}'\circ\boldsymbol{k}'+\frac{1-\chi}{\chi}V_k\mathbf{1}\right)\circ\boldsymbol{k}'\right]\\&=\vartheta^2\chi^2(1-\chi)(1-2\chi)\frac{\mathbf{1}^\top(\boldsymbol{k}'\circ\boldsymbol{k}'\circ\boldsymbol{k}')}{n}\\&=\vartheta^2\chi^2(1-\chi)(1-2\chi)T_k,\end{aligned} \qquad \text{[S5.20]}$$

where we used $\mathbf{1}^\top \boldsymbol{k'} = 0$. See Eq. (S5.9) for the definition of T_k. Substituting this, Eq. (S5.7b), and Eq. (S5.7c) into Eq. (S5.18), $\boldsymbol{y'''}$ is obtained as

$$\begin{aligned}
\boldsymbol{y'''} &= \frac{1}{y_0}\frac{by_0}{by_0+m_0}(1-2\chi)\boldsymbol{k'}\circ\boldsymbol{y''} + \frac{m_0}{by_0}\frac{by_0}{by_0+m_0}\frac{\mathbf{1}^\top\boldsymbol{y'''}}{n}\mathbf{1} \\
&= \vartheta\chi(1-2\chi)\boldsymbol{k'}\circ\left[\vartheta\chi^2(1-\chi)\left(\boldsymbol{k'}\circ\boldsymbol{k'}+\frac{1-\chi}{\chi}V_k\mathbf{1}\right)\right] + \frac{1-\chi}{\chi}\chi\vartheta^2\chi^2(1-\chi)(1-2\chi)T_k\mathbf{1} \\
&= \vartheta^2\chi^3(1-\chi)(1-2\chi)\left[(\boldsymbol{k'}\circ\boldsymbol{k'}\circ\boldsymbol{k'})+\frac{1-\chi}{\chi}\left(V_k\boldsymbol{k'}+T_k\mathbf{1}\right)\right]. \qquad \text{[S5.21]}
\end{aligned}$$

This completes the derivation of Eq. (S5.7d). ■

S5.3. Eigenvectors of Jacobian for invasion of neutral pathogen variants. In the previous subsection S5.2, we have derived the approximate equilibrium densities in the metapopulation up to the third-order terms of the degree ε of heterogeneity. To derive the approximate selection gradient up to the fourth-order terms of ε, we also need to obtain the eigenvectors of the Jacobian for the invasion of a pathogen variant that has an identical trait as the resident for the necessary degree of precision, up to the third order of ε, which we will do in this subsection.

To do this, in this subsection S5.3, we first expand the Jacobian $\boldsymbol{J}$ for the invasion of a mutant that has identical trait as the resident, up to the fourth-order terms of ε. Then, in S5.4 and S5.5, we derive the left and right eigenvectors of $\boldsymbol{G} = \boldsymbol{J}/m_0$ up to the third order of ε.

Expansion of Jacobian We consider the invasibility of a new pathogen variant that has a virulence $\hat{\alpha}$ that differs from that α of the resident. Assuming that the resident metapopulation is in endemic equilibrium with the scaled equilibrium infected density vector $\boldsymbol{y} = (y_1, y_2, \cdots, y_n)^\top = \boldsymbol{I}^*/K_0$ obtained in S5.2, the dynamics of the scaled densities of hosts infected by the new variant, $\boldsymbol{z} = (z_1, z_2, \cdots, z_n)^\top = (\hat{I}_1, \ldots, \hat{I}_n)^\top/K_0$ changes with time when rare as

$$\frac{\mathrm{d}\hat{z}_i}{\mathrm{d}t} = \hat{b}(k_i - y_i)z_i - \hat{v}z_i + m_0\left(\frac{1}{n}\sum\nolimits_j z_j - z_i\right), \qquad \text{[S5.22]}$$

where $\boldsymbol{x} = \boldsymbol{k} - \boldsymbol{y} = (k_1 - y_1, k_2 - y_2, \cdots, k_n - y_n)^\top$ is the scaled equilibrium density of susceptible hosts in the resident population, $\hat{v} = \mu + \hat{\alpha} + \eta$, is the rate of loss of new-variant-infected hosts by natural mortality, disease-induced mortality, and recovery, and $\hat{b} = \beta(\hat{\alpha})K_0$ is the scaled transmission rate of a new variant under the tradeoff $\beta = \beta(\alpha)$. In vector form for $\boldsymbol{z} = (\hat{I}_1, \ldots, \hat{I}_n)^\top/K_0$, this is rewritten as

$$\begin{aligned}
\frac{\mathrm{d}\boldsymbol{z}}{\mathrm{d}t} &= \begin{pmatrix} \hat{b}(k_1-y_1)-\hat{v} & 0 & \cdots & 0 \\ 0 & \hat{b}(k_2-y_2)-\hat{v} & \ddots & \vdots \\ \vdots & \ddots & \ddots & 0 \\ 0 & \cdots & 0 & \hat{b}(k_n-y_n)-\hat{v} \end{pmatrix}\boldsymbol{z} + m_0\left(\frac{1}{n}\begin{pmatrix}1 & \cdots & 1\\ \vdots & \ddots & \vdots \\ 1 & \cdots & 1\end{pmatrix} - \boldsymbol{I}\right)\boldsymbol{z} \\
&= \hat{\boldsymbol{J}}\boldsymbol{z}, \qquad \text{[S5.23]}
\end{aligned}$$

where $\boldsymbol{I}$ is n dimensional identity matrix and $\hat{\boldsymbol{J}}$ is the Jacobian for the invasion dynamics of a new variant.

We focus on the case where the new variant has identical virulence and infectiousness as the resident: $\hat{\alpha} = \alpha$ and hence $\hat{\beta} = \beta$. The Jacobian $\boldsymbol{J} = \hat{\boldsymbol{J}}\Big|_{\hat{\alpha}=\alpha}$ for the invasion dynamics Eq. (S5.23) evaluated at $\hat{\alpha} = \alpha$ is

$$\boldsymbol{J} = \begin{pmatrix} b(k_1-y_1)-v & 0 & \cdots & 0 \\ 0 & b(k_2-y_2)-v & \ddots & \vdots \\ \vdots & \ddots & \ddots & 0 \\ 0 & \cdots & 0 & b(k_n-y_n)-v \end{pmatrix} + m_0\left(\frac{1}{n}\begin{pmatrix}1 & \cdots & 1\\ \vdots & \ddots & \vdots \\ 1 & \cdots & 1\end{pmatrix} - \boldsymbol{I}\right). \qquad \text{[S5.24]}$$

We expand the Jacobian $\boldsymbol{J}$ in a Taylor series with respect to ε as

$$\boldsymbol{J} = \boldsymbol{J_0} + \varepsilon \boldsymbol{J'} + \varepsilon^2 \boldsymbol{J''} + \varepsilon^3 \boldsymbol{J'''} + O(\varepsilon^4),$$

by substituting $\boldsymbol{y} = y_0 \mathbf{1} + \varepsilon \boldsymbol{y'} + \varepsilon^2 \boldsymbol{y''} + \varepsilon^3 \boldsymbol{y'''} + O(\varepsilon^4)$ and $\boldsymbol{k} = \mathbf{1} + \varepsilon \boldsymbol{k'}$ into Eq. (S5.24). Noting that $b(1 - y_0) - v = 0$, we have

$$\boldsymbol{J_0} = m_0 \left(\frac{1}{n} \begin{pmatrix} 1 & \cdots & 1 \\ \vdots & \ddots & \vdots \\ 1 & \cdots & 1 \end{pmatrix} - \boldsymbol{I} \right), \quad \text{[S5.25a]}$$

$$\boldsymbol{J'} = b\boldsymbol{\Sigma'} = b(\mathcal{K}' - \mathcal{Y}') = b \begin{pmatrix} k'_1 - y'_1 & 0 & \cdots & 0 \\ 0 & k'_2 - y'_2 & \ddots & \vdots \\ \vdots & \ddots & \ddots & 0 \\ 0 & \cdots & 0 & k'_n - y'_n \end{pmatrix}, \quad \text{[S5.25b]}$$

$$\boldsymbol{J''} = b\boldsymbol{\Sigma''} = -b\mathcal{Y}'' = -b \begin{pmatrix} y''_1 & 0 & \cdots & 0 \\ 0 & y''_2 & \ddots & \vdots \\ \vdots & \ddots & \ddots & 0 \\ 0 & \cdots & 0 & y''_n \end{pmatrix}, \quad \text{[S5.25c]}$$

$$\boldsymbol{J'''} = b\boldsymbol{\Sigma'''} = -b\mathcal{Y}''' = -b \begin{pmatrix} y'''_1 & 0 & \cdots & 0 \\ 0 & y'''_2 & \ddots & \vdots \\ \vdots & \ddots & \ddots & 0 \\ 0 & \cdots & 0 & y'''_n \end{pmatrix}, \quad \text{[S5.25d]}$$

where $\boldsymbol{\Sigma} = \mathrm{diag}(x_1, x_2, \cdots, x_n)$, $\mathcal{K} = \mathrm{diag}(k_1, k_2, \cdots, k_n)$, and $\mathcal{Y} = \mathrm{diag}(y_1, y_2, \cdots, y_n)$, are the identity matrices whose diagonal elements are the scaled equilibrium susceptible densities, the scaled carrying capacities, and the scaled equilibrium infected densities in local populations: $\boldsymbol{\Sigma} = \mathcal{K} - \mathcal{Y}$. As defined in S5.2, $\boldsymbol{k} = \mathbf{1} + \varepsilon \boldsymbol{k'}$ represents the scaled carrying capacities varied around their metapopulation average. The corresponding equilibrium infected densities $\boldsymbol{y} = \boldsymbol{y_0} + \varepsilon \boldsymbol{y'} + \cdots$ under such heterogeneity in carrying capacities are obtained in S5.2 up to the third order deviations with respect to the degree ε of heterogeneity.

Expansion of left and right eigenvectors In this subsection, we derive the left and right eigenvectors of the matrix $\boldsymbol{G} = \boldsymbol{J}/m_0$ up to the third order of ε, the degree of metapopulation heterogeneity in carrying capacities.

As the new pathogen variant has identical virulence and infectivity to the resident pathogen, the dominant eigenvector of the Jacobian $\boldsymbol{J}$, or the invasion fitness, of the new variant should be zero. Therefore, the corresponding left and right eigenvectors, $\boldsymbol{v}$ and $\boldsymbol{\phi}$, of the matrix $\boldsymbol{G} = \boldsymbol{J}/m_0$ satisfy the eigenequations

$$\boldsymbol{G\phi} = \mathbf{0}, \quad \text{[S5.26a]}$$

$$\boldsymbol{v}^\top \boldsymbol{G} = \mathbf{0}^\top. \quad \text{[S5.26b]}$$

where, as before, $^\top$ denotes the transpose.

We then expand the dominant left and right eigenvectors, $\boldsymbol{v}$ and $\boldsymbol{\phi}$, of the matrix $\boldsymbol{G}$ in a Taylor series with respect to the degree ε of metapopulation heterogeneity:

$$\boldsymbol{\phi} = \boldsymbol{\phi_0} + \varepsilon \boldsymbol{\phi'} + \varepsilon^2 \boldsymbol{\phi''} + \ldots. \quad \text{[S5.27a]}$$

$$\boldsymbol{v} = \boldsymbol{v_0} + \varepsilon \boldsymbol{v'} + \varepsilon^2 \boldsymbol{v''} + \ldots. \quad \text{[S5.27b]}$$

S5.4. Expansion of dominant right eigenvector. Here, we show that each term of ε-expansion of the right eigenvector $\boldsymbol{\phi} = \boldsymbol{\phi_0} + \varepsilon\boldsymbol{\phi}' + \varepsilon^2\boldsymbol{\phi}'' + \varepsilon^3\boldsymbol{\phi}''' + O(\varepsilon^4)$ up to the third-order is

$$\boldsymbol{\phi_0} = \frac{\boldsymbol{1}}{n}, \quad \text{[S5.28a]}$$

$$\boldsymbol{\phi}' = \frac{1}{n}\vartheta\chi\boldsymbol{k}', \quad \text{[S5.28b]}$$

$$\boldsymbol{\phi}'' = \frac{1}{n}\vartheta^2\chi^2(1-\chi)\left(\boldsymbol{k}'\circ\boldsymbol{k}' - V_k\boldsymbol{1}\right), \quad \text{[S5.28c]}$$

$$\boldsymbol{\phi}''' = \frac{1}{n}\vartheta^3\chi^3(1-\chi)\left[(1-2\chi)\left(\boldsymbol{k}'\circ\boldsymbol{k}'\circ\boldsymbol{k}' - T_k\boldsymbol{1}\right) - (3-2\chi)V_k\boldsymbol{k}'\right]. \quad \text{[S5.28d]}$$

In the rest of this subsection, we derive Eq. (S5.28a)–Eq. (S5.28d) step by step.

Substituting Eq. (S5.27) into Eq. (S5.26), denoting $\boldsymbol{G_0} = \boldsymbol{J_0}/m_0$, $\boldsymbol{G}' = \boldsymbol{J}'/m_0$ and so on, the eigenequation corresponding the dominant eigenvalue 0 is

$$(\boldsymbol{G_0} + \varepsilon\boldsymbol{G}' + \ldots)(\boldsymbol{\phi_0} + \varepsilon\boldsymbol{\phi}' + \ldots) = \boldsymbol{0}.$$

Equating the terms in the same order of ε powers, we have

$$\boldsymbol{G_0}\boldsymbol{\phi_0} = \boldsymbol{0}, \quad \text{[S5.29a]}$$

$$\boldsymbol{G_0}\boldsymbol{\phi}' + \boldsymbol{G}'\boldsymbol{\phi_0} = \boldsymbol{0}, \quad \text{[S5.29b]}$$

$$\boldsymbol{G_0}\boldsymbol{\phi}'' + \boldsymbol{G}'\boldsymbol{\phi}' + \boldsymbol{G}''\boldsymbol{\phi_0} = \boldsymbol{0}, \quad \text{[S5.29c]}$$

$$\boldsymbol{G_0}\boldsymbol{\phi}''' + \boldsymbol{G}'\boldsymbol{\phi}'' + \boldsymbol{G}''\boldsymbol{\phi}' + \boldsymbol{G}'''\boldsymbol{\phi_0} = \boldsymbol{0}. \quad \text{[S5.29d]}$$

The dominant right eigenvector, $\boldsymbol{\phi} = \boldsymbol{\phi_0} + \varepsilon\boldsymbol{\phi}' + \ldots$ of the matrix $\boldsymbol{G}$ corresponding to the eigenvalue 0 is determined sequentially from Eq. (S5.29) and normalization relationships: $\boldsymbol{1}^\top\boldsymbol{\phi} = \boldsymbol{1}^\top(\boldsymbol{\phi_0} + \varepsilon\boldsymbol{\phi}' + \ldots) = 1$, or

$$\begin{aligned}\boldsymbol{1}^\top\boldsymbol{\phi_0} &= 1,\\ \boldsymbol{1}^\top\boldsymbol{\phi}' = \boldsymbol{1}^\top\boldsymbol{\phi}'' &= \boldsymbol{1}^\top\boldsymbol{\phi}''' = \ldots = 0.\end{aligned} \quad \text{[S5.30]}$$

From the zeroth-order eigenequation (S5.29a), we have

$$\begin{aligned}\boldsymbol{G_0}\boldsymbol{\phi_0} &= \left(\frac{1}{n}\begin{pmatrix}1 & \cdots & 1\\ \vdots & \ddots & \vdots\\ 1 & \cdots & 1\end{pmatrix} - \boldsymbol{I}\right)\boldsymbol{\phi_0} = \left(\frac{1}{n}\boldsymbol{1}\boldsymbol{1}^\top - \boldsymbol{I}\right)\boldsymbol{\phi_0} = \frac{1}{n}\boldsymbol{1}(\boldsymbol{1}^\top\boldsymbol{\phi_0}) - \boldsymbol{\phi_0}\\ &= \frac{1}{n}\boldsymbol{1} - \boldsymbol{\phi_0} = \boldsymbol{0},\end{aligned} \quad \text{[S5.31]}$$

where we used the normalization relation (S5.30a). From this, we then see that the zeroth order term of the right eigenvector is the uniform distribution over populations,

$$\boldsymbol{\phi_0} = \frac{\boldsymbol{1}}{n}, \quad \text{[S5.32a]}$$

as shown in Eq. (S5.28a). ■

To solve $\boldsymbol{\phi}'$, $\boldsymbol{\phi}''$, and $\boldsymbol{\phi}'''$ from Eq. (S5.29) and Eq. (S5.30), we first note that $\boldsymbol{G_0}\boldsymbol{\phi}' = (\boldsymbol{1}\boldsymbol{1}^\top/n - \boldsymbol{I})\boldsymbol{\phi}' = -\boldsymbol{\phi}'$, $\boldsymbol{G_0}\boldsymbol{\phi}'' = -\boldsymbol{\phi}''$, and $\boldsymbol{G_0}\boldsymbol{\phi}''' = -\boldsymbol{\phi}'''$ because of the second equation of normalization relationship: $\boldsymbol{1}^\top\boldsymbol{\phi}' = \boldsymbol{0}$, $\boldsymbol{1}^\top\boldsymbol{\phi}'' = \boldsymbol{0}$, and $\boldsymbol{1}^\top\boldsymbol{\phi}''' = \boldsymbol{0}$ (Eq. (S5.30)). Therefore, $\boldsymbol{\phi}'$, $\boldsymbol{\phi}''$, and $\boldsymbol{\phi}'''$ are solved from (S5.29b)-(S5.29d) as

$$\boldsymbol{\phi}' = \boldsymbol{G}'\boldsymbol{\phi_0}, \quad \text{[S5.32b]}$$

$$\begin{aligned}\boldsymbol{\phi}'' &= \boldsymbol{G}'\boldsymbol{\phi}' + \boldsymbol{G}''\boldsymbol{\phi_0}\\ &= (\boldsymbol{G}'^2 + \boldsymbol{G}'')\boldsymbol{\phi_0},\end{aligned} \quad \text{[S5.32c]}$$

$$\begin{aligned}\boldsymbol{\phi}''' &= \boldsymbol{G}'\boldsymbol{\phi}'' + \boldsymbol{G}''\boldsymbol{\phi}' + \boldsymbol{G}'''\boldsymbol{\phi_0}\\ &= (\boldsymbol{G}'^3 + \boldsymbol{G}'\boldsymbol{G}'' + \boldsymbol{G}''\boldsymbol{G}' + \boldsymbol{G}''')\boldsymbol{\phi_0}.\end{aligned} \quad \text{[S5.32d]}$$

Using the definitions Eq. (S5.25) of these matrices ($\boldsymbol{G} = \boldsymbol{J}/m_0$), and noting that the matrices $\mathcal{K}'$, $\mathcal{Y}'$, $\mathcal{Y}''$, and $\mathcal{Y}'''$ are diagonal, we have

$$\begin{aligned}\boldsymbol{G}' &= \frac{b}{m_0}(\mathcal{K}' - \mathcal{Y}') = \frac{b}{m_0}(1-\chi)\mathcal{K}' \\ &= \vartheta\chi\mathcal{K}', \qquad \text{[S5.33a]}\\ \boldsymbol{G}'^2 + \boldsymbol{G}'' &= \vartheta^2\chi^2\mathcal{K}'^2 - \vartheta^2\chi^3\left(\mathcal{K}'^2 + \frac{1-\chi}{\chi}V_k\boldsymbol{I}\right)\\ &= \vartheta^2\chi^2(1-\chi)\left(\mathcal{K}'^2 - V_k\boldsymbol{I}\right), \qquad \text{[S5.33b]}\\ \boldsymbol{G}'^3 + \boldsymbol{G}'\boldsymbol{G}'' + \boldsymbol{G}''\boldsymbol{G}' + \boldsymbol{G}''' &= \vartheta^3\chi^3\mathcal{K}'^3 - 2\vartheta\chi\mathcal{K}'\vartheta^2\chi^3\left(\mathcal{K}'^2 + \frac{1-\chi}{\chi}V_k\boldsymbol{I}\right)\\ &\quad - \vartheta^3\chi^4(1-2\chi)\left[\mathcal{K}'^3 + \frac{1-\chi}{\chi}\left(T_k\boldsymbol{I} + V_k\mathcal{K}'\right)\right]\\ &= \vartheta^3\chi^3(1-\chi)\left[(1-2\chi)\left(\mathcal{K}'^3 - T_k\boldsymbol{I}\right) - (3-2\chi)V_k\mathcal{K}'\right] \qquad \text{[S5.33c]}\end{aligned}$$

Substituting these and Eq. (S5.30) into Eq. (S5.32b)-Eq. (S5.32d), we have

$$\begin{aligned}\boldsymbol{\phi}' &= \frac{1}{n}\vartheta\chi\boldsymbol{k}',\\ \boldsymbol{\phi}'' &= \frac{1}{n}\vartheta^2\chi^2(1-\chi)\left(\boldsymbol{k}'\circ\boldsymbol{k}' - V_k\boldsymbol{1}\right),\\ \boldsymbol{\phi}''' &= \frac{1}{n}\vartheta^3\chi^3(1-\chi)\left[(1-2\chi)\left(\boldsymbol{k}'\circ\boldsymbol{k}'\circ\boldsymbol{k}' - T_k\boldsymbol{1}\right) - (3-2\chi)V_k\boldsymbol{k}'\right],\end{aligned}$$

which completes the derivation of Eq. (S5.28a)–Eq. (S5.28d). ■

S5.5. Expansion of dominant left eigenvector. Here, we show that each term of ε-expansion of the left eigenvector $\boldsymbol{v}^\top = \boldsymbol{v_0}^\top + \varepsilon\boldsymbol{v}'^\top + \varepsilon^2\boldsymbol{v}''^\top + \varepsilon^3\boldsymbol{v}'''^\top + O(\varepsilon^4)$ up to the third-order is

$$\begin{aligned}\boldsymbol{v_0}^\top &= \boldsymbol{1}^\top, \qquad \text{[S5.34a]}\\ \boldsymbol{v}'^\top &= \vartheta\chi\boldsymbol{k}'^\top, \qquad \text{[S5.34b]}\\ \boldsymbol{v}''^\top &= \vartheta^2\chi^2(1-\chi)(\boldsymbol{k}'\circ\boldsymbol{k}')^\top - \vartheta^2\chi^2(2-\chi)V_k\boldsymbol{1}^\top, \qquad \text{[S5.34c]}\\ \boldsymbol{v}'''^\top &= \vartheta^3\chi^3(1-\chi)(1-2\chi)(\boldsymbol{k}'\circ\boldsymbol{k}'\circ\boldsymbol{k}')^\top - \vartheta^3\chi^3(1-\chi)(3-2\chi)T_k\boldsymbol{1}^\top\\ &\quad - \vartheta^3\chi^3(4-5\chi+2\chi^2)V_k\boldsymbol{k}'^\top. \qquad \text{[S5.34d]}\end{aligned}$$

In the rest of this subsection, we derive Eq. (S5.34a)–Eq. (S5.34d) sequentially.

Substituting Eq. (S5.27) into Eq. (S5.26), the left eigenequation corresponding the dominant eigenvalue 0 is

$$(\boldsymbol{v_0} + \varepsilon\boldsymbol{v}' + \ldots)^\top(\boldsymbol{G_0} + \varepsilon\boldsymbol{G}' + \ldots) = \boldsymbol{0}^\top.$$

Equating the terms in the same order of ε powers, we have

$$\begin{aligned}\boldsymbol{v_0}^\top\boldsymbol{G_0} &= \boldsymbol{0}^\top, \qquad \text{[S5.35a]}\\ \boldsymbol{v}'^\top\boldsymbol{G_0} + \boldsymbol{v_0}^\top\boldsymbol{G}' &= \boldsymbol{0}^\top, \qquad \text{[S5.35b]}\\ \boldsymbol{v}''^\top\boldsymbol{G_0} + \boldsymbol{v}'^\top\boldsymbol{G}' + \boldsymbol{v_0}^\top\boldsymbol{G}'' &= \boldsymbol{0}^\top, \qquad \text{[S5.35c]}\\ \boldsymbol{v}'''^\top\boldsymbol{G_0} + \boldsymbol{v}''^\top\boldsymbol{G}' + \boldsymbol{v}'^\top\boldsymbol{G}'' + \boldsymbol{v_0}^\top\boldsymbol{G}''' &= \boldsymbol{0}^\top. \qquad \text{[S5.35d]}\end{aligned}$$

The dominant left eigenvector, $\boldsymbol{v}^\top = \boldsymbol{v_0}^\top + \varepsilon \boldsymbol{v'}^\top + \ldots$ of the matrix $\boldsymbol{G}$ corresponding to the eigenvalue 0 is determined sequentially from Eq. (S5.35) and normalization relationships: $\boldsymbol{v}^\top \boldsymbol{\phi} = (\boldsymbol{v_0} + \varepsilon \boldsymbol{v'} + \ldots)^\top (\boldsymbol{\phi_0} + \varepsilon \boldsymbol{\phi'} + \ldots) = 1$, or

$$\boldsymbol{v_0}^\top \boldsymbol{\phi_0} = 1, \tag{S5.36a}$$

$$\boldsymbol{v'}^\top \boldsymbol{\phi_0} + \boldsymbol{v_0}^\top \boldsymbol{\phi'} = 0, \tag{S5.36b}$$

$$\boldsymbol{v''}^\top \boldsymbol{\phi_0} + \boldsymbol{v'}^\top \boldsymbol{\phi'} + \boldsymbol{v_0}^\top \boldsymbol{\phi''} = 0, \tag{S5.36c}$$

$$\boldsymbol{v'''}^\top \boldsymbol{\phi_0} + \boldsymbol{v''}^\top \boldsymbol{\phi'} + \boldsymbol{v'}^\top \boldsymbol{\phi''} + \boldsymbol{v_0}^\top \boldsymbol{\phi'''} = 0. \tag{S5.36d}$$

Zeroth-order term of left eigenvector From the zeroth order eigenequation (S5.35a), we have

$$\begin{aligned} \boldsymbol{v_0}^\top \boldsymbol{G_0} &= \boldsymbol{v_0}^\top \left(\frac{1}{n} \mathbf{1}\mathbf{1}^\top - \boldsymbol{I} \right) = (\boldsymbol{v_0}^\top \boldsymbol{\phi_0}) \mathbf{1}^\top - \boldsymbol{v_0}^\top \\ &= \mathbf{1}^\top - \boldsymbol{v_0}^\top = \mathbf{0}, \end{aligned} \tag{S5.37}$$

where we used the normalization relation (S5.36a). From this, we then see that the zeroth order term of the right eigenvector is uniform over populations:

$$\boldsymbol{v_0}^\top = \mathbf{1}^\top. \tag{S5.38}$$

This is Eq. (S5.34a) shown in the beginning of this subsection.

First-order term of left eigenvector We obtain the first-order term of the left eigenvector, $\boldsymbol{v'}$, from the left eigenequation of the first order, $\boldsymbol{v'}^\top \boldsymbol{G_0} + \boldsymbol{v_0}^\top \boldsymbol{G'} = \mathbf{0}^\top$, and the normalization relation for left eigenvector of the first order, $\boldsymbol{v'}^\top \boldsymbol{\phi_0} + \boldsymbol{v_0}^\top \boldsymbol{\phi'} = 0$, as follows. We first note that

$$\boldsymbol{v'}^\top \boldsymbol{G_0} = \boldsymbol{v'}^\top \left(\frac{1}{n} \mathbf{1}\mathbf{1}^\top - \boldsymbol{I} \right) = (\boldsymbol{v'}^\top \boldsymbol{\phi_0}) \mathbf{1}^\top - \boldsymbol{v'}^\top$$

because $\boldsymbol{\phi_0} = \mathbf{1}/n$. Substituting $\boldsymbol{v'}^\top \boldsymbol{\phi_0} = -\boldsymbol{v_0}^\top \boldsymbol{\phi'} = 0$ that follows from the above normalization relation for left eigenvector and another normalization relation $\mathbf{1}^\top \boldsymbol{\phi'} = 0$ for right eigenvector, we have

$$\boldsymbol{v'}^\top \boldsymbol{G_0} = -\boldsymbol{v'}.$$

Using this, the left eigenequation of the first order becomes

$$\begin{aligned} \boldsymbol{v'}^\top &= \boldsymbol{v_0}^\top \boldsymbol{G'} = \frac{b}{m_0} (1 - \chi) \boldsymbol{k'}^\top \\ &= \vartheta \chi \boldsymbol{k'}^\top. \end{aligned} \tag{S5.39}$$

This is Eq. (S5.34b) shown in the beginning of this subsection.

Second-order term of left eigenvector Next, we obtain the second-order term of the left eigenvector, $\boldsymbol{v''}$, from the left eigenequation of the second order, $\boldsymbol{v''}^\top \boldsymbol{G_0} + \boldsymbol{v'}^\top \boldsymbol{G'} + \boldsymbol{v_0}^\top \boldsymbol{G''} = \mathbf{0}^\top$, and the normalization relation for left eigenvector of the second order, $\boldsymbol{v''}^\top \boldsymbol{\phi_0} + \boldsymbol{v'}^\top \boldsymbol{\phi'} + \boldsymbol{v_0}^\top \boldsymbol{\phi''} = 0$, as follows. We first note that

$$\boldsymbol{v''}^\top \boldsymbol{G_0} = \boldsymbol{v''}^\top \left(\frac{1}{n} \mathbf{1}\mathbf{1}^\top - \boldsymbol{I} \right) = (\boldsymbol{v''}^\top \boldsymbol{\phi_0}) \mathbf{1}^\top - \boldsymbol{v''}^\top$$

because $\boldsymbol{\phi_0} = \mathbf{1}/n$. Substituting $\boldsymbol{v''}^\top \boldsymbol{\phi_0} = -(\boldsymbol{v'}^\top \boldsymbol{\phi'} + \boldsymbol{v_0}^\top \boldsymbol{\phi''}) = -\boldsymbol{v'}^\top \boldsymbol{\phi'}$ that follows from the above normalization relation for left eigenvector and another normalization relation $\mathbf{1}^\top \boldsymbol{\phi''} = 0$ for right eigenvector, we have

$$\boldsymbol{v''}^\top \boldsymbol{G_0} = -(\boldsymbol{v'}^\top \boldsymbol{\phi'}) \mathbf{1}^\top - \boldsymbol{v''}.$$

Using this, the left eigenequation of the second order becomes

$$
\begin{aligned}
\boldsymbol{v''}^\top &= \boldsymbol{v'}^\top\boldsymbol{G'} + \boldsymbol{v_0}^\top\boldsymbol{G''} - (\boldsymbol{v'}^\top\boldsymbol{\phi'})\mathbf{1}^\top \\
&= \mathbf{1}^\top(\boldsymbol{G'}^2 + \boldsymbol{G''}) - (\boldsymbol{v'}^\top\boldsymbol{\phi'})\mathbf{1}^\top \\
&= \left(\frac{b}{m_0}\right)^2(1-\chi)^2(\boldsymbol{k'}\circ\boldsymbol{k'})^\top - \frac{b}{m_0}\boldsymbol{y''}^\top - \vartheta^2\chi^2\frac{1}{n}|\boldsymbol{k'}|^2\mathbf{1}^\top \\
&= \vartheta^2\chi^2(\boldsymbol{k'}\circ\boldsymbol{k'})^\top - \frac{\vartheta\chi}{1-\chi}\vartheta\chi^2(1-\chi)\left((\boldsymbol{k'}\circ\boldsymbol{k'})^\top + \frac{1-\chi}{\chi}V_k\mathbf{1}^\top\right) - \vartheta^2\chi^2V_k\mathbf{1}^\top \\
&= \vartheta^2\chi^2(1-\chi)(\boldsymbol{k'}\circ\boldsymbol{k'})^\top - \vartheta^2\chi^2(2-\chi)V_k\mathbf{1}^\top. \qquad \text{[S5.40]}
\end{aligned}
$$

The last equation is Eq. (S5.34c) shown in the beginning of this subsection.

Third-order term of left eigenvector Finally, we obtain the third-order term of the left eigenvector, $\boldsymbol{v'''}$, from the left eigenequation of the third order, $\boldsymbol{v'''}^\top\boldsymbol{G_0} + \boldsymbol{v''}^\top\boldsymbol{G'} + \boldsymbol{v'}^\top\boldsymbol{G''} + \boldsymbol{v_0}^\top\boldsymbol{G'''} = \mathbf{0}^\top$, and the normalization relation for left eigenvector of the third order, $\boldsymbol{v'''}^\top\boldsymbol{\phi_0} + \boldsymbol{v''}^\top\boldsymbol{\phi'} + \boldsymbol{v'}^\top\boldsymbol{\phi''} + \boldsymbol{v_0}^\top\boldsymbol{\phi'''} = 0$, as follows. We first note that

$$
\boldsymbol{v'''}^\top\boldsymbol{G_0} = \boldsymbol{v'''}^\top\left(\frac{1}{n}\mathbf{1}\mathbf{1}^\top - \boldsymbol{I}\right) = (\boldsymbol{v'''}^\top\boldsymbol{\phi_0})\mathbf{1}^\top - \boldsymbol{v'''}^\top
$$

because $\boldsymbol{\phi_0} = \mathbf{1}/n$. Substituting $\boldsymbol{v'''}^\top\boldsymbol{\phi_0} = -(\boldsymbol{v''}^\top\boldsymbol{\phi'} + \boldsymbol{v'}^\top\boldsymbol{\phi''} + \boldsymbol{v_0}\boldsymbol{\phi'''}) = -(\boldsymbol{v''}^\top\boldsymbol{\phi'} + \boldsymbol{v'}^\top\boldsymbol{\phi''})$ that follows from the above normalization relation for left eigenvector and another normalization relation $\mathbf{1}^\top\boldsymbol{\phi'''} = 0$ for right eigenvector. Therefore

$$
\boldsymbol{v'''}^\top\boldsymbol{G_0} = -(\boldsymbol{v''}^\top\boldsymbol{\phi'} + \boldsymbol{v'}^\top\boldsymbol{\phi''})\mathbf{1}^\top - \boldsymbol{v'''}.
$$

Using this, the left eigenequation of the third order becomes

$$
\begin{aligned}
\mathbf{0}^\top &= \boldsymbol{v'''}^\top\boldsymbol{G_0} + \boldsymbol{v''}^\top\boldsymbol{G'} + \boldsymbol{v'}^\top\boldsymbol{G''} + \boldsymbol{v_0}^\top\boldsymbol{G'''} \\
&= -(\boldsymbol{v''}^\top\boldsymbol{\phi'} + \boldsymbol{v'}^\top\boldsymbol{\phi''})\mathbf{1}^\top - \boldsymbol{v'''} + \boldsymbol{v''}^\top\boldsymbol{G'} + \boldsymbol{v'}^\top\boldsymbol{G''} + \boldsymbol{v_0}^\top\boldsymbol{G'''}
\end{aligned}
$$

and hence, by substituting $\boldsymbol{\phi'} = \boldsymbol{G'}\boldsymbol{\phi_0}$, $\boldsymbol{\phi''} = (\boldsymbol{G''} + \boldsymbol{G'}^2)\boldsymbol{\phi_0}$, and $\boldsymbol{\phi_0} = \mathbf{1}/n$,

$$
\begin{aligned}
\boldsymbol{v'''}^\top &= \boldsymbol{v''}^\top\boldsymbol{G'} + \boldsymbol{v'}^\top\boldsymbol{G''} + \boldsymbol{v_0}^\top\boldsymbol{G'''} - (\boldsymbol{v''}^\top\boldsymbol{\phi'} + \boldsymbol{v'}^\top\boldsymbol{\phi''})\mathbf{1}^\top \\
&= \boldsymbol{v''}^\top\boldsymbol{G'}\left(\boldsymbol{I} - \frac{1}{n}\mathbf{1}\mathbf{1}^\top\right) + \boldsymbol{v'}^\top\left(\boldsymbol{G''} - (\boldsymbol{G''} + \boldsymbol{G'}^2)\frac{1}{n}\mathbf{1}\mathbf{1}^\top\right) + \mathbf{1}^\top\boldsymbol{G'''} \\
&= -\boldsymbol{v''}^\top\boldsymbol{G'}\boldsymbol{G_0} - \boldsymbol{v'}^\top\boldsymbol{G''}\boldsymbol{G_0} - \boldsymbol{v'}^\top\boldsymbol{G'}^2\frac{1}{n}\mathbf{1}\mathbf{1}^\top + \mathbf{1}^\top\boldsymbol{G'''}. \qquad \text{[S5.41]}
\end{aligned}
$$

The remaining task to obtain $\boldsymbol{v'''}$ is to evaluate the right-hand side of Eq. (S5.41). To simplify the notation, we introduce the following abbreviations:

$$
\boldsymbol{\kappa_1}^\top = \boldsymbol{k'}, \qquad \boldsymbol{\kappa_2}^\top = (\boldsymbol{k'}\circ\boldsymbol{k'})^\top, \qquad \boldsymbol{\kappa_3}^\top = (\boldsymbol{k'}\circ\boldsymbol{k'}\circ\boldsymbol{k'})^\top,
$$

Using these abbreviations, each term of the right-hand side of Eq. (S5.41) is evaluated as follows. By substituting the expressions for $\boldsymbol{v''}$, $\boldsymbol{G'}$, and $\boldsymbol{G_0}$ into the first term of Eq. (S5.41), and noting $\boldsymbol{k'}^\top\mathbf{1} = 0$,

$$
\begin{aligned}
-\boldsymbol{v''}^\top\boldsymbol{G'}\boldsymbol{G_0} &= -\left(\vartheta^2\chi^2(1-\chi)\boldsymbol{\kappa_2}^\top - \vartheta^2\chi^2(2-\chi)V_k\mathbf{1}^\top\right)\vartheta\chi\mathcal{K}'\left(\frac{1}{n}\mathbf{1}\mathbf{1}^\top - \boldsymbol{I}\right) \\
&= -\vartheta^3\chi^3(1-\chi)\frac{1}{n}\boldsymbol{\kappa_2}^\top\boldsymbol{\kappa_1}\mathbf{1}^\top + \vartheta^3\chi^3(1-\chi)\boldsymbol{\kappa_3}^\top - \vartheta^3\chi^3(2-\chi)V_k\boldsymbol{\kappa_1}^\top \\
&= \vartheta^3\chi^3(1-\chi)\left(\boldsymbol{\kappa_3} - T_k\mathbf{1}^\top\right) - \vartheta^3\chi^3(2-\chi)V_k\boldsymbol{\kappa_1}^\top. \qquad \text{[S5.42]}
\end{aligned}
$$

By substituting the expressions for $\boldsymbol{v'}$, $\boldsymbol{G''}$, $\mathcal{Y}''$, and $\boldsymbol{G_0}$ into the second term of Eq. (S5.41),

$$\begin{aligned}
-\boldsymbol{v'}^{\top}\boldsymbol{G''}\boldsymbol{G_0} &= \vartheta\chi\boldsymbol{\kappa_1}^{\top}\frac{\vartheta\chi}{1-\chi}\mathcal{Y}''\left(\frac{1}{n}\mathbf{1}\mathbf{1}^{\top}-\boldsymbol{I}\right)\\
&= \vartheta\chi\boldsymbol{\kappa_1}^{\top}\frac{\vartheta\chi}{1-\chi}\vartheta\chi^2(1-\chi)\left(\mathcal{K}'^2+\frac{1-\chi}{\chi}V_k\boldsymbol{I}\right)\left(\frac{1}{n}\mathbf{1}\mathbf{1}^{\top}-\boldsymbol{I}\right)\\
&= \left(\vartheta^3\chi^4\boldsymbol{\kappa_3}^{\top}+\vartheta^3\chi^3(1-\chi)V_k\boldsymbol{\kappa_1}^{\top}\right)\left(\frac{1}{n}\mathbf{1}\mathbf{1}^{\top}-\boldsymbol{I}\right)\\
&= \vartheta^3\chi^4T_k\mathbf{1}^{\top}-\vartheta^3\chi^4\boldsymbol{\kappa_3}^{\top}-\vartheta^3\chi^3(1-\chi)V_k\boldsymbol{\kappa_1}^{\top}\\
&= -\vartheta^3\chi^4\left(\boldsymbol{\kappa_3}^{\top}-T_k\mathbf{1}^{\top}\right)-\vartheta^3\chi^3(1-\chi)V_k\boldsymbol{\kappa_1}^{\top}.
\end{aligned} \qquad \text{[S5.43]}$$

By substituting the expressions for $\boldsymbol{v'}$, $\boldsymbol{G'}$ into the third term of Eq. (S5.41),

$$\begin{aligned}
-\boldsymbol{v'}^{\top}\boldsymbol{G'}^2\frac{1}{n}\mathbf{1}\mathbf{1}^{\top} &= -\vartheta\chi\boldsymbol{\kappa_1}^{\top}\vartheta^2\chi^2\mathcal{K}'^2\frac{1}{n}\mathbf{1}\mathbf{1}^{\top}\\
&= -\vartheta^3\chi^3T_k\mathbf{1}^{\top}.
\end{aligned} \qquad \text{[S5.44]}$$

Finally, by substituting the expressions for $\boldsymbol{G'''}$ and $\mathcal{Y}'''$ into the fourth term of Eq. (S5.41),

$$\begin{aligned}
\mathbf{1}^{\top}\boldsymbol{G'''} &= -\mathbf{1}^{\top}\frac{\vartheta\chi}{1-\chi}\mathcal{Y}'''\\
&= -\mathbf{1}^{\top}\frac{\vartheta\chi}{1-\chi}\vartheta^2\chi^3(1-\chi)(1-2\chi)\left(\mathcal{K}'^3+\frac{1-\chi}{\chi}\left(T_k\boldsymbol{I}+V_k\mathcal{K}'\right)\right)\\
&= -\vartheta^3\chi^4(1-2\chi)\left(\boldsymbol{\kappa_3}^{\top}+\frac{1-\chi}{\chi}\left(T_k\mathbf{1}^{\top}+V_k\boldsymbol{k_1}^{\top}\right)\right).
\end{aligned} \qquad \text{[S5.45]}$$

Combining these, and using the original abbreviations rather than $\boldsymbol{k_1}$, $\boldsymbol{k_3}$, $\mathcal{V}_k$ and $\mathcal{T}_k$, the third-order term of the left eigenvector $\boldsymbol{v'''}^{\top}$ is obtained as

$$\begin{aligned}
\boldsymbol{v'''}^{\top} = {}& \vartheta^3\chi^3(1-\chi)(1-2\chi)(\boldsymbol{k'}\circ\boldsymbol{k'}\circ\boldsymbol{k'})^{\top}-\vartheta^3\chi^3(1-\chi)(3-2\chi)T_k\mathbf{1}^{\top}\\
&-\vartheta^3\chi^3(4-5\chi+2\chi^2)V_k\boldsymbol{k'}^{\top}.
\end{aligned}$$

This completes the derivation of Eq. (S5.34a)–Eq. (S5.34d). ■

S5.6. Expansion of selection gradient . In this subsection, using the results obtained in S5.1-S5.5, we derive the selection gradient for pathogen virulence and infectivity in heterogeneous metapopulation up to the fourth order of the degree of heterogeneity:

$$\mathcal{S}(\alpha)=\mathcal{S}_0(\alpha)+\varepsilon\mathcal{S}'(\alpha)+\varepsilon^2\mathcal{S}''(\alpha)+\varepsilon^3\mathcal{S}'''(\alpha)+\varepsilon^4\mathcal{S}''''(\alpha)+O(\varepsilon^5) \qquad \text{[S5.46]}$$

where

$$\mathcal{S}_0(\alpha)=\frac{\mathrm{d}b}{\mathrm{d}\alpha}(1-y_0)-1=\frac{\mathrm{d}b}{\mathrm{d}\alpha}x_0-1, \qquad \text{[S5.47a]}$$

$$\mathcal{S}'(\alpha)=0, \qquad \text{[S5.47b]}$$

$$\mathcal{S}''(\alpha)=\frac{\mathrm{d}b}{\mathrm{d}\alpha}\vartheta\chi(1-\chi)V_k, \qquad \text{[S5.47c]}$$

$$\mathcal{S}'''(\alpha)=\frac{\mathrm{d}b}{\mathrm{d}\alpha}2\vartheta^2\chi^2(1-\chi)^2T_k, \qquad \text{[S5.47d]}$$

$$\mathcal{S}''''(\alpha)=\frac{\mathrm{d}b}{\mathrm{d}\alpha}\vartheta^3\chi^3(1-\chi)\left[\left(3-8\chi+5\chi^2\right)Q_k-\left(8-12\chi+5\chi^2\right){V_k}^2\right] \qquad \text{[S5.47e]}$$

where $b = \beta K_0$ is the scaled transmission rate and $Q_k = \frac{1}{n}\sum_{i=1}^{n} k_i'^4$ is the fourth central moment for the variation of the scaled carrying capacities around the metapopulation average.

In the rest of this subsection, we will show Eq. (S5.47a)–Eq. (S5.47e) step by step. We start with the definition of the selection gradient

$$\mathcal{S}(\alpha) = \frac{\mathrm{d}b}{\mathrm{d}\alpha}\boldsymbol{v}^\top \boldsymbol{\Sigma}\boldsymbol{\phi} - 1 \quad \text{[S5.48]}$$

with

$$\boldsymbol{\Sigma} = \boldsymbol{\Sigma_0} + \varepsilon\boldsymbol{\Sigma'} + \varepsilon^2\boldsymbol{\Sigma''} + \varepsilon^3\boldsymbol{\Sigma'''} + O(\varepsilon^4), \quad \text{[S5.49]}$$

$$\boldsymbol{v}^\top = \boldsymbol{v_0} + \varepsilon\boldsymbol{v'} + \varepsilon^2\boldsymbol{v''} + \varepsilon^3\boldsymbol{v''''} + O(\varepsilon^4), \quad \text{[S5.50]}$$

$$\boldsymbol{\phi}^\top = \boldsymbol{\phi_0} + \varepsilon\boldsymbol{\phi'} + \varepsilon^2\boldsymbol{\phi''} + \varepsilon^3\boldsymbol{\phi'''} + O(\varepsilon^4), \quad \text{[S5.51]}$$

where $\boldsymbol{\phi_0}$, $\boldsymbol{\phi'}$, $\boldsymbol{\phi''}$, $\boldsymbol{\phi'''}$, $\boldsymbol{v_0}^\top$, $\boldsymbol{v'}^\top$, $\boldsymbol{v''}^\top$, $\boldsymbol{v'''}^\top$ are obtained in the last two subsections, $\boldsymbol{\Sigma_0}$, $\boldsymbol{\Sigma'}$, ... are n dimensional diagonal matrices defined as

$$\boldsymbol{\Sigma_0} = x_0\boldsymbol{I} = (1 - y_0)\boldsymbol{I}, \quad \text{[S5.52a]}$$

$$\boldsymbol{\Sigma'} = \mathcal{K}' - \mathcal{Y}' = (1-\chi)\mathcal{K}', \quad \text{[S5.52b]}$$

$$\boldsymbol{\Sigma''} = -\mathcal{Y}'', \quad \text{[S5.52c]}$$

$$\boldsymbol{\Sigma'''} = -\mathcal{Y}''', \quad \text{[S5.52d]}$$

where $\mathcal{K}'$, $\mathcal{Y}'$, $\mathcal{Y}''$ and $\mathcal{Y}'''$ are diagonal matrices whose diagonal components are given by k_i', y_i', y_i'', and y_i''', respectively ($i = 1, \ldots, n$) obtained in S5.2. With these quantities already defined, the selection gradient is expanded in a Taylor series with respect to the degree ε of metapopulation heterogeneity as Eq. (S5.46).

Zeroth-order selection gradient The zeroth term of the selection gradient for pathogen virulence α Eq. (S5.48) is given by

$$\begin{aligned}\mathcal{S}_0(\alpha) &= \frac{\mathrm{d}b}{\mathrm{d}\alpha}\boldsymbol{v_0}^\top\boldsymbol{\Sigma_0}\boldsymbol{\phi_0} - 1 = \frac{\mathrm{d}b}{\mathrm{d}\alpha}\boldsymbol{1}^\top(1-y_0)\boldsymbol{I}\boldsymbol{1}/n - 1\\ &= \frac{\mathrm{d}b}{\mathrm{d}\alpha}(1-y_0) - 1 = \frac{\mathrm{d}b}{\mathrm{d}\alpha}x_0 - 1. \end{aligned} \quad \text{[S5.53]}$$

This is the same as the selection gradient for virulence α in a single population, and is Eq. (S5.47a) to be shown. ■

First-order term of selection gradient The first-order term of the selection gradient Eq. (S5.48) for pathogen virulence α is given by

$$\begin{aligned}\mathcal{S}'(\alpha) &= \frac{\mathrm{d}b}{\mathrm{d}\alpha}\left(\boldsymbol{v'}^\top\boldsymbol{\Sigma_0}\boldsymbol{\phi_0} + \boldsymbol{v_0}^\top\boldsymbol{\Sigma_0}\boldsymbol{\phi'} + \boldsymbol{v_0}^\top\boldsymbol{\Sigma'}\boldsymbol{\phi_0}\right)\\ &= \frac{\mathrm{d}b}{\mathrm{d}\alpha}\left(x_0(\boldsymbol{v'}^\top\boldsymbol{\phi_0} + \boldsymbol{v_0}^\top\boldsymbol{\phi'}) + \boldsymbol{1}^\top\boldsymbol{\Sigma'}\frac{\boldsymbol{1}}{n}\right)\\ &= \frac{\mathrm{d}b}{\mathrm{d}\alpha}\boldsymbol{1}^\top(1-\chi)\mathcal{K}'\frac{\boldsymbol{1}}{n}\\ &= \frac{\mathrm{d}b}{\mathrm{d}\alpha}\frac{\boldsymbol{1}^\top\boldsymbol{k'}}{n} = 0, \end{aligned} \quad \text{[S5.54]}$$

where we used $\boldsymbol{v'}^\top\boldsymbol{\phi_0} + \boldsymbol{v_0}^\top\boldsymbol{\phi'} = 0$ that follows from the normalization condition for left eigenvector, $\boldsymbol{\Sigma'} = (1-\chi)\mathcal{K}$, and $\boldsymbol{1}^\top\boldsymbol{k'} = 0$. This completes the derivation of Eq. (S5.47b). ■

Second-order term of selection gradient Collecting the terms of the second-power of ε in the selection gradient Eq. (S5.48) for pathogen virulence α, we have

$$\begin{aligned}\mathcal{S}''(\alpha) &= \frac{\mathrm{d}b}{\mathrm{d}\alpha}\left(\boldsymbol{v}''^{\top}\boldsymbol{\Sigma_0}\boldsymbol{\phi_0} + \boldsymbol{v}'^{\top}\boldsymbol{\Sigma_0}\boldsymbol{\phi}' + \boldsymbol{v_0}^{\top}\boldsymbol{\Sigma_0}\boldsymbol{\phi}'' + \boldsymbol{v}'^{\top}\boldsymbol{\Sigma}'\boldsymbol{\phi_0} + \boldsymbol{v_0}^{\top}\boldsymbol{\Sigma}'\boldsymbol{\phi}' + \boldsymbol{v_0}^{\top}\boldsymbol{\Sigma}''\boldsymbol{\phi_0}\right)\\ &= \frac{\mathrm{d}b}{\mathrm{d}\alpha}\left(\boldsymbol{v_0}^{\top}\left(\boldsymbol{\Sigma_0}\boldsymbol{\phi}'' + \boldsymbol{\Sigma}'\boldsymbol{\phi}' + \boldsymbol{\Sigma}''\boldsymbol{\phi_0}\right) + \left(\boldsymbol{v}'^{\top}\boldsymbol{\Sigma_0}\boldsymbol{\phi}' + \boldsymbol{v}''^{\top}\boldsymbol{\Sigma_0}\boldsymbol{\phi_0}\right) + \boldsymbol{v}'^{\top}\boldsymbol{\Sigma}'\boldsymbol{\phi_0}\right)\\ &= \frac{\mathrm{d}b}{\mathrm{d}\alpha}\boldsymbol{v}'^{\top}\boldsymbol{\Sigma}'\boldsymbol{\phi_0}, \end{aligned} \qquad \text{[S5.55]}$$

where we used $\boldsymbol{v_0}^{\top}(\boldsymbol{\Sigma_0}\boldsymbol{\phi}''+\boldsymbol{\Sigma}'\boldsymbol{\phi}''+\boldsymbol{\Sigma}'\boldsymbol{\phi}'+\boldsymbol{\Sigma}''\boldsymbol{\phi_0}) = 0$ that follows by multiplying $\boldsymbol{v_0}^{\top}$ from left to the second-order right eigenequation, and $\boldsymbol{v}'^{\top}\boldsymbol{\Sigma_0}\boldsymbol{\phi}' + \boldsymbol{v}''^{\top}\boldsymbol{\Sigma_0}\boldsymbol{\phi_0} = \boldsymbol{v}'^{\top}x_0\boldsymbol{I}\boldsymbol{\phi}' + \boldsymbol{v}''^{\top}x_0\boldsymbol{I}\boldsymbol{\phi_0} = x_0\left(\boldsymbol{v}'^{\top}\boldsymbol{\phi}' + \boldsymbol{v}''^{\top}\boldsymbol{\phi_0}\right) = 0$ that follows by combining the second order normalization condition for $\boldsymbol{v}^{\top}$, $\boldsymbol{v}''^{\top}\boldsymbol{\phi_0} + \boldsymbol{v}'^{\top}\boldsymbol{\phi}' + \boldsymbol{v_0}^{\top}\boldsymbol{\phi}'' = 0$ and the second order normalization condition for $\boldsymbol{\phi}$, $\boldsymbol{1}^{\top}\boldsymbol{\phi}'' = \boldsymbol{v_0}^{\top}\boldsymbol{\phi}'' = 0$. Therefore, by substituting $\boldsymbol{v}'$ obtained in S5.5 and $\boldsymbol{\Sigma}' = (1-\chi)\mathcal{K}$ into Eq. (S5.55), we have

$$\begin{aligned}\mathcal{S}''(\alpha) &= \frac{\mathrm{d}b}{\mathrm{d}\alpha}\vartheta\chi\boldsymbol{k}'^{\top}(1-\chi)\mathcal{K}'\frac{\boldsymbol{1}}{n} = \frac{\mathrm{d}b}{\mathrm{d}\alpha}\vartheta\chi(1-\chi)\frac{\boldsymbol{k}'^{\top}\boldsymbol{k}'}{n}\\ &= \frac{\mathrm{d}b}{\mathrm{d}\alpha}\vartheta\chi(1-\chi)V_k. \end{aligned} \qquad \text{[S5.56]}$$

This completes the derivation of Eq. (S5.47c). ■

Third-order term of selection gradient The third-order term of the selection gradient Eq. (S5.48) for pathogen virulence α is given by

$$\begin{aligned}\mathcal{S}'''(\alpha) &= \frac{\mathrm{d}b}{\mathrm{d}\alpha}\Big(\boldsymbol{v}'''^{\top}\boldsymbol{\Sigma_0}\boldsymbol{\phi_0} + \boldsymbol{v}''^{\top}\boldsymbol{\Sigma_0}\boldsymbol{\phi}' + \boldsymbol{v}'^{\top}\boldsymbol{\Sigma_0}\boldsymbol{\phi}'' + \boldsymbol{v_0}^{\top}\boldsymbol{\Sigma_0}\boldsymbol{\phi}'''\\ &\qquad + \boldsymbol{v}''^{\top}\boldsymbol{\Sigma}'\boldsymbol{\phi_0} + \boldsymbol{v}'^{\top}\boldsymbol{\Sigma}'\boldsymbol{\phi}' + \boldsymbol{v_0}^{\top}\boldsymbol{\Sigma}'\boldsymbol{\phi}'' + \boldsymbol{v}'^{\top}\boldsymbol{\Sigma}''\boldsymbol{\phi_0} + \boldsymbol{v_0}^{\top}\boldsymbol{\Sigma}''\boldsymbol{\phi}' + \boldsymbol{v_0}^{\top}\boldsymbol{\Sigma}'''\boldsymbol{\phi_0}\Big),\\ &= \frac{\mathrm{d}b}{\mathrm{d}\alpha}\Big(\boldsymbol{v_0}^{\top}\left(\boldsymbol{\Sigma_0}\boldsymbol{\phi}''' + \boldsymbol{\Sigma}'\boldsymbol{\phi}'' + \boldsymbol{\Sigma}''\boldsymbol{\phi}' + \boldsymbol{\Sigma}'''\boldsymbol{\phi_0}\right) + \left(\boldsymbol{v}'''^{\top}\boldsymbol{\Sigma_0}\boldsymbol{\phi_0} + \boldsymbol{v}''^{\top}\boldsymbol{\Sigma_0}\boldsymbol{\phi}' + \boldsymbol{v}'^{\top}\boldsymbol{\Sigma_0}\boldsymbol{\phi}''\right)\\ &\qquad + \boldsymbol{v}''^{\top}\boldsymbol{\Sigma}'\boldsymbol{\phi_0} + \boldsymbol{v}'^{\top}\boldsymbol{\Sigma}'\boldsymbol{\phi}' + \boldsymbol{v}'^{\top}\boldsymbol{\Sigma}''\boldsymbol{\phi_0}\Big)\\ &= \frac{\mathrm{d}b}{\mathrm{d}\alpha}\left(\boldsymbol{v}''^{\top}\boldsymbol{\Sigma}'\boldsymbol{\phi_0} + \boldsymbol{v}'^{\top}\boldsymbol{\Sigma}'\boldsymbol{\phi}' + \boldsymbol{v}'^{\top}\boldsymbol{\Sigma}''\boldsymbol{\phi_0}\right) \end{aligned} \qquad \text{[S5.57]}$$

where we used $\boldsymbol{v_0}^{\top}(\boldsymbol{\Sigma_0}\boldsymbol{\phi}'''+\boldsymbol{\Sigma}'\boldsymbol{\phi}''+\boldsymbol{\Sigma}''\boldsymbol{\phi}'+\boldsymbol{\Sigma}'''\boldsymbol{\phi_0}) = 0$ that follow by multiplying $\boldsymbol{v_0}^{\top}$ from left to the third-order right eigenequation, and $\boldsymbol{v}'''^{\top}\boldsymbol{\Sigma_0}\boldsymbol{\phi_0} + \boldsymbol{v}''^{\top}\boldsymbol{\Sigma_0}\boldsymbol{\phi}' + \boldsymbol{v}'^{\top}\boldsymbol{\Sigma_0}\boldsymbol{\phi}'' = x_0\left(\boldsymbol{v}'''^{\top}\boldsymbol{\phi_0} + \boldsymbol{v}''^{\top}\boldsymbol{\phi}' + \boldsymbol{v}'^{\top}\boldsymbol{\phi}''\right) = 0$ and, using $\boldsymbol{v_0}^{\top}\boldsymbol{\phi}''' = \boldsymbol{1}^{\top}\boldsymbol{\phi}''' = 0$ that follows by combining the third order normalization condition for $\boldsymbol{v}^{\top}$, $\boldsymbol{v}'''^{\top}\boldsymbol{\phi_0} + \boldsymbol{v}''^{\top}\boldsymbol{\phi}' + \boldsymbol{v}'^{\top}\boldsymbol{\phi}'' + \boldsymbol{v_0}^{\top}\boldsymbol{\phi}''' = 0$ and the third order normalization condition for $\boldsymbol{\phi}$, $\boldsymbol{1}^{\top}\boldsymbol{\phi}''' = \boldsymbol{v_0}^{\top}\boldsymbol{\phi}''' = 0$. Therefore, by substituting $\boldsymbol{v}''$, $\boldsymbol{v}'$, and $\boldsymbol{\phi}'$ obtained in S5.4 and S5.5, $\boldsymbol{\Sigma}' = (1-\chi)\mathcal{K}$, and $\boldsymbol{\Sigma}'' = -\mathcal{Y}''$ into Eq. (S5.57), we have

$$\begin{aligned}\mathcal{S}'''(\alpha) &= \frac{\mathrm{d}b}{\mathrm{d}\alpha}\Bigg[\left(\vartheta^2\chi^2(1-\chi)(\boldsymbol{k}'\circ\boldsymbol{k}')^{\top} - \vartheta^2\chi^2(2-\chi)V_k\boldsymbol{1}^{\top}\right)(1-\chi)\mathcal{K}'\frac{\boldsymbol{1}}{n}\\ &\qquad + \vartheta\chi\boldsymbol{k}'^{\top}(1-\chi)\mathcal{K}'\frac{1}{n}\vartheta\chi\boldsymbol{k}' + \vartheta\chi\boldsymbol{k}'^{\top}\left(-\vartheta\chi^2(1-\chi)\left(\mathcal{K}'^2 + \frac{1-\chi}{\chi}V_k\boldsymbol{I}\right)\right)\frac{\boldsymbol{1}}{n}\Bigg]\\ &= \frac{\mathrm{d}b}{\mathrm{d}\alpha}\left[\vartheta^2\chi^2(1-\chi)^2T_k + \vartheta^2\chi^2(1-\chi)T_k - \vartheta^2\chi^3(1-\chi)T_k\right]\\ &= \frac{\mathrm{d}b}{\mathrm{d}\alpha}2\vartheta^2\chi^2(1-\chi)^2T_k. \end{aligned} \qquad \text{[S5.58]}$$

This completes the derivation of Eq. (S5.47d). ■

The derivation of the expression Eq. (S5.47e) for $\mathcal{S}''''(\alpha)$ takes more pages when spelling out but is completely analogous to those of $\mathcal{S}''(\alpha)$ and $\mathcal{S}'''(\alpha)$ (Eq. (S5.47c)-Eq. (S5.47d)) described above, and hence omitted from here. See online Mathematica source code ("file-name.nb") to check Eq. (S5.47e).

S5.7. Increment of ESS virulence. We now evaluate ESS virulence α^* in a Taylor series with respect to ε as

$$\alpha^* = \alpha_0^* + \varepsilon\alpha_1^* + \varepsilon^2\alpha_2^* + \varepsilon^3\alpha_3^* + \varepsilon^4\alpha_4^* + O(\varepsilon^5). \qquad [S5.59]$$

We substitute (S5.59) into the expression for selection gradient, Eq. (S5.46)-Eq. (S5.47e), and equate it to zero to obtain the ESS virulence: α^*. We then have the expression of ESS virulence up to the fourth order.

ESS for general tradeoffs For any monotonically increasing tradeoff $\beta = \beta(\alpha)$ between transmission and virulence, the zeroth order approximation for ESS virulence (which is an exact ESS in a homogeneous metapopulation) is determined from

$$\frac{\mathrm{d}b}{\mathrm{d}\alpha}(\alpha_0^*) = \frac{b(\alpha_0^*)}{v(\alpha_0^*)} = \frac{b(\alpha_0^*)}{\delta + \alpha_0^*} = \frac{\vartheta_0}{\vartheta_0 - 1} \qquad [S5.60]$$

where $\delta = \mu + \eta$ and ϑ_0 denotes $\vartheta = b/(b - v)$ at $\alpha = \alpha_0^*$: $\vartheta_0 = b(\alpha_0^*)/(b(\alpha_0^*) - (\delta + \alpha_0^*))$.

The first-order increment of ESS virulence is found to be

$$\alpha_1^* = 0. \qquad [S5.61]$$

The second-order increment is found to be proportional to the variance of carrying capacities over metapopulation

$$\alpha_2^* \frac{\vartheta_0^3}{(\vartheta_0 - 1)^2} \frac{\chi_0(1 - \chi_0)}{\left(-\frac{\mathrm{d}^2 b}{\mathrm{d}\alpha^2}(\alpha_0^*)\right)} V_k, \qquad [S5.62]$$

where χ_0 is the value of $\chi = (b(\alpha) - (\delta + \alpha))/(b(\alpha) - (\delta + \alpha) + m_0)$ evaluated at $\alpha = \alpha_0^*$ and $V_k = \sum_i {k_i'}^2/n$ is the metapopulation variance of the scaled carrying capacities as defined before.

The third-order increment is found to be proportional to the third central moment $T_k = \sum_i {k_i'}^3/n$ of scaled carrying capacities

$$\alpha_3^* = \frac{\vartheta_0^4}{(\vartheta_0 - 1)^2} \frac{2\chi_0^2(1 - \chi_0)^2}{\left(-\frac{\mathrm{d}^2 b}{\mathrm{d}\alpha^2}(\alpha_0^*)\right)} T_k. \qquad [S5.63]$$

The fourth-order increment α_4^* of ESS virulence is found to be expressed as a function of the fourth-order central moment $Q_k = \sum_i {k_i'}^4/n$ and the variance of scaled carrying capacities as

$$\alpha_4^* = \frac{\vartheta_0^5}{(\vartheta_0 - 1)^2} \frac{\chi_0^2(1 - \chi_0)^2}{\left(-\frac{\mathrm{d}^2 b}{\mathrm{d}\alpha^2}(\alpha_0^*\right)} \Bigg[\chi_0(3 - 5\chi_0)\mathcal{Q}_k$$

$$+ \left\{ \frac{\vartheta_0}{2(\vartheta_0 - 1)^2} \frac{\frac{\mathrm{d}^3 b}{\mathrm{d}\alpha^3}(\alpha_0^*)}{\left(-\frac{\mathrm{d}^2 b}{\mathrm{d}\alpha^2}(\alpha_0^*)\right)^2} + \frac{\vartheta_0^2}{2(\vartheta_0 - 1)^3} \frac{3 - 4\chi_0}{\left(-\frac{\mathrm{d}^2 b}{\mathrm{d}\alpha^2}(\alpha_0^*)\right) b(\alpha_0^*)} \right.$$

$$\left. - \left(\frac{1}{\vartheta_0 - 1} + \frac{\chi_0}{1 - \chi_0}(8 - 12\chi_0 + 5\chi_0^2) \right) \right\} {\mathcal{V}_k}^2 \Bigg]. \qquad [S5.64]$$

ESS for a specific tradeoff Although we have the general expression of ESS virulence with a general tradeoff form of $\beta(\alpha)$ up to fourth-order terms of the degree of metapopulation heterogeneity, δ, we here show the result for a specific form,

$$\beta(\alpha) = a\sqrt{\alpha},$$

used in numerical examples in the main body. Here we assume that $K_0 = 1$ and hence $b = \beta$. The ESS virulence in homogeneous metapopulation maximizes the basic reproduction ratio, $\mathscr{R}_0 = \beta(\alpha)/(\delta+\alpha) = a\sqrt{\alpha}/(\delta+\alpha)$, or

$$\alpha_0^* = \delta. \tag{S5.65}$$

The first-order increment of ESS virulence in heterogeneous metapopulation is zero as in the case of general tradeoff: $\alpha_1^* = 0$. The second, third, and fourth-order increments of ESS virulence, obtained for general tradeoff $\beta = \beta(\alpha)$ as Eq. (S5.62)–Eq. (S5.64) are expressed with this tradeoff, $\beta = a\sqrt{\alpha}$, as

$$\alpha_2^* = \frac{a^2 m_0 \delta}{\left(m_0 + a\sqrt{\delta} - 2\delta\right)^2} V_k, \tag{S5.66}$$

$$\alpha_3^* = 2\frac{a^3 {m_0}^2 \delta^{3/2}}{\left(m_0 + a\sqrt{\delta} - 2\delta\right)^4} T_k, \tag{S5.67}$$

$$\alpha_4^* = \frac{a^4 m_0 \delta}{\left(m_0 + a\sqrt{\delta} - 2\delta\right)^6}\Bigg[m_0\delta\left(3m_0 - 2\left(a - 2\sqrt{\delta}\right)\sqrt{\delta}\right)Q_k + \left\{{m_0}^3 + {m_0}^2\left(a\sqrt{\delta} - 10\delta\right) - 4m_0\left(a - 2\sqrt{\delta}\right)\delta^{3/2} - \left(a - 2\sqrt{\delta}\right)^2\delta^2\right\}V_k^2\Bigg]. \tag{S5.68}$$

S6. Robustness of our results for more general circumstances

We have shown, in our simple SIRS model with density-dependent transmissions (see S1-S5), that metapopulation heterogeneity in birth rates, carrying capacities, immunity loss rates, and movement rates always increases the virulence and infection rate that evolves. Here, we extend our results into other epidemiological scenarios, such as frequency-dependent transmission models that are often assumed in the models of sexually transmitted and vectorborne diseases, superinfection models that are known to increase ESS virulence, and density-dependent host mortality models that allows evolutionary branching to occur.

S6.1. Frequency-dependent transmission. We assume that the transmission rate of the pathogen from infected to susceptible host is frequency dependent as in the vectorborne and the sexually transmitted disease models. We extend a simple SIRS model in Eq. (8) in the main body by changing the transmission term from $\beta S_i I_i$ to $\beta(S_i/N_i)I_i$:

$$\frac{\mathrm{d}S_i}{\mathrm{d}t} = r_i\left(1 - \frac{N_i}{K_i}\right)N_i - \mu S_i + \eta I_i + \nu_i R_i - \frac{\beta S_i I_i}{N_i} + \frac{1}{n}\sum_j \widetilde{m}_{ij} S_j - \frac{1}{n}\sum_j \widetilde{m}_{ji} S_i, \tag{S6.1}$$

$$\frac{\mathrm{d}I_i}{\mathrm{d}t} = \frac{\beta S_i I_i}{N_i} - (\mu + \alpha + \eta + \gamma)I_i + \frac{1}{n}\sum_j \widetilde{m}_{ij} I_j - \frac{1}{n}\sum_j \widetilde{m}_{ji} I_i, \tag{S6.2}$$

$$\frac{\mathrm{d}R_i}{\mathrm{d}t} = \gamma I_i - (\mu + \nu_i)R_i + \frac{1}{n}\sum_j \widetilde{m}_{ij} R_j - \frac{1}{n}\sum_j \widetilde{m}_{ji} R_i. \tag{S6.3}$$

S6.1.1. Analytical results for frequency-dependent transmission model. As the only difference of the frequency-dependent transmission model [S6.1]-[S6.3] from mass-action transmission model (Eq. 8 in the main body) is to replace the local density of susceptible hosts, S_i, by the local fraction of susceptible hosts, $x_i = S_i/N_i$, the same results as shown in S1-S5 hold by replacing ${S_i}^*$ with x_i^*, which will be shown in more detail below.

What is to be changed in the Jacobian of the invasion dynamics of a mutant? The ij elements of the Jacobian matrix $\hat{\boldsymbol{J}}$ of the invasion dynamics of a mutant having a different virulence $\hat{\alpha}$ and transmission rate $\hat{\beta} = \beta(\hat{\alpha})$

 Masato Sato, Ulf Dieckmann and Akira Sasaki

than those (α and $\beta = \beta(\alpha)$) of the resident are defined as

$$\hat{J}_{ii} = \beta(\hat{\alpha})x_i^* - (\mu + \hat{\alpha} + \eta + \gamma) - \sum_j \widetilde{m}_{ji}/n, \tag{S6.4}$$

$$\hat{J}_{ij} = \widetilde{m}_{ij}/n, \qquad (i \neq j), \tag{S6.5}$$

where x_i^* is the equilibrium fraction of susceptible hosts in the ith population of the resident. The only change from our Jacobian in the main body (SI Appendix S2) is to replace the equilibrium density S_i^* by the equilibrium fraction $x_i^* = S_i^*/N_i^*$. We do not need to obtain an explicit form of x_i^* to derive the following results.

Selection gradient for pathogen trait in a metapopulation As in the main body, the selection gradient for a resident virulence α is defined as

$$\mathcal{S}(\alpha) = \boldsymbol{v}^\top \left(\left. \frac{\mathrm{d}\hat{\boldsymbol{J}}}{\mathrm{d}\hat{\alpha}} \right|_{\hat{\alpha}=\alpha} \right) \boldsymbol{\phi}, \tag{S6.6a}$$

where $\boldsymbol{v}$ and $\boldsymbol{\phi}$ are the left and right eigenvectors of the Jacobian evaluated at the resident trait: $\boldsymbol{J} = \hat{\boldsymbol{J}}\big|_{\hat{\alpha}=\alpha}$ (its corresponding eigenvalue is zero), and $\frac{\mathrm{d}\hat{\boldsymbol{J}}}{\mathrm{d}\hat{\alpha}}$ denotes the element-wise differentiation of a matrix $\hat{\boldsymbol{J}}$ by the virulence $\hat{\alpha}$ of a mutant. The matrix $\frac{\mathrm{d}\hat{\boldsymbol{J}}}{\mathrm{d}\hat{\alpha}}\Big|_{\hat{\alpha}=\alpha}$ is a diagonal matrix with diagonal elements

$$\left(\left. \frac{\mathrm{d}\hat{\boldsymbol{J}}}{\mathrm{d}\hat{\alpha}} \right|_{\hat{\alpha}=\alpha} \right)_{ii} = \frac{\mathrm{d}\beta}{\mathrm{d}\alpha}(\alpha)x_i^* - 1. \tag{S6.6b}$$

Expansion of selection gradient with small heterogeneity Now we expand the selection gradient Eq. (S6.6) assuming that the degree ε of metapopulation heterogeneity is small. The equilibrium fraction of susceptible hosts is expanded as

$$x_i^* = x_0^* + \varepsilon\xi_i' + \varepsilon^2\xi_i'' + O(\varepsilon^3) \tag{S6.7}$$

The eigenvectors are also expanded as $\boldsymbol{v} = \boldsymbol{v_0} + \varepsilon\boldsymbol{v'} + \cdots$ and $\boldsymbol{\phi} = \boldsymbol{\phi_0} + \varepsilon\boldsymbol{\phi'} + \cdots$, where $\boldsymbol{v_0} = \boldsymbol{1} = (1, 1, \cdots, 1)^\top$, $\boldsymbol{\phi_0} = \boldsymbol{1}/n$, and

$$\boldsymbol{\phi'} = \frac{\beta\boldsymbol{\xi'} + \boldsymbol{T'}}{nm_0}, \tag{S6.8a}$$

$$\boldsymbol{v'} = \frac{\beta\boldsymbol{\xi'}}{nm_0}, \tag{S6.8b}$$

where $\boldsymbol{\xi'} = (\xi_1', \xi_2', \cdots, \xi_n')^\top$ is the vector of the first order deviation of equilibrium susceptible fractions at local populations and $\boldsymbol{T'} = (T_1', T_2', \cdots, T_n')^\top$ is the vector of net movement inflows to local populations: $T_i' = \sum_j(\widetilde{m}_{ij}' - \widetilde{m}_{ji}')/n$. The only difference here is still the replacement of the vector of the first order deviations of the equilibrium numbers of susceptible hosts $\boldsymbol{S^{*\prime}} = (S_1^{*\prime}, S_2^{*\prime}, \cdots, S_n^{*\prime})^\top$ by the corresponding vector for the susceptible fractions $\boldsymbol{\xi'}$. The selection gradient is then expanded as

$$\mathcal{S}(\alpha) = \mathcal{S}_0(\alpha) + \varepsilon^2 \frac{\mathrm{d}\beta}{\mathrm{d}\alpha} \frac{\beta}{m_0 n} \left|\boldsymbol{\xi'}\right|^2 \tag{S6.9}$$

where $\mathcal{S}_0(\alpha)$ is the selection gradient in a homogeneous metapopulation. This formula (4) is the same as what we already have if we replace $\boldsymbol{\xi'}$ by $\boldsymbol{S^{*\prime}}$. After the replacement of $\boldsymbol{S^{*\prime}}$) and $\boldsymbol{S^{*\prime\prime}}$ by $\boldsymbol{\xi'}$ and $\boldsymbol{\xi''}$, we just need to repeat the same procedure as in our paper to derive Eq. (S6.8) and Eq. (S6.9).

To conclude, all our findings derived under mass-action type epidemiological dynamics are extended to frequency-dependent transmission models.

S6.1.2. Numerical evolution with frequency-dependent transmission. Our results on the evolution of virulence and infectiousness in heterogeneous metapopulation described in the main body and SI Appendices S1-S5 remain the same by simply replacing all local equilibrium susceptible densities, S_i^*, by equilibrium susceptible proportions, $\xi_i = S_i^*/N_i^*$, in local populations as already shown analytically in S6.1.1. We here confirm this analytically shown result by numerical evolutions in metapopulations where movement rates between local populations are drawn randomly. For simplicity, the parameters other than movement rates are kept constant over local populations.

We find that metapopulation heterogeneity in movement always increases the pathogen virulence (Fig 7a in the main body) and the increment of pathogen virulence is proportional to the variance of heterogeneity in movement (Fig 7d in the main body).

S6.2. Numerical evolution in superinfection model. We next numerically examine the effect of the metapopulation heterogeneity on the model with superinfection which is known to increase the ESS virulence in a single population (1–3). Here, we introduce the metapopulation heterogeneity of movement rate into the superinfection model described in previous study Nowak and May (3). The epidemiological dynamics is written as

$$\frac{\mathrm{d}S_i}{\mathrm{d}t} = r_0 - \mu S_i - S_i \sum_{k=1}^{h} \beta_k I_{i,k} + \frac{1}{n}\sum_j \widetilde{m}_{ij} S_j - \frac{1}{n}\sum_{j=1}^{n} \widetilde{m}_{ji} S_i, \qquad \text{[S6.10a]}$$

$$\frac{\mathrm{d}I_{i,k}}{\mathrm{d}t} = \left(\beta_k S_i + s\beta_k \sum_{l=1}^{k-1} I_{i,l} - s \sum_{l=k+1}^{h} \beta_l I_{i,l}\right) I_{i,k} - (\mu + \alpha_k) I_{i,k} + \frac{1}{n}\sum_j \widetilde{m}_{ij} I_{j,k} - \frac{1}{n}\sum_j \widetilde{m}_{ji} I_{i,k}, \qquad \text{[S6.10b]}$$

where S_i is the susceptible host density in local population i, $I_{i,k}$ is the density of hosts in local population i infected by pathogen genotype k, and β_k and α_k are the transmission rate and virulence of pathogen genotype k. It is assumed that pathogen genotype k can superinfect (take over), with probability s, the host already infected by genotype l if the genotype k is more virulent to hosts than genotype l ($k > l$).

We conducted the evolutionary simulation with randomly generated metapopulations with heterogeneity in movement rate. We assume the same parameter values and transmission-virulence tradeoff as assumed in (3) except movement rates which are introduced here to extend the model into heterogeneous metapopulation. As shown in Nowak and May (3), there is no stable evolutionary equilibrium and the mean virulence among metapopulation fluctuates with time (Fig. 6 of Nowak and May (3)). Thus, we calculated the mean virulence among metapopulation sampled at 1000 time points between $t = 10000$ and $t = 100000$ and averaged them. Fig. 7b in the main body shows that the time-averaged virulence with heterogenous movement is always larger than that in a homogeneous metapopulation and that the increment of virulence lineally increases with the variance of heterogeneity (Fig. 7e in the main body). Although the average virulence is highly scattered, perhaps because the evolutionary dynamics of virulence follow a very complex trajectory, as shown by Nowak and May (3), we conclude that our key results robustly hold with superinfections.

S6.3. Numerical evolution with density-dependent host mortality. Here we examine the effect of metapopulation heterogeneity on the evolution of pathogen virulence when evolutionary branching can occur in virulence in the models with more than one environmental feedback dimension, e.g. with a density-dependent host mortality (4, 5). We introduce the heterogeneity in movement rate into the model described in Svennungsen and Kisdi (5) with the parameter conditions under which evolutionary branching can occur and the evolutionary dimorphism can emerge. The model is described as

$$\frac{\mathrm{d}S_i}{\mathrm{d}t} = r_0 N_i - \beta S_i I_i - \mu(N_i) S_i + \frac{1}{n}\sum_j \widetilde{m}_{ij} S_j - \frac{1}{n}\sum_j \widetilde{m}_{ji} S_i, \qquad \text{[S6.11a]}$$

$$\frac{\mathrm{d}I_i}{\mathrm{d}t} = \beta S_i I_i - (\mu(N_i) + \alpha) I_i + \frac{1}{n}\sum_j \widetilde{m}_{ij} I_j - \frac{1}{n}\sum_j \widetilde{m}_{ji} I_i, \qquad \text{[S6.11b]}$$

In this model, the natural mortality of hosts depends on the total density of hosts, $N_i = S_i + I_i$, and the dependence is described as $\mu(N) = A + BN$. Additionally, they use the tradeoff function of the transmission rate and the virulence as $\beta(\alpha) = \frac{c\alpha}{a+\alpha}\left[1 - K\exp\left(-(\alpha - \tilde{\alpha})^2/\sigma^2\right)\right]$ which has the flexibility of its convexity by manipulating the value of K. We calculated the mean virulence in the population because, in the parameter conditions used, an evolutionarily stable dimorphism with low and high virulence genotypes can emerge in the homogeneous case. In the numerical evolutions with randomly generated movement heterogeneities, we find that the average virulence in metapopulation always increases by introducing the heterogeneity in movement (Fig. 7c in the main body) and that the relative increment of virulence is monotonically increased as increasing the degree of heterogeneity (Fig. 7f in the main body). Examining the change in the frequency of the two morphs as well as the mean virulence of each morph, we found that as the coefficient of variation of heterogeneity increases, the frequency of low virulence morphs decreases and that of high virulence morphs increases, while the mean virulence value of each morph was insensitive to the changes in the degree of metapopulation heterogeneity (Fig. S5). Above a certain threshold degree of heterogeneity, low virulence morph became extinct leaving a single morph with a high virulence. After the extinction of the low virulent morph, the virulence of the high virulent morph started to increase in response to the increase in the degree of heterogeneity, the rate of increase is slower than in the response by morph frequencies (zoomed-in panel in Fig. S5b).

In conclusion, the analytical and numerical results of the frequency-dependent transmission model, as well as the numerical results of the superinfection and density-dependent mortality models, all show that our key finding that the metapopulation heterogeneity always increases the pathogen virulence is robust in various epidemiological models.

Supplementary Figures

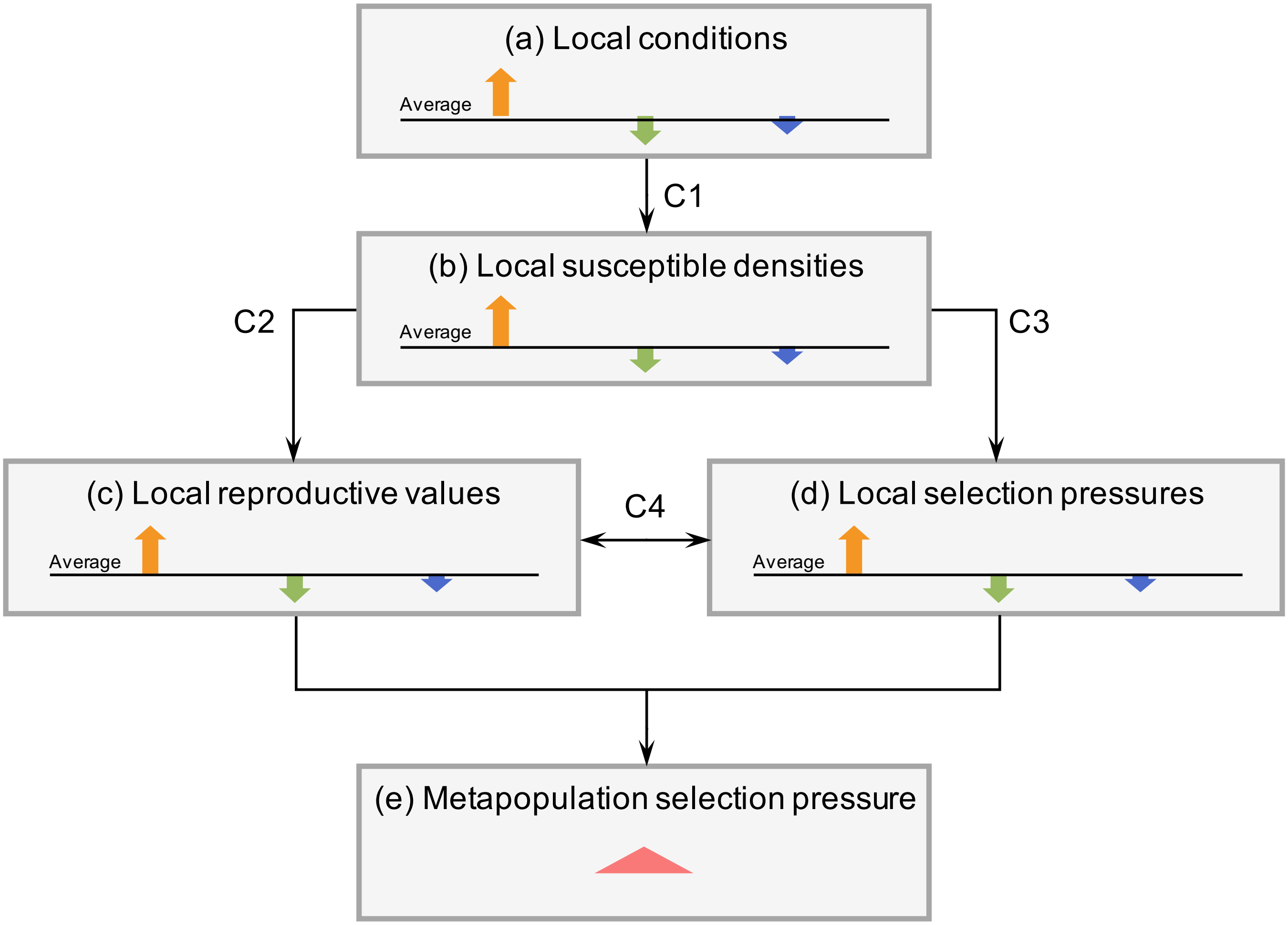

Fig. S1. Schematic summary of the fourfold quantitative concordance characterizing the evolution of pathogens in heterogeneous host metapopulations. (a) The local net movement rates, birth rates, carrying capacities, and immunity-loss rates of hosts may vary among local host populations and thereby deviate (as indicated by the orange, green, and blue arrows) from their metapopulation averages (as indicated by the black horizontal lines). The first quantitative concordance (C1) arises between the pattern of deviations in the local conditions (b) and that of deviations in the local densities of susceptible hosts (c). The second quantitative concordance (C2) arises between the pattern of deviations in the local densities of susceptible hosts (b) and that of deviations in the local reproductive values. The third quantitative concordance (C3) arises between the pattern of deviations in the local densities of susceptible hosts (b) and that of deviations in the local selection pressures (d). The fourth and last quantitative concordance (C4), implied by the concordances C2 and C3, arises between the pattern of deviations in the local reproductive values (c) and that of deviations in the local selection pressures (d). This last concordance explains, as shown in Fig. 4, why selection in heterogeneous metapopulations always favors pathogens with raised ESS virulence and infectiousness.

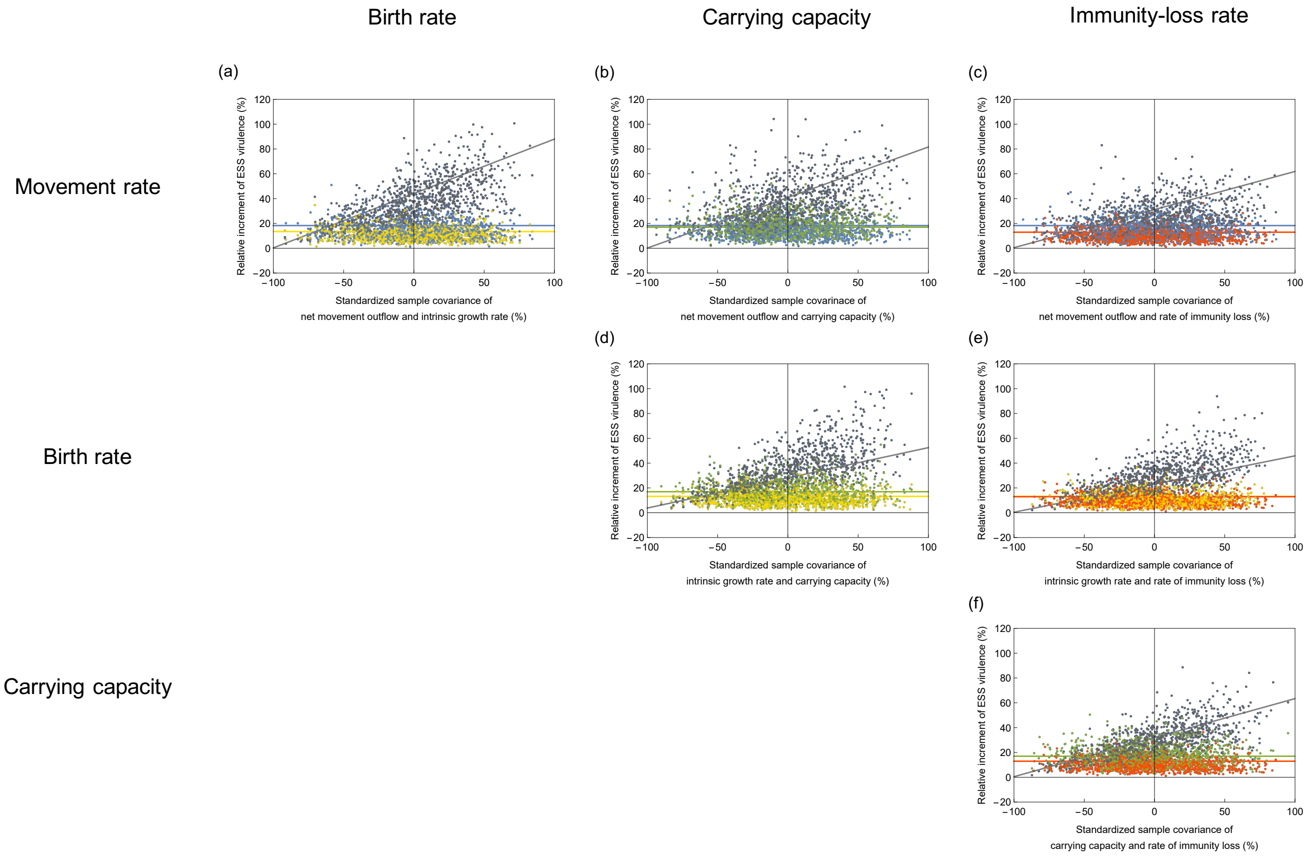

Fig. S2. Dependence of the relative increments of ESS virulence caused by two metapopulation heterogeneities on the covariation between these heterogeneities. The increments are measured in comparison with the ESS virulence in the corresponding homogeneous metapopulation. The figure compares numerical and analytical results for the six different combinations of four different types of heterogeneity: in (a) movement rates (first row), (b) birth rates (second row and first column), (c) carrying capacities (third row and second column), and (d) immunity-loss rates (third column). For each of the combinations of heterogeneities, the panels show on the vertical axes the relative increments of ESS virulence (grey dots) obtained through numerical evolution in each of 1,000 randomly generated metapopulations (created as described in the caption of Fig. 2) in comparison with the ESS virulence predicted by our analytical theory (grey lines) according to Eq. (5a). All metapopulations comprise n=20 local populations. The covariation between the two considered metapopulation heterogeneities, indicated on the horizontal axes, is measured by the relative sample covariance of the two heterogeneities. For comparison, the panels also show the relative increments of ESS virulence when the heterogeneity indicated in the corresponding row applies alone (dots of corresponding colors: blue for movement rates, yellow for birth rates, green for carrying capacities, and red for immunity-loss rates) and when the heterogeneity indicated in the corresponding column applies alone (dots of corresponding colors) in comparison with the ESS virulence predicted by our analytical theory (lines of corresponding colors) according to Eq. (2). The relative increment $\delta\alpha^*/\alpha_0^*$ of ESS virulence is minimized at the maximum negative correlation (i.e., at a relative sampling covariance of –100%, which implies $\delta\alpha^*/\alpha_0^* = \Theta\left(q_x\hat{V}_x^{1/2} - q_y\hat{V}_y^{1/2}\right)^2$): therefore, $\delta\alpha^*/\alpha_0^*$ is positive except when the two heterogeneities exactly cancel each other, $q_x\hat{V}_x^{1/2} = q_y\hat{V}_y^{1/2}$. Similarly, $\delta\alpha^*/\alpha_0^*$ is maximized at the maximum positive correlation (i.e., at a relative sampling covariance of 100%, which implies $\delta\alpha^*/\alpha_0^* = \Theta\left(q_x\hat{V}_x^{1/2} + q_y\hat{V}_y^{1/2}\right)^2$. All parameter values are as shown in Table 1.

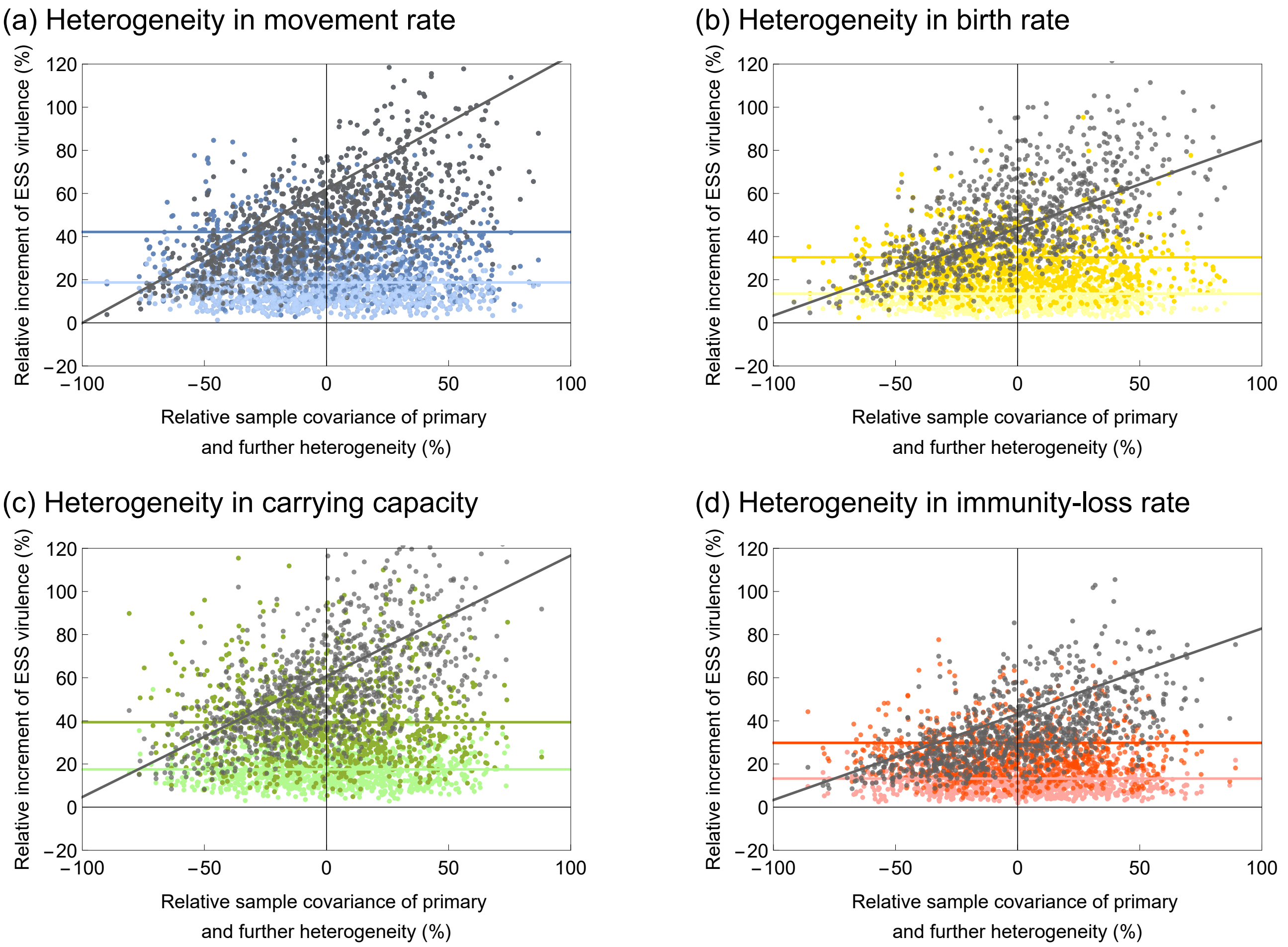

Fig. S3. Dependence of the relative increments of ESS virulence caused by additional heterogeneity in an already heterogeneous metapopulation on the covariation between these heterogeneities. The increments are measured in comparison with the ESS virulence in the corresponding homogeneous metapopulation. The degree of heterogeneity is 15% for the primary heterogeneity and 10% for the further heterogeneity. The figure compares numerical and analytical results for the four different types of heterogeneity: in (a) movement rates, (b) birth rates, (c) carrying capacities, and (d) immunity-loss rates. For each heterogeneity, the panels show on the vertical axes the relative increments of ESS virulence (grey dots) obtained through numerical evolution in each of 1,000 randomly generated metapopulations (created as described in the caption of Fig. 2) in comparison with the ESS virulence predicted by our analytical theory (grey lines) according to Eq. (5c). All metapopulations comprise $n = 20$ local populations. The covariation between the two considered metapopulation heterogeneities, indicated on the horizontal axes, is measured by the relative sample covariance of the two heterogeneities. For comparison, the panels also show the relative increments of ESS virulence when the primary heterogeneity applies alone (dots of corresponding colors) and when the further heterogeneity applies alone (dots of corresponding light colors) in comparison with the ESS virulence predicted by our analytical theory (horizontal lines of corresponding colors) according to Eq. (2). The relative increment $\delta\alpha^*/\alpha_0^*$ of ESS virulence is minimized at the maximum negative correlation (i.e., at a relative sampling covariance of -100%, which implies $\delta\alpha^*/\alpha_0^* = \Theta q_x^2 \left(\hat{V}_x^{1/2} - \hat{V}_{\tilde{x}}^{1/2}\right)^2 = (1/9)\Theta q_x^2 \hat{V}_x$): since we assume $\hat{V}_{\tilde{x}}^{1/2} = (2/3)\hat{V}_x^{1/2}$, $\delta\alpha^*/\alpha_0^*$ is reduced to one-ninth of the value applying with only the primary heterogeneity. Similarly, $\delta\alpha^*/\alpha_0^*$ is maximized at the maximum positive correlation (i.e., at a relative sampling covariance of 100%, which implies $\delta\alpha^*/\alpha_0^* = \Theta q_x^2 \left(\hat{V}_x^{1/2} + \hat{V}_{\tilde{x}}^{1/2}\right)^2 = (25/9)\Theta q_x^2 \hat{V}_x$): since we assume $\hat{V}_{\tilde{x}}^{1/2} = (2/3)\hat{V}_x^{1/2}$, $\delta\alpha^*/\alpha_0^*$ is increased to about 2.8 times of the value applying with only the primary heterogeneity. All parameter values are as shown in Table 1.

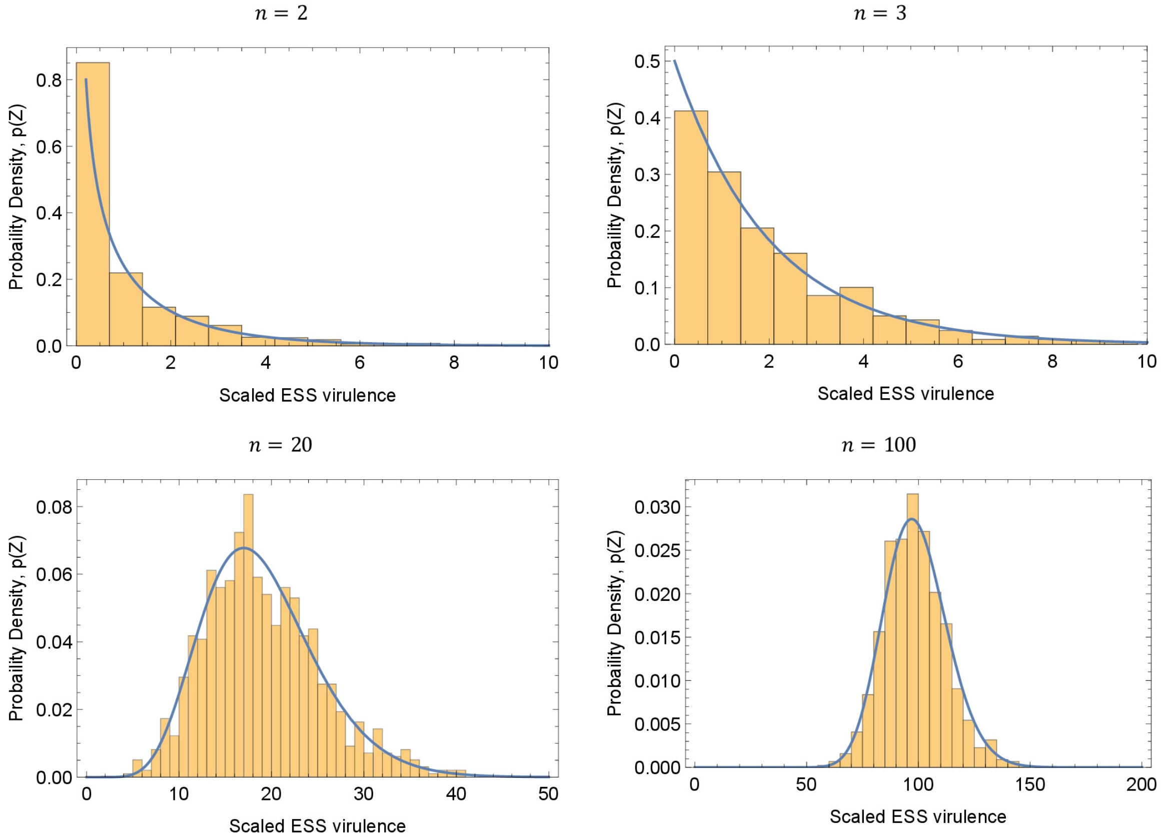

Fig. S4. The predicted and observed distribution of the scaled ESS virulence increment in a heterogeneous metapopulation in which movement rates between local populations are varied. In each panel, the observed distribution for the scaled ESS virulence increment, $Z = (\delta\alpha/\alpha_0^*)(n^2/2\varepsilon^2\mathcal{E}_T)$, is shown as a histogram, and the predicted chi-square distribution is shown as the solid curve. The movement rates, $m_{ij} = m_0 + \varepsilon m'_{ij}$, from local population j to i $(i, j = 1, \ldots, n)$ are varied randomly around its mean $m_0 = 0.6$, where m'_{ij} $(i, j = 1, \ldots, n)$ are mutually independent normal random deviates with standard deviation $\sigma_m = \varepsilon m_0 = 0.06$. The degree of heterogeneity in movement rates is set to be $\varepsilon = 0.1$. The number of local populations consisting of a metapopulation is varied as $n = 2$, 3, 20, and 100. For each panel, 1000 independent heterogeneous metapopulations are generated to make the observed distribution for the increments of the evolved virulence. The solid curves are the predicted probability density function for Z: chi-square distribution with the degree of freedom $n - 1$. See Table 1 for the other parameters.

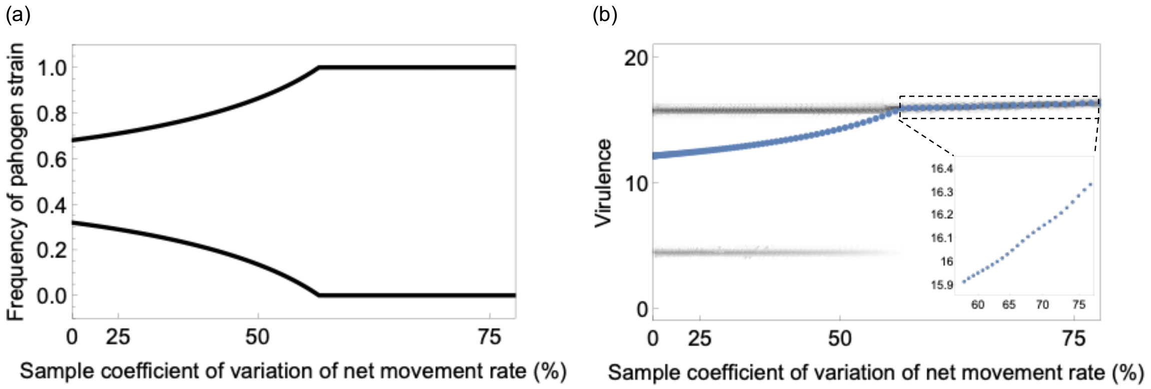

Fig. S5. Dependence of evolutionarily stable distribution of pathogen virulence trait on the degree of metapopulation heterogeneity when evolutionary branching can occur in homogeneous population. We randomly sampled m'_{ij} once and only changed ε for every simulation to generate heterogeneous metapopulations with different degrees of heterogeneity. (a) Relative frequency of dimorphic pathogen strains plotted against the sampled coefficient of variation of net movement rate. As the degree of heterogeneity increases, the frequency of high virulence morph increases (and that of low virulence morph decreases), while their within morph distributions are kept nearly constant. As the degree of heterogeneity further increases, low virulence morph becomes extinct and dimorphism is no longer maintained. (b) Heat map of virulence distribution (gray shades) and population mean virulence (blue points) plotted against the sampled coefficient of variation of net movement rate. The within-morph mean virulences of both morphs do not change as the degree of heterogeneity increases until the low virulence morph becomes extinct, but the population mean virulence increases as the frequency of high virulence morph increases and that of low virulence morph decreases. After the extinction of the low virulence morph, the mean virulence of the high virulent morph begins to increase. The parameter values and the tradeoff function are the same as used in Fig. 7c and 7f in the main body.

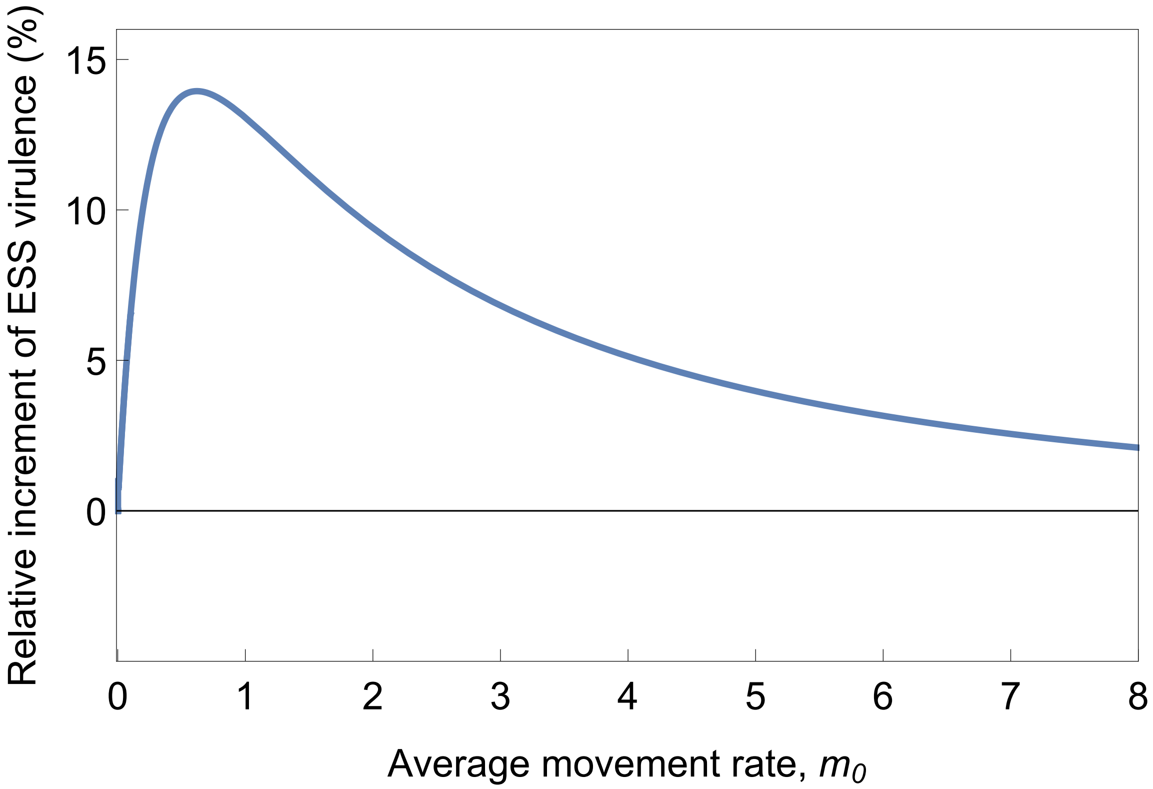

Fig. S6. Dependence of the relative increment of ESS virulence caused by metapopulation heterogeneity in movement on the average movement rate m_0. We evaluate the ESS virulence when only the movement is heterogeneous, with fixed values for all parameters except m_0. The ESS virulence is maximized at intermediate values of m_0 and gets smaller as m_0 approaches 0 or infinity, where it naturally approaches the ESS expected in a homogeneous population. All parameter values except m_0 are the same as in Fig. 2 in the main text, and the coefficient of variation in movement rates equals $\varepsilon = 0.1$.

References

1. HJ Bremermann, J Pickering, A game-theoretical model of parasite virulence. *J. Theor. Biol.* **100**, 411–426 (1983).
2. A Sasaki, Y Iwasa, Optimal growth schedule of pathogens within a host: Switching between lytic and latent cycles. *Theor. Popul. Biol.* **39**, 201–239 (1991).
3. MA Nowak, RM May, Superinfection and the evolution of parasite virulence. *Proc. Royal Soc. London. Ser. B: Biol. Sci.* **255**, 81–89 (1994).
4. S Lion, JAJ Metz, Beyond R0 maximisation: on pathogen evolution and environmental dimensions. *Trends Ecol. & Evol.* **33**, 458–473 (2018).
5. TO Svennungsen, E Kisdi, Evolutionary branching of virulence in a single-infection model. *J. Theor. Biol.* **257**, 408–418 (2009).
